# Low Salt Influences Archaellum-Based Motility, Glycerol Metabolism, and Gas Vesicles Biogenesis in *Halobacterium salinarum*

**DOI:** 10.3390/microorganisms10122442

**Published:** 2022-12-10

**Authors:** Evelyn Ayumi Onga, Ricardo Z. N. Vêncio, Tie Koide

**Affiliations:** 1 Department of Biochemistry and Immunology, Ribeirão Preto Medical School, University of São Paulo, Ribeirão Preto 14049-900, Brazil; 2 Department of Computation and Mathematics, Faculdade de Filosofia, Ciências e Letras de Ribeirão Preto, University of São Paulo, Ribeirão Preto 14040-901, Brazil

**Keywords:** salt stress response, *Halobacterium salinarum* NRC-1, transcriptome proteome comparison, low salt

## Abstract

*Halobacterium salinarum* NRC-1 is an extremophile that grows optimally at 4.3 M NaCl concentration. In spite of being an established model microorganism for the archaea domain, direct comparisons between its proteome and transcriptome during osmotic stress are still not available. Through RNA-seq-based transcriptomics, we compared a low salt (2.6 M NaCl) stress condition with 4.3 M of NaCl and found 283 differentially expressed *loci*. The more commonly found classes of genes were: ABC-type transporters and transcription factors. Similarities, and most importantly, differences between our findings and previously published datasets in similar experimental conditions are discussed. We validated three important biological processes differentially expressed: gas vesicles production (due to down-regulation of *gvpA1b*, *gvpC1b*, *gvpN1b*, and *gvpO1b*); archaellum formation (due to down-regulation of *arlI*, *arlB1*, *arlB2*, and *arlB3*); and glycerol metabolism (due to up-regulation of *glpA1*, *glpB*, and *glpC*). Direct comparison between transcriptomics and proteomics showed 58% agreement between mRNA and protein level changes, pointing to post-transcriptional regulation candidates. From those genes, we highlight *rpl15e*, encoding for the 50S ribosomal protein L15e, for which we hypothesize an ionic strength-dependent conformational change that guides post-transcriptional processing of its mRNA and, thus, possible salt-dependent regulation of the translation machinery.

## 1. Introduction

*Halobacterium salinarum* NRC-1 is an extremely halophilic archaeon, whose optimal salt growth concentration is 4.3 M of NaCl (~0.825 water activity) [1,2]. There are extremophiles that can grow at saturated solutions (0.755 water activity), including *H. salinarum* NRC-1 that withstands growth even beyond that of saturated NaCl (0.717 water activity) being one of the top 10 most halophilic microbes [2,3]. The high salinity intrinsic to such a lifestyle has implications for osmotic pressure and protein folding [4,5]. An adaptation mechanism this microorganism uses to survive consists of maintaining high levels of internal potassium chloride in addition to presenting a proteome with a median pI of 4.4, with many negatively charged amino acids on its protein’s surface and overall rigidity of protein folding [5,6,7,8].

Previous studies on salt stress in halophiles show that submitting these cells to a higher concentration of salt is less disruptive to the metabolism than lower salt concentrations, given the smaller number of genes differentially expressed and their functions in the organism [9,10]. At higher salt concentrations, proteins are stabilized by a solvation layer formed around charged amino acids [11,12,13,14], but the process of adaptation to lower salinity is not very clear.

Recently, it was shown that many large-scale changes happen at a molecular level when *H. salinarum* NRC-1 is under salt concentrations lower than its optimal [8,11,15]. For instance, cell shape distributions change towards great circularity at 3.4 M NaCl [15]. Cells growing under 2.5 M had their growth rates five to eight times lower than optimal, and flow cytometry experiments indicated an increase in the percentage of membrane and DNA damage when salt concentration decreased. Neutron scattering experiments and respiration activity experiments showed that low salt alters the molecular dynamics of *H. salinarum*’s proteome and reduces the metabolic activity of the cells [8,11].

Given its importance as an extremophile model organism, there are seminal published studies on osmotic stress in *H. salinarum* NRC-1 [9,10]. These studies used DNA microarrays for transcriptome and mass spectrometry for proteome profiling, the state-of-the-art ‘omics’ technologies ~15 years ago. DNA microarray results indicated that there was a cellular metabolism restructuring to deal mainly with the transport of ions, phosphate sulfur, and maintenance of intracellular ionic levels [10]. On the other hand, the study of the proteome showed the rearrangement of cell metabolism to obtain more energy, maintenance of already formed proteins, and DNA repair [9]. These and many other studies [8,15,16,17,18,19] lay the foundation for understanding better mechanisms of halophilic archaea adaptation and survival in different salt conditions. However, to the best of our knowledge, there are still no direct comparisons between transcriptomic and proteomic *H. salinarum* NRC-1′s informational layers at (approximately) the same experimental conditions mimicking low salt environments.

In the present work, we performed a transcriptome survey at 2.6 M NaCl, a low salt osmotic stress condition for *H. salinarum* NRC-1, and at 4.3 M NaCl control condition in which this extremophile grows optimally. Proteome data are publicly available for the same conditions [9], which allowed us to perform a direct comparison and identify candidates for post-transcriptional regulation.

## 2. Materials and Methods

### 2.1. Strain, Growth Conditions, and RNA Extraction

*H. salinarum* NRC-1 was cultivated in complex medium (CM; 250 g/L NaCl, 20 g/L MgSO_4,_ 2 g/L KCl, 3 g/L Sodium Citrate, 10 g/L Bacteriological Peptone Oxoid) in liquid or solid medium (adding 1.5% *w*/*v* agar). Colonies of *H. salinarum* NRC-1 were selected from Petri dishes, and pre-inoculum was grown in liquid medium under light and stirring at 160 rpm. After five days, cultures were diluted to an optical density (OD_600_) of 0.05 in medium with 4.3 M NaCl (synonymous: control, optimal, O, 25% *w*/*v*) and 2.6M NaCl (hyposaline, low salt, L, 15% *w*/*v*), as described by Leuko et al. (2009) [9]. Growth curves were performed in duplicates at 37 °C, under light and agitation at 160 rpm, and OD_600_ was measured every 24 h. When samples reached OD_600_ of 0.5, the culture was centrifuged at 10,000× *g* for five minutes. Pellets were stored at −80 °C. RNA extraction was performed using the protocol adapted from the mirVana™ miRNA Isolation kit (Ambion). A control PCR was performed using primers for VNG_RS06465 to check for DNA contamination using primers: (forward) CCC GAG AAG TTC ACG CGG and (reverse) GCT GCC CTG GCG GCT GTT). Agarose gels (1% *w*/*v*) and Bioanalyzer 2100 Expert (Agilent Technologies, Santa Clara, CA, USA—Agilent RNA 6000 Nano kit) were used to verify RNA integrity. 

### 2.2. RNA-seq Library Preparation, Sequencing, and Data Analysis

Ribosomal RNA was depleted using the RiboZero kit (Illumina, San Diego, CA, USA). Illumina TruSeq Stranded mRNA Kit protocol was started with 330 ng of RNA for each sample. Quality control of the libraries was performed with Bioanalyzer 2100 Expert (Agilent Technologies) using the Agilent High Sensitivity DNA kit and Qubit 4 Fluorometer (Thermo Fisher Scientific, Waltham, CA, USA) with the Qubit™ dsDNA HS Assay kit. Libraries were sequenced using MISeq Reagent Kit v2 kit (Illumina) of 50 cycles in a paired-ended mode in MISeq 03081 (Illumina) at Hemocentro FMRP-USP, University of São Paulo Ribeirão Preto. Four libraries were sequenced, with two biological replicates for each treatment. The fastq files generated were submitted to frtc (https://github.com/alanlorenzetti/frtc (accessed on 21 October 2019 )), an automatic in-house pipeline assembled to perform the RNA-seq analysis of prokaryotes closely following a previously published protocol [20]. Experiments were compared in terms of logarithmic fold-change between the low salt (L) condition and the optimum control condition (O) as M = log_2_(L/O) with proper additive normalization to center mean transcriptome M values around <M> = 0. Differentially expressed genes were defined using the HTself method [21], which can handle statistical analysis with few replicates modeling the random noise with self-self experiments (replicate vs. replicate in this work) after minor adaptations to handle RNA-seq data. Genes presenting M = log_2_(fold-change) values outside 95% probability cutoffs in all biological replicates were considered differentially expressed (data-driven cutoffs of M < −1.4 and M > 1.3). Differentially expressed genes were bulk analyzed for global patterns using standard enrichment and functional analysis tools made available at PANTHER knowledge base [22] (http://pantherdb.org/ (accessed on 29 September 2022)). 

### 2.3. Proteomics and RNA-seq Data Comparison

Differentially expressed proteins were filtered from published data by Leuko et al. (2009) [9] considering the same numerical M value filters defined previously for RNA-seq data. Genes for which protein levels did not considerably change or were down-regulated, not following an mRNA up-regulation upon stress, were considered post-transcriptional regulation candidates. These highlighted cases were further investigated in silico using: (i) visual inspection of their predicted RNA secondary structure obtained by RNAbow [23] and RNAfold [24], and (ii) visual inspection of ribosome footprint profile (Ribo-seq) data and Transcript Processing Sites (TPS) data intersections [25].

### 2.4. Growth Curve Supplemented with Glycerol

Control and low-salinity media were supplemented with 30 µg/mL and 60 µg/mL glycerol, and growth curves were made in triplicate with OD_600_ measurements every 3 h. Glycerol concentrations used were based on [26], which shows that *Dunaliella salina* produces an average of 30 µg/mL in different salinity levels, and at a concentration of 2 M NaCl, it has its maximum production of approximately 60 µg/mL.

### 2.5. Motility Assays

Motility assays were performed as previously described [27,28]: Liquid culture with O.D. in the logarithmic growth phase (0.5) was inoculated in the center of a petri dish with semi-solid CM 0.3% *w*/*v* agar. The plates were incubated for five days at 37 °C, and then the diameter of the motility halo was measured. When the halo was deformed, the average between the smallest and largest radius was taken.

### 2.6. Gas Vesicles Evaluation

After completion of the growth curves previously described, the cultures were left on the bench without agitation, and pictures were taken after 120 h.

## 3. Results

This section presents an overall view of the obtained results as objectively as possible, redirecting our interpretation to the next section. We start with an overview of the transcriptome changes and differentially expressed genes, with detailed data tables as supplemental material. Next, we highlight two biological processes found down-regulated in low salt stress and then one up-regulated in the following sub-sections. We close with a transcriptome/proteome direct comparison. Genes are identified by their common symbols but their exact annotations, *loci* positions, and IDs can be easily traced back in the Supplemental Materials (Table S1, sheets e and g).

### 3.1. A Global Transcriptomic Survey in Low Salt

To expand our understanding of how osmotic stress affects the gene expression of *H. salinarum* NRC-1, we performed a global survey of differential gene expression using RNA-seq. The conditions investigated were specifically chosen to be physiologically relevant and compatible with published legacy data on protein expression [9], so novel insights could be derived: media containing NaCl optimal growth concentration (4.3 M) and media containing a relatively low concentration of NaCl (2.6 M).

In this study, growth curves recapitulate previously known differences. Literature shows that under low salinity (2.5 M NaCl), around 80% of the cells were intact and 20% were ruptured [8,11], along with lower OD_600_ along the growth curve (Figure S1). In addition, cells incubated with 2.5 M of NaCl medium could undergo a reactivation process and continue growing in terms of cell density when re-incubated in the optimal growth medium [11]. We reproduced these established observations as a quality control standard for the conditions assessed in our transcriptome analysis (Figure S2). In spite of rRNA depletion procedures, the percentage of rRNA in the final samples were, at most, 40%, 13% and <0.01% for 23S, 16S, and 5S rRNAs, respectively. Inefficient rRNA depletion represent a somewhat waste of sequencing resources, reducing effective sequencing depth, but do not influence the results. 

Our transcriptome analysis showed **283** *loci* classified as differentially expressed under low salinity stress. Of those, 113 were up-regulated (L>O) and 170 were down-regulated (O>L) under osmotic stress (Table S1, sheet e). The RNA-seq approach allows feature identification beyond classically annotated genes, therefore the 283 *loci* include 105 coding sequence genes (CDS) up-regulated; 163 CDS down-regulated, and 15 non-coding RNAs (ncRNAs) were differentially expressed in this condition, 11 out of which were identified in the present work (Table S1, sheet e) and four are antisense RNA (asRNAs) [29].

For instance, the top three annotated genes whose expression level increased in low- salt conditions are *ycdH*, *queC*, and *repI*, encoding an ABC-type metal ion transport protein (20-fold), a ligase involved in queuosine synthesis (13-fold), and a plasmid replication protein (12-fold), respectively. Conversely, *acs*, *dmsB*, and *dmsC*, encoding an acyl-CoA synthetase (45-fold) and dimethylsulfoxide reductase subunits B (34-fold) and C (32-fold), respectively, were the top three genes showing the strongest decrease in expression under stress. The complete gene list, along with direct links to the *H. salinarum* NRC-1 Atlas (https://halodata.systemsbiology.net/ (accessed on 9 June 2022 )) [30] for each gene, is available in Supplementary Table S1 (sheet e).

From a global point of view, the most common protein families encoded by the differentially expressed genes were ABC-type transporters (12 genes); dehydrogenases, oxidoreductases, and ligases (more than five genes), and transcription factors (five genes), among many other single hits into PANTHER families, all available in detail as a supplemental file (Supplementary File S1). A random uniform drawing from a 283 gene list would be expected to return only five genes classified, as in the ATP-binding cassette transporter family, but we observed 12, approximately twice as much (PANTHER PC00003, *p*-value < 0.015), showing its overrepresentation.

Genes related to DNA repair such as excinuclease *uvrA* and endonuclease III, and transcription regulators such as *rosR*, *dmsR*, *sirR*, and *boa4* were altered in low salt. We also saw perturbations in genes related to translation: tRNA modification, ribosomal proteins, and TATA-box binding proteins which might contribute to overall proteome dynamics, an important factor for hyposalinity survival [8]. Cell division-related genes were down-regulated, and remodeling of cell surface glycoproteins was observed. Low respiration rate was previously reported in hyposalinity [11] and could be a consequence of the low expression of complex IV in the electron transport chain seen in our data. 

Our RNA-seq experiments showed changes in *H. salinarum* NRC-1 RNA levels for three important biological processes: (i) gas vesicles production (*gvpA1b*, *gvpC1b*, *gvpN1b*, and *gvpO1b*), (ii) archaellum formation (*arlI*, *arlB1*, *arlB2*, and *arlB3*), and (iii) glycerol metabolism (*glpA1*, *glpB*, and *glpC*). We decided to further investigate these three processes by performing phenotypic evaluations.

### 3.2. Gas Vesicle Biogenesis and Cell Motility Are Down-Regulated on Low Salinity Stress

Given the at least 4-fold decrease in RNA expression of many gas vesicle biogenesis genes, we conducted a follow-up validation to observe the consequences of a controlled low salt environment on *H. salinarum* NRC-1 phenotype. 

**Figure 1:**
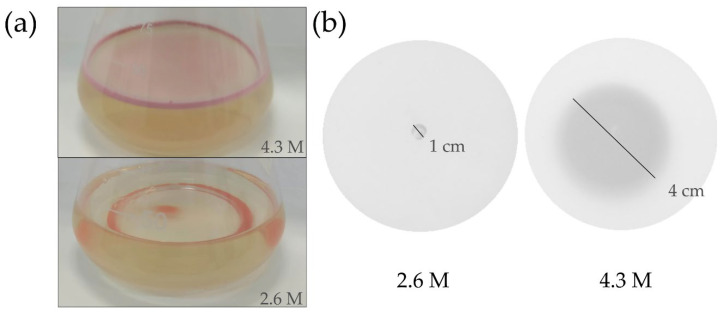
Buoyancy and motility phenotypes were observed in salt-stressed cultures. The optimum growth condition is 4.3 M NaCl and low salt is 2.6 M NaCl. (**a**) Fluctuation of cells in optimal salinity and sinking of cells in hyposalinity. (**b**) A motility assay showed less motility of cells in hyposalinity.

To evaluate the gas vesicles’ presence, we observed 7-day-old cultures left on the bench for 120 h. Cultures at 4.3 M NaCl displayed characteristic pink biofilm with floating cells while cultures at 2.6 M NaCl showed cells sinking at the bottom of the flask, with reddish color (Figure 1a). These results are consistent with the down-regulation of the *gvp* cluster at low salinity seen in our data, as well as mutants that have no gas vesicle production [31].

Along with the gas vesicles, another motility structure used by *H. salinarum* NRC-1 is the archaellum, a flagellum-like structure that allows swimming [32]. Given the average 4-fold decrease in RNA expression of genes involved in the archaellar motility under low salt stress, we conducted a follow-up validation expecting to observe motility impairment. Archaellin-based cell motility was evaluated by inoculating cells in semi-solid media with 2.6 M or 4.3 M NaCl and measuring the growth halo. Growth of 4 cm was observed in the control condition, while less than 1 cm was observed in low salinity (Figure 1b), a phenotype consistent with the down-regulation of archaellin-related transcript levels.

### 3.3. Glycerol Metabolism Is Up-Regulated in Low Salinity

Differential expression analysis revealed that *glpA*, *glpB*, and *glpC* genes, which code for the three subunits of the enzyme glycerol-3-phosphate dehydrogenase, were more expressed in hyposalinity. The average induction was 6-fold under low salt stress, which motivated validation experiments with glycerol supplementation to observe phenotypical responses under this stressed environment.

**Figure 2:**
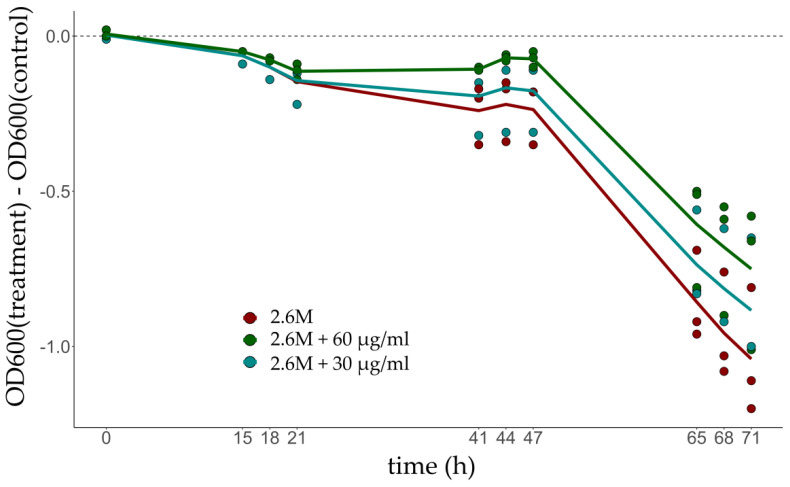
Phenotype comparison in salt-stressed cultures supplemented with 30 μg (blue) and 60 μg glycerol (green). Optimum growth condition is 4.3 M NaCl (control) and low salt is 2.6 M NaCl (treatment). Supplementation resulted in better growth of cells in hyposalinity. OD_600_ stands for optical density at 600 nm.

Differences in OD_600_ observed at low salinity compared to control conditions show that glycerol supplementation has a positive effect on the growth rate of the culture, which is consistent with the up-regulation of glycerol-3-phosphate dehydrogenase and the use of glycerol as an energy source (Figure 2).

The positive impact on growth is clearly revealed around 65 h if 60 µg/mL of glycerol is added.

### 3.4. Comparison between Transcriptome and Proteome

We compared our RNA-seq dataset with published *H. salinarum* NRC-1 proteome results by Leuko et al. (2009) [9], given that the experiments were performed, by design, under very similar conditions. We detected transcripts for almost all (98%) proteins previously measured [9].

**Figure 3:**
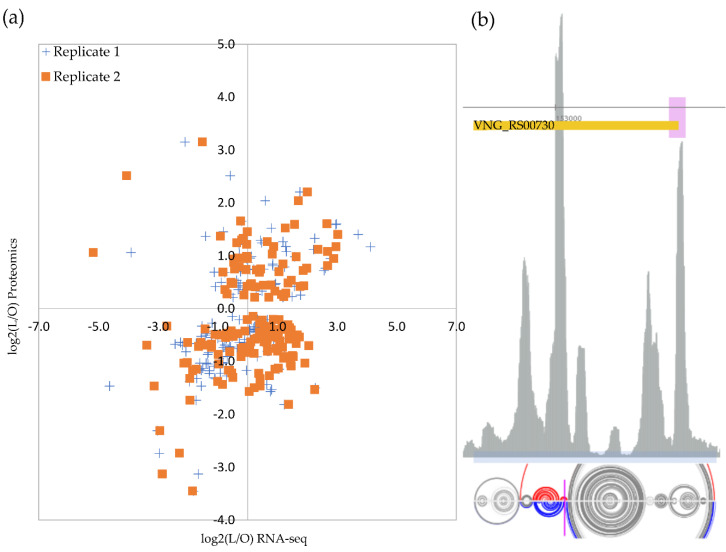
Transcriptome and proteome comparison. (**a**) Optimum growth condition is 4.3 M NaCl (O) and low salt is 2.6 M NaCl (L). Proteome data from Leuko et al. (2009) [9]. The relative number of genes in every four quadrants is shown. (**b**) Example of the putative post-transcriptionally regulated gene, 50S ribosomal protein L15e (*rpl15e*), and its Ribo-seq ribosome footprint profile (gray sequenced reads counting peaks in arbitrary scale). Ribo-seq data from Lorenzetti et al. (2022) [30]. The orange box is the annotated CDS with 5’-3’ direction shown from right to left. The pink box indicates the SmAP1 RNA chaperone binding site. Magenta marker indicates transcription processing site. The light blue box indicates the region used for RNA structure prediction with arch representations for the two thermodynamically stable predictions in the ensemble below (bases connected by an arch are predicted to be pairing, gray arches are similar pairing predictions in both structure models, and colored are different). See text for details.

As expected, not all genes follow a straightforward relationship between transcription and translation, but the majority (26% + 32%) showed a positive relation (Figure 3a). For the 165 proteins that were previously detected in hyposalinity, we analyzed whether our RNA-seq data indicated a similar direction of change, i.e., up- or down-regulated relative to optimal salinity.

The original work by Leuko et al. (2009) considered as up-regulated any reliably identified protein above a simple 1:1 ratio (M > 0). Conversely, they considered as down-regulated any case more expressed in low salt stress condition relative to the optimal growth condition (M < 0). We adopted more stringent criteria and applied, for simplicity, the same fold-cutoff used in our RNA-seq analysis. RNAs and proteins displayed strong similar changing patterns for ten genes: *dpp1A*, *arlA2*, *arlB2*, *arlB3*, *cbiC*, *sirR*, *sufS*, *nrdJ*, VNG_RS02360 *locus*, and VNG_RS03640 *locus*, suggesting a canonical transcription and translation coupling (Table S1, sheet g). These annotated genes code for, respectively: an ABC transporter substrate-binding protein, archaellins, a precorrin-8× methylmutase, a transcriptional regulator, a cysteine desulfurase, an adenosyl cobalamin-dependent ribonucleoside-diphosphate reductase, and two not characterized proteins.

However, for four cases, we observed discordant fold-changes patterns: *rpl15e*, a 50S ribosomal protein L15e (VNG_RS00730), and the uncharacterized *loci* VNG_RS09060, VNG_RS05135, and VNG_RS07000; being up- and down-; up- and down-; down- and up-, and down- and up-regulated at RNA and protein levels, respectively.

Transcript levels of gene *rpl15e* (encoding ribosomal protein L15e) more than doubled under low salt stress whereas the protein levels were detected as less than one third at the same condition (Table S1, sheet g), indicating some sort of salinity-induced post-transcriptional regulation of the translational machinery. Data mining of a published ribosome footprint profile (Ribo-seq) dataset [30], acquired from optimal growth conditions, detected a clear signal of ribosome arrest inside the *rpl15e* transcript (Figure 3b). This Ribo-seq peak is co-localized with a strong cleavage signal retrieved from our previously published database of transcript processing sites (TPS) [25]. Finally, there is evidence from the *H. salinarum* NRC-1 Atlas [30] that the *rpl15e* transcript’s 5′ portion binds to the archaeal equivalent to bacterial Hfq RNA chaperone, named SmAP1 (Figure 3b), which is consistent with post-transcriptional regulation evidence.

## 4. Discussion

### 4.1. Transcriptome Change under Hyposalinity Stress

Although *H. salinarum* NRC-1 is a long-established model organism for halophile microorganisms, we did not find multi-omics system-level studies comparing its osmotic stress response at both transcriptome and proteome levels. Therefore, we set up matching experimental conditions to legacy proteomics data [9] in order to investigate the respective transcriptome change under low salt stress (2.6 M) compared to optimal salt concentration (4.3 M).

Osmotic stress elicits many molecular and phenotypical responses in the organism, perturbing biological processes and players such as transcription regulators; translational machinery; cell division; energy production; locomotion and buoyancy, etc., all represented in the selected 283 genes (~11% of the ~2600-gene genome) with 113 up-regulated and 170 down-regulated in stress (Table S1, sheet e). 

Recently, a study with different goals used deep sequencing to explore osmotic stress in *H. salinarum* NRC-1 and, as an intermediate byproduct, compared optimal and low salt growth conditions [15]. Low salt was defined as 3.4 M NaCl, still, ~1 M higher than our work, and a non-wild-type strain (delta-*ura3* strain for uracil counter-selection) was used to allow genetic perturbation on the sole histone encoded in this model archaea. Nonetheless, interesting comparisons can be made (Table S1, sheet h). From all 145 genes with at least 1.5-fold change in all 2.6 M vs. 4.3 M replicates and, simultaneously, confidently reported by the 3.4 M vs. 4.3 M comparisons (Table S4 in [15]), 52% agree but 48% do not agree. Many overall classes and individual genes discussed in our result sections were found as well. Interesting disagreements include our highlighted gas vesicle genes: *gvpD*, *gvpE*, *gvpF*, *gvpG*, *gvpH*, *gvpI*, *gvpJ*, *gvpL*, and *gvpO* which were all less expressed in 2.6 M than 4.3 M (at least 1.7-fold, average 2.7-fold change) but were more expressed in 3.4 M than in 4.3 M (>1.6-fold change). Another noticeable disagreement is the gene *hop*, rhodopsin (VNG_RS00745 *locus*), which we found highly down-regulated but was observed 1.8-fold up-regulated in 3.4 M vs. 4.3 M. Reasons for disagreement can vary wildly from methodological differences to actual biological phase transitions happening between “extremely” low salt (2.6 M NaCl) to “less extreme” low salt (3.4 M). Further investigation is evidently required; however, this type of orthogonal replication is probably the most effective way to identify the universal hyposalinity adaptation mechanisms.

Some technical and arbitrary decisions are involved in establishing this list of the differentially expressed genes, however, we made the raw and processed data easily available to foster independent alternative interpretations and data mining (NCBI-SRA accession PRJNA674319). From all perturbations detected, we decided to follow up and validate a few: gas vesicle biogenesis down-regulation; cell motility down-regulation; glycerol metabolism up-regulation. Additionally, we investigated in silico putative post-transcriptional regulations due to transcriptome/proteome discrepancies.

### 4.2. Buoyancy and Swimming Impairment under Hyposalinity Stress

The gas vesicles, assembled by the proteins coded from the *gvp* operon genes, are hollow protein nano compartments filled with oxygen [31]. This structure gives cells the ability to migrate vertically to more oxygenated regions [33]. It is believed that this migration helps cells reach higher light intensities for the functioning of bacteriorhodopsin [34]. However, the correlation between gas vesicle production, bacteriorhodopsin, and light intensity is not totally clear. Proteomics studies under low salinity showed an up-regulation of bacteriorhodopsin [9], while in our study, the *bop* gene was found down-regulated by >16-fold. Our data showed >4-fold down-regulation of several gas vesicle-involved genes, thus, we expected to see a phenotype compatible with some sort of buoyancy impairment under stress, as we observed, validating this finding (Figure 1a).

*H. salinarum* NRC-1 also achieves mobility by swimming using its archaellum. The archaellum operon is composed of many proteins, including archaellin A1, A2, B1, B2, and B3, which are the proteins that make up the extracellular fraction of the archaellum, from the hook (B3) to the elongation of the archaellum (A1, A2, B1, B2) [35,36]. The rotation of the flagellar motor is ATP-dependent, and it is related to the transfer of signals coming from the sensory rhodopsins, one of them, the bacteriorhodopsin [28]. Previous data suggest that the down-regulation of bacteriorhodopsin in low salinity results in the down-regulation of the archaellum as a consequence [37,38]. Our findings showed that rhodopsins coded by *bob*, *hop*, and *sopI* are >4-fold down-regulated, along with actual archaellum-related genes *arlI*, *arlB1*, *arlB2*, and *arlB3* which led to the prediction that swimming would be impaired under low salt stress, as validated by a motility assay (Figure 1b).

### 4.3. Glycerol Metabolism May Alleviate Hyposalinity Stress

Glycerol’s role in expanding the water activity limit for microbial cell function has been recently discussed in fungi [39] although not so much is known in archaea. It is known that the habitat of *H. salinarum* is also home to the algae *Dunaliella salina* which produces glycerol, a compatible solute, to deal with the osmotic imbalance that occurs due to variations in environmental conditions [40,41]. The co-culture of the two microorganisms showed that *D. salina* secretes substances and nutrients important for the survival of *H. salinarum* [40]. We hypothesized that the up-regulation of genes encoding glycerol-3 phosphate dehydrogenase (*glpA*, *glpB*, and *glpC*) could be related to the availability of glycerol secreted by *D. salina*, allowing an increase in O.D. of cultures under these conditions when supplemented with glycerol. Glycerol metabolism was evaluated by performing growth curves at 2.6 M and 4.3 M NaCl with the addition of glycerol at concentrations compatible with those secreted by *D. salina*: mean of 30 µg/mL and a maximum of 60 µg/mL [26] (Figure 2). OD_600_ low salt stress is always smaller than optimal, but the gap decreases as glycerol supplementation increases, validating a prediction from the glycerol-3-phosphate dehydrogenase up-regulation. Understanding the function of glycerol for this microorganism is crucial since we found that supplementation under low salinity stress improved growth but supplementation under optimal salinity deteriorated the growth (data not shown).

### 4.4. Post-Transcriptional Regulation under Hyposalinity Stress

By choosing experimental conditions matching published proteomics data to perform our high-throughput transcriptome sequencing, we could leverage multi-omics comparisons and search for evidence of post-transcriptional regulation in osmotic stress. The majority of the 165 genes commonly observed (a ~6% sample of the coding genome) indeed follow the canonical expected trend (Figure 3a): 26% increased RNA expression under stress followed by an increase in protein expression and 32% decreased both RNA and protein expression under stress. The remaining (100% − 58% = 42%) were divided between (a) 26%, for which an increase in RNA expression was not met by protein increases (including some sharp decreasing cases), indicative of post-transcriptional processing blocking the canonical message, and (b) 17%, for which protein expression increased under stress despite less RNA available, a harder to interpret scenario.

Recent evidence for environment-specific translation systems with distinct ribosomal protein composition [42] led us to follow up on a case of putative post-transcriptional regulation under low salt stress: *rpl15e*, a 50S ribosomal protein L15e. Relaxing our stringent double-differentially expressed filter by allowing cases with >2-fold increase in RNA levels but with modest/absent protein up-regulation, it is possible to find other ribosomal components with similar Transcript Processing site (TPS) signals, such as *rps19e* or *rpl11*. Ribosome profile footprint combined with TPS evidence (Figure 3b) might indicate that *rpl15e* transcripts have their translation interrupted by ribosome stalling, probably followed by a “No-Go” decay-type cleavage, a scenario that would explain why a >2-fold increase in RNA levels was not accompanied by increased protein levels. A thermodynamically oriented RNA structure prediction method [23] that returns stable options from the ensemble instead of only the minimal energy option, shows that two possible structures exist (Figure 3b and Figure S3a) in a 56:44% equilibrium, which differs markedly exactly at the TPS site. Since it is hard to use prediction tools that consider salt concentrations, we emulate different ionic environments predicting secondary structures by a temperature gradient (10 °C to 77 °C, Figure S4a–i), disregarding the actual numerical data and keeping the qualitative result. Depending on the thermodynamical context, the 56:44% sharing changes (Figure S4 shows the median structure). Since a decrease in salt concentration, emulated here by increasing temperature, would apparently diminish the stability of secondary segments [43], we suggest that *rpl15e* 5’UTR unpairing at higher salt concentrations allows an internal hairpin to form (Figure S4c,d), which, in turn, impair ribosome’s march on. The presence of an SmAP1 chaperone binding signal at 5’ UTR and a faint antisense RNA signal (https://halodata.systemsbiology.net/viewgene/VNG_0177G (accessed on 9 June 2022)) complicate the regulatory scenario, which remains to be further investigated.

We put forward the hypothesis that these osmo-sensitive structures play a role in post-transcriptional condition-specific ribosome composition. 

## 5. Conclusions

In conclusion, although being a basic and well-studied experimental set-up, especially for a halophile microorganism, osmotic stress is still revealing nuances on how the regulatory and adaptative processes play out to ensure survival in such a biochemically harsh environment.

## Data Availability

RNA-seq data are available in the NCBI’s SRA database under accession number SRP290963. Information about the samples and a project overview is available at NCBI under BioProject accession number PRJNA674319.

## Supplementary Materials

The following supporting information can be downloaded at: https://www.mdpi.com/article/10.3390/microorganisms10122442/s1, Figure S1: Growth curve of *H. salinarum* under the conditions of optimum salt (4.3 M of NaCl) and low salt (2.6 M of NaCl). Figure S2: Growth curve of *H. salinarum* pre-inoculum after a 40-h treatment with an optimal or low salt medium. Figure S3: Predicted rainbow diagram of *rpl15e* representing the two thermodynamically stable predictions. Figure S4: Emulation of different ionic environments predicting secondary structures of *rpl15e* by a temperature gradient. Figure S5: Coverage of VNG_0177G (*rpl15e*) region in RNA-seq experiments from different growth phases. Table S1: Results of RNA-seq analysis. File S1: 283 different expression of genes list.

## References

[1] Oren A., Dworkin M., Falkow S., Rosenberg E., Schleifer K.-H., Stackebrandt E. (2006). Life at High Salt Concentrations. The Prokaryotes: Volume 2: Ecophysiology and Biochemistry.

[2] Lee C.J.D., McMullan P.E., O’Kane C.J., Stevenson A., Santos I.C., Roy C., Ghosh W., Mancinelli R.L., Mormile M.R., McMullan G. (2018). NaCl-Saturated Brines Are Thermodynamically Moderate, Rather than Extreme, Microbial Habitats. FEMS Microbiol. Rev..

[3] Stevenson A., Cray J.A., Williams J.P., Santos R., Sahay R., Neuenkirchen N., McClure C.D., Grant I.R., Houghton J.D., Quinn J.P. (2015). Is There a Common Water-Activity Limit for the Three Domains of Life?. ISME J..

[4] Poli A., Finore I., Romano I., Gioiello A., Lama L., Nicolaus B. (2017). Microbial Diversity in Extreme Marine Habitats and Their Biomolecules. Microorganisms.

[5] Vauclare P., Madern D., Girard E., Gabel F., Zaccai G., Franzetti B. (2014). New Insights into Microbial Adaptation to Extreme Saline Environments. BIO Web Conf..

[6] Ng W.V., Kennedy S.P., Mahairas G.G., Berquist B., Pan M., Shukla H.D., Lasky S.R., Baliga N.S., Thorsson V., Sbrogna J. (2000). Genome Sequence of Halobacterium Species NRC-1. Proc. Natl. Acad. Sci. USA.

[7] Van P.T., Schmid A.K., King N.L., Kaur A., Pan M., Whitehead K., Koide T., Facciotti M.T., Goo Y.-A., Deutsch E.W. (2008). Halobacterium Salinarum NRC-1 PeptideAtlas: Strategies for Targeted Proteomics. J. Proteome Res..

[8] Vauclare P., Marty V., Fabiani E., Martinez N., Jasnin M., Gabel F., Peters J., Zaccai G., Franzetti B. (2015). Molecular Adaptation and Salt Stress Response of Halobacterium Salinarum Cells Revealed by Neutron Spectroscopy. Extremophiles.

[9] Leuko S., Raftery M.J., Burns B.P., Walter M.R., Neilan B.A. (2009). Global Protein-Level Responses of Halobacterium Salinarum NRC-1 to Prolonged Changes in External Sodium Chloride Concentrations. J. Proteome Res..

[10] Coker J.A., DasSarma P., Kumar J., Müller J.A., DasSarma S. (2007). Transcriptional Profiling of the Model Archaeon Halobacterium Sp. NRC-1: Responses to Changes in Salinity and Temperature. Saline Syst..

[11] Vauclare P., Natali F., Kleman J.P., Zaccai G., Franzetti B. (2020). Surviving Salt Fluctuations: Stress and Recovery in Halobacterium Salinarum, an Extreme Halophilic Archaeon. Sci. Rep..

[12] Madern D., Ebel C., Zaccai G. (2000). Halophilic Adaptation of Enzymes. Extremophiles.

[13] Lanyi J.K. (1974). Salt-Dependent Properties of Proteins from Extremely Halophilic Bacteria. Bacteriol. Rev..

[14] Kennedy S.P., Ng W.V., Salzberg S.L., Hood L., DasSarma S. (2001). Understanding the Adaptation of Halobacterium Species NRC-1 to Its Extreme Environment through Computational Analysis of Its Genome Sequence. Genome Res..

[15] Sakrikar S., Schmid A. (2021). An Archaeal Histone-like Protein Regulates Gene Expression in Response to Salt Stress. Nucleic Acids Res..

[16] Kish A., Kirkali G., Robinson C., Rosenblatt R., Jaruga P., Dizdaroglu M., DiRuggiero J. (2009). Salt Shield: Intracellular Salts Provide Cellular Protection against Ionizing Radiation in the Halophilic Archaeon, Halobacterium Salinarum NRC-1. Environ. Microbiol..

[17] Kurt-Kızıldoğan A., Abanoz B., Okay S. (2017). Global Transcriptome Analysis of Halolamina Sp. to Decipher the Salt Tolerance in Extremely Halophilic Archaea. Gene.

[18] Mei Y., Liu H., Zhang S., Yang M., Hu C., Zhang J., Shen P., Chen X. (2017). Effects of Salinity on the Cellular Physiological Responses of Natrinema Sp. J7-2. PLoS ONE.

[19] Almeida-Dalmet S., Litchfield C.D., Gillevet P., Baxter B.K. (2018). Differential Gene Expression in Response to Salinity and Temperature in a Haloarcula Strain from Great Salt Lake, Utah. Genes.

[20] Ten-Caten F., Vêncio R.Z., Lorenzetti A.P.R., Zaramela L.S., Santana A.C., Koide T. (2018). Internal RNAs Overlapping Coding Sequences Can Drive the Production of Alternative Proteins in Archaea. RNA Biol..

[21] Vêncio R.Z.N., Koide T. (2005). HTself: Self-Self Based Statistical Test for Low Replication Microarray Studies. DNA Res..

[22] Thomas P.D., Ebert D., Muruganujan A., Mushayahama T., Albou L.-P., Mi H. (2022). PANTHER: Making Genome-Scale Phylogenetics Accessible to All. Protein Sci..

[23] Aalberts D.P., Jannen W.K. (2013). Visualizing RNA Base-Pairing Probabilities with RNAbow Diagrams. RNA.

[24] Lorenz R., Bernhart S.H., Höner Zu Siederdissen C., Tafer H., Flamm C., Stadler P.F., Hofacker I.L. (2011). ViennaRNA Package 2.0. Algorithms Mol. Biol..

[25] Ibrahim A.G.A.E.-R., Vêncio R.Z.N., Lorenzetti A.P.R., Koide T. (2021). Halobacterium Salinarum and Haloferax Volcanii Comparative Transcriptomics Reveals Conserved Transcriptional Processing Sites. Genes.

[26] Chen H., Jiang J.G., Wu G.H. (2009). Effects of Salinity Changes on the Growth of Dunaliella Salina and Its Isozyme Activities of Glycerol-3-Phosphate Dehydrogenase. J. Agric. Food Chem..

[27] Zaretsky M., Darnell C.L., Schmid A.K., Eichler J. (2019). N-Glycosylation Is Important for Halobacterium Salinarum Archaellin Expression, Archaellum Assembly and Cell Motility. Front. Microbiol..

[28] Collins M., Afolayan S., Igiraneza A.B., Schiller H., Krespan E., Beiting D.P., Dyall-Smith M., Pfeiffer F., Pohlschroder M. (2020). Mutations Affecting HVO_1357 or HVO_2248 Cause Hypermotility in Haloferax Volcanii, Suggesting Roles in Motility Regulation. Genes.

[29] de Almeida J.P.P., Vêncio R.Z.N., Lorenzetti A.P.R., Ten-Caten F., Gomes-Filho J.V., Koide T. (2019). The Primary Antisense Transcriptome of Halobacterium Salinarum NRC-1. Genes.

[30] Lorenzetti A.P.R., Kusebauch U., Zaramela L.S., Wu W.-J., de Almeida J.P.P., Turkarslan S., de Lomana A.L.G., Gomes-Filho J.V., Vêncio R.Z.N., Moritz R.L. (2022). A Genome-Scale Atlas Reveals Complex Interplay of Transcription and Translation in an Archaeon. bioRxiv.

[31] Pfeifer F. (2015). Haloarchaea and the Formation of Gas Vesicles. Life.

[32] Albers S.-V., Jarrell K.F. (2015). The Archaellum: How Archaea Swim. Front. Microbiol..

[33] Walsby A.E. (1994). Gas Vesicles. Microbiol. Rev..

[34] Pfeifer F. (2022). Recent Advances in the Study of Gas Vesicle Proteins and Application of Gas Vesicles in Biomedical Research. Life.

[35] Albers S.-V., Jarrell K.F. (2018). The Archaellum: An Update on the Unique Archaeal Motility Structure. Trends Microbiol..

[36] Kinosita Y., Nishizaka T. (2018). Cross-Kymography Analysis to Simultaneously Quantify the Function and Morphology of the Archaellum. Biophys. Phys..

[37] Sasaki J., Spudich J.L. (2008). Signal Transfer in Haloarchaeal Sensory Rhodopsin–Transducer Complexes. Photochem. Photobiol..

[38] Yaakop A.S., Chan K.-G., Ee R., Lim Y.L., Lee S.-K., Manan F.A., Goh K.M. (2016). Characterization of the Mechanism of Prolonged Adaptation to Osmotic Stress of Jeotgalibacillus Malaysiensis via Genome and Transcriptome Sequencing Analyses. Sci. Rep..

[39] Stevenson A., Hamill P.G., Medina Á., Kminek G., Rummel J.D., Dijksterhuis J., Timson D.J., Magan N., Leong S.-L.L., Hallsworth J.E. (2017). Glycerol Enhances Fungal Germination at the Water-Activity Limit for Life. Environ. Microbiol..

[40] Orellana M.V., Pang W.L., Durand P.M., Whitehead K., Baliga N.S. (2013). A Role for Programmed Cell Death in the Microbial Loop. PLoS ONE.

[41] Williams T.J., Allen M., Tschitschko B., Cavicchioli R. (2017). Glycerol Metabolism of Haloarchaea. Environ. Microbiol..

[42] López García de Lomana A., Kusebauch U., Raman A.V., Pan M., Turkarslan S., Lorenzetti A.P.R., Moritz R.L., Baliga N.S. (2020). Selective Translation of Low Abundance and Upregulated Transcripts in *Halobacterium Salinarum*. mSystems.

[43] Wang Y., Liu T., Yu T., Tan Z.-J., Zhang W. (2020). Salt Effect on Thermodynamics and Kinetics of a Single RNA Base Pair. RNA.

