# Supplementary material for: Low Salt Influences Archaellum-Based Motility, Glycerol Metabolism, and Gas Vesicles Biogenesis in Halobacterium salinarum
Source: Microorganisms. 2022 Dec 10;10(12):2442. doi: 10.3390/microorganisms10122442 (PMC9786353; doi:10.3390/microorganisms10122442)
Supplement: Supplementary file 1 [file microorganisms-10-02442-s001.zip › microorganisms-2047039-Figures.pdf]

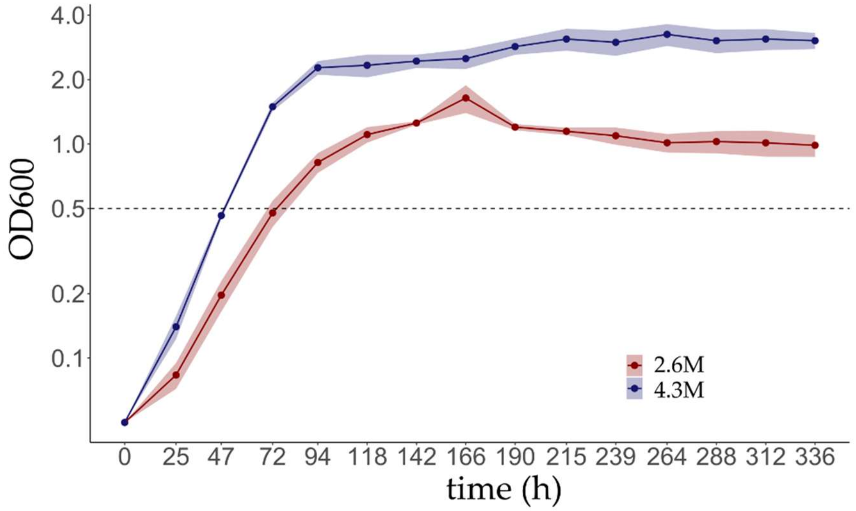

Figure S1. Growth curve of *H. salinarum* under the conditions of optimum salt (4.3 M of NaCl) and low salt (2.6 M of NaCl).

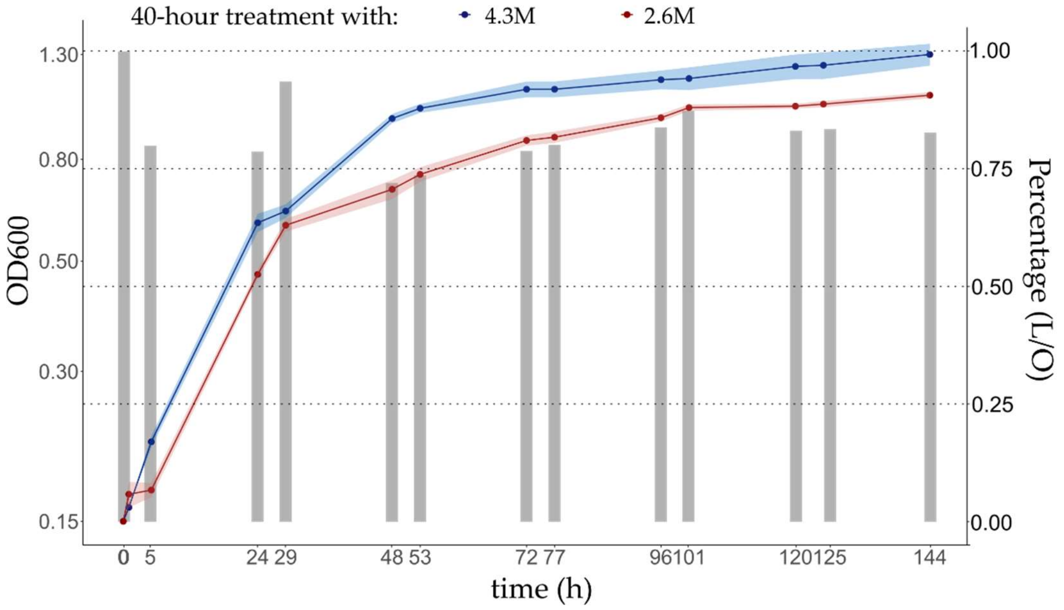

Figure S2. ***H. salinarum* pre-inoculum after a 40-hour treatment with optimal or low salt medium.** The right Y-axis represents the relative percentage of cells recovered from the low salt treatment.

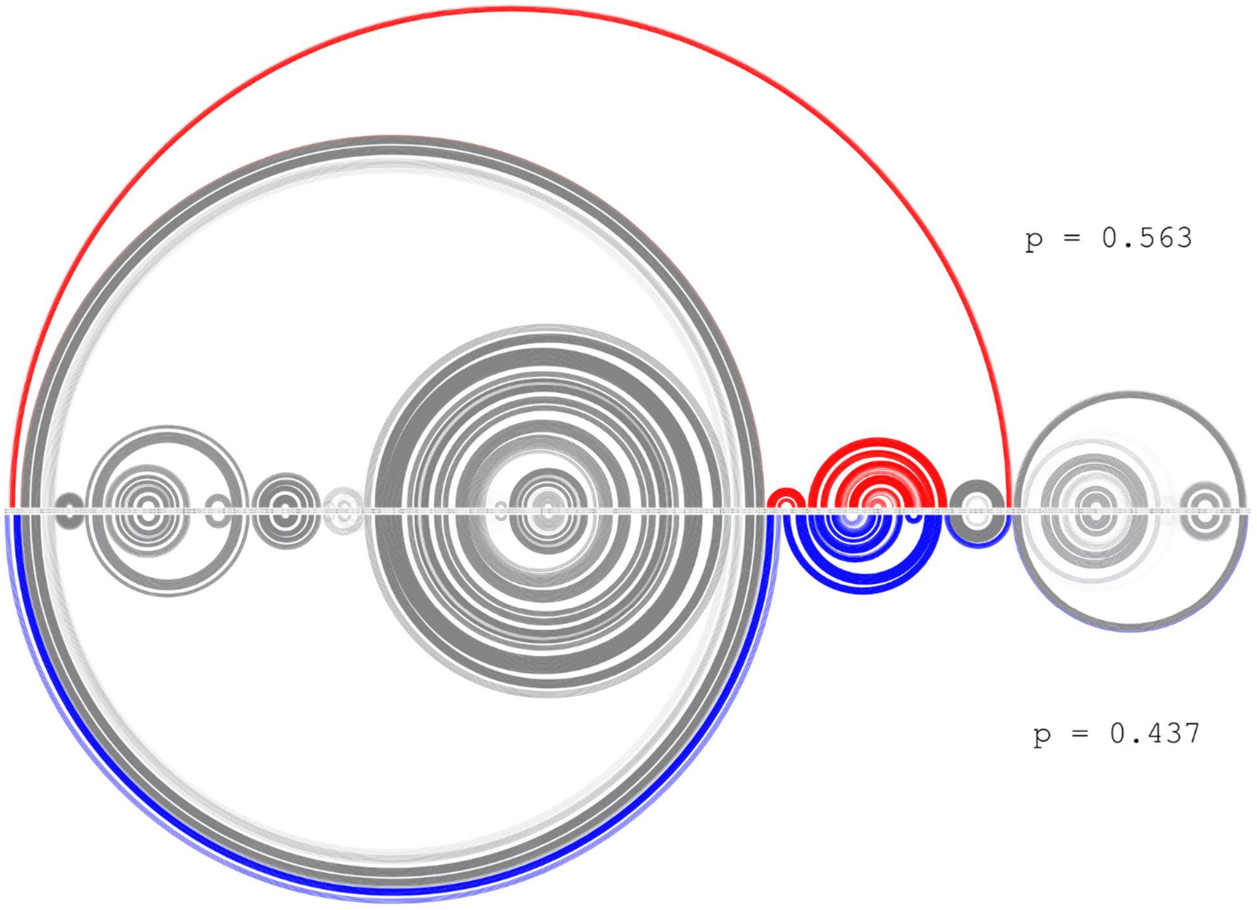

Figure S3. Predicted rainbow diagram of *rpl15e* representing the two thermodynamically stable predictions.

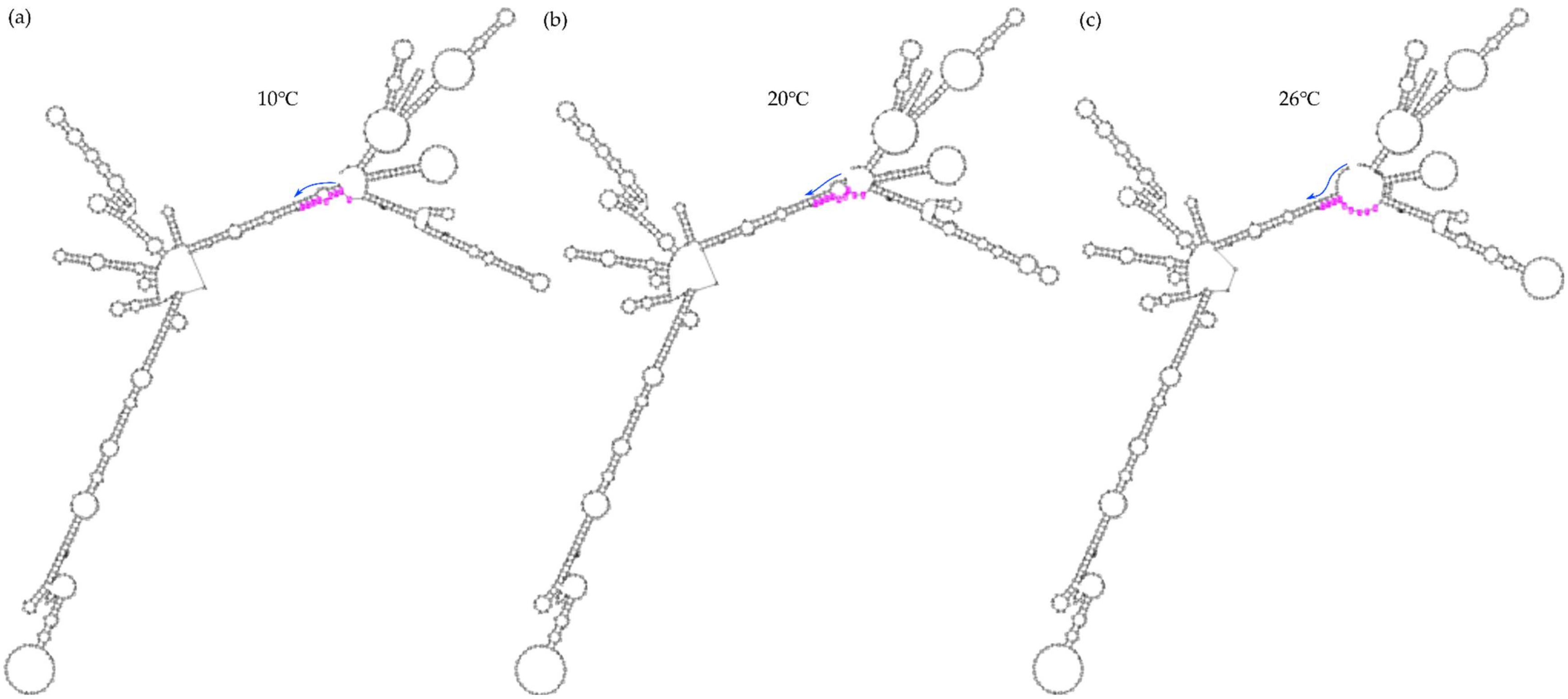

Figure S4. Emulation of different ionic environments predicting secondary structures of *rpl15e* by a temperature gradient. Blue arrow represents the beginning of the transcript and magenta nucleotides represent TPS upstream sequence. (**a**) Structure at 10°C (**b**) 20°C (**c**) 26°C (**d**) 28°C (**e**) 30°C (**f**) 36°C (**g**)46°C (**h**) 60°C (**i**) 77°C.

(d) (e) (f)

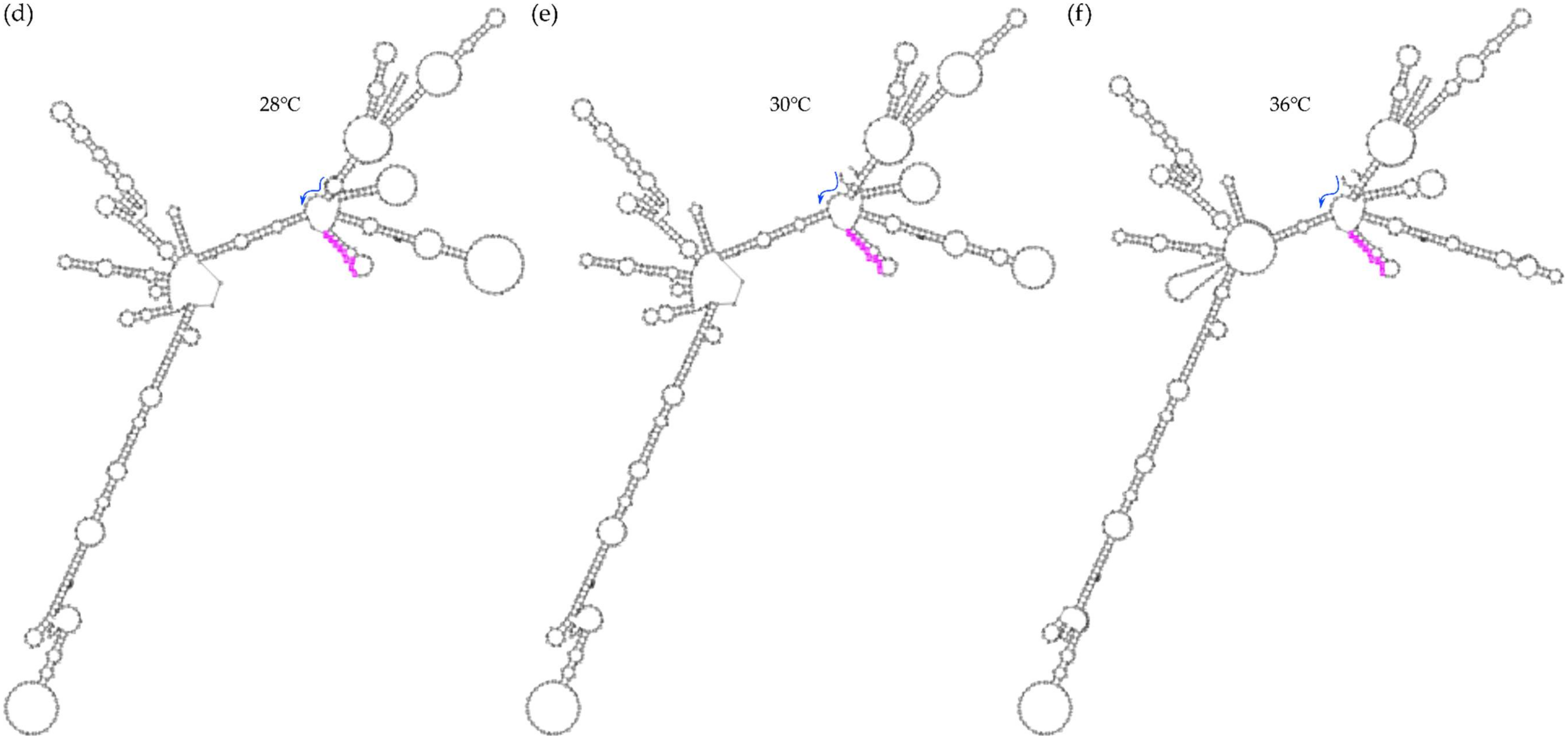

Figure S4. Emulation of different ionic environments predicting secondary structures of *rpl15e* by a temperature gradient. Blue arrow represents the beginning of the transcript and magenta nucleotides represent TPS upstream sequence. (**a**) Structure at 10°C (**b**) 20°C (**c**) 26°C (**d**) 28°C (**e**) 30°C (**f**) 36°C (**g**)46°C (**h**) 60°C (**i**) 77°C.

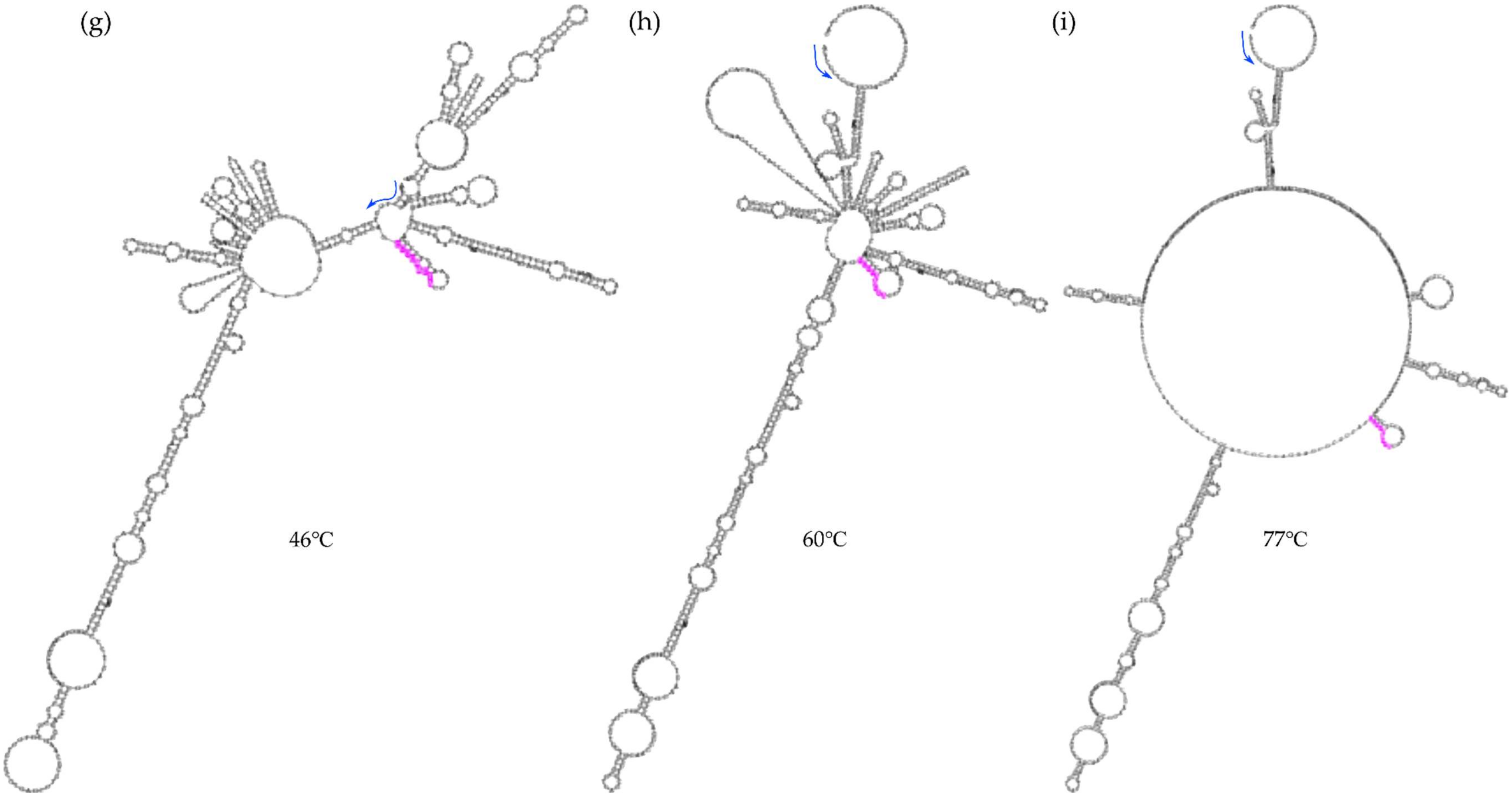

Figure S4. Emulation of different ionic environments predicting secondary structures of *rpl15e* by a temperature gradient. Blue arrow represents the beginning of the transcript and magenta nucleotides represent TPS upstream sequence. (**a**) Structure at 10°C (**b**) 20°C (**c**) 26°C (**d**) 28°C (**e**) 30°C (**f**) 36°C (**g**)46°C (**h**) 60°C (**i**) 77°C.

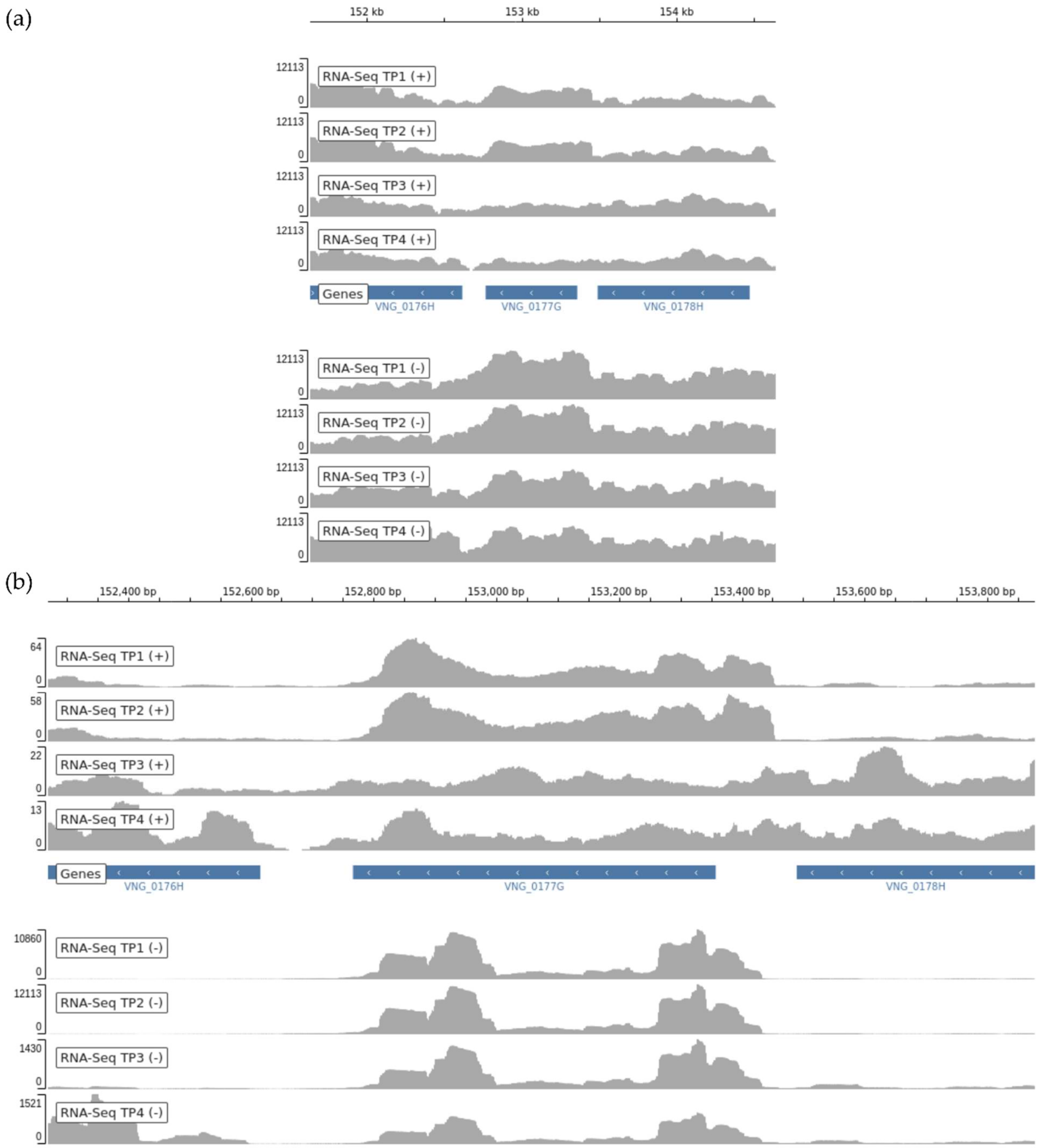

Figure S5. Coverage of VNG_0177G (*rpl15e*) region in RNA-seq experiments from different growth phases. TP1: early exponential growth phase; TP2: mid-exponential growth phase; TP4: stationary phase. (**a**) Fixed data range (**b**) Data range relative to each track.
