# Supplementary material for: Low Salt Influences Archaellum-Based Motility, Glycerol Metabolism, and Gas Vesicles Biogenesis in Halobacterium salinarum
Source: Microorganisms. 2022 Dec 10;10(12):2442. doi: 10.3390/microorganisms10122442 (PMC9786353; doi:10.3390/microorganisms10122442)
Supplement: Supplementary file 1 [file microorganisms-10-02442-s001.zip › microorganisms-2047039-File S1.zip!/list-283-diff-express-genes-PANTHER-category-v01.html]

this frame prevents back forward cache


The mission of the PANTHER knowledgebase is to support biomedical and other research by providing **comprehensive information about the evolution of protein-coding gene families**, particularly protein phylogeny, function and genetic variation impacting that function. More...

|  |  |  |
| --- | --- | --- |
|  | All Genes and orthologs Pathways PANTHER families Ontology terms | Go |

|  |  |  |  |  |  |  |  |  |  |  |  |  |  |  |  |  |  |
| --- | --- | --- | --- | --- | --- | --- | --- | --- | --- | --- | --- | --- | --- | --- | --- | --- | --- |
|  | Home |  | About |  | Data Version |  | Tools |  | API/Services |  | Publications |  | Workspace |  | Downloads |  | FAQ/Help/Tutorial |

|  |  |  |  |  |  |
| --- | --- | --- | --- | --- | --- |
|  | Login |  | Register |  | Contact us |

|  |  |  |
| --- | --- | --- |
|  | **PANTHER17.0 Released.** | Current Release: **PANTHER 17.0**  \|  **15,619** family phylogenetic trees  \|  **143** species  \|  News   Whole genome function views |


PANTHER Gene list


  Customize Gene list
  Click to view Enhancer Data

|  |  |  |
| --- | --- | --- |
| Convert List to:  -Select-  PANTHER Ontology Terms PANTHER Families Pathways Pathway Components | Send list to:  -Select- Workspace File Text |  |

|  |  |  |
| --- | --- | --- |
| Display:  1020305010020050010001000020000 items per page |  | Refine Search |

|  |  |  |
| --- | --- | --- |
| Hits 1-237 of 237 | [ page: (1) ] | Number of mapped ids found 237 IDs not found (46) |

|  |  |  |  |  |  |  |  |
| --- | --- | --- | --- | --- | --- | --- | --- |
| clr all | | Gene ID | **Mapped IDs** | Gene Name Gene Symbol Persistent id Orthologs | PANTHER Family/Subfamily | PANTHER Protein Class | Species |
|  | **1.** | HALSA\|EnsemblGenome=VNG\_2473G\|UniProtKB=Q9HMM4 | VNG\_2473G | DNA repair and recombination protein RadA radA PTN001118391 orthologs | MEIOTIC RECOMBINATION PROTEIN DMC1/LIM15 HOMOLOG (PTHR22942:SF30) | DNA metabolism protein | Halobacterium salinarum |
|  | **2.** | HALSA\|EnsemblGenome=VNG\_6362G\|UniProtKB=Q9HHJ8 | VNG\_6362G | DNA-directed DNA polymerase polB2 PTN004148471 orthologs | DNA POLYMERASE DELTA CATALYTIC SUBUNIT (PTHR10322:SF23) | DNA metabolism protein | Halobacterium salinarum |
|  | **3.** | HALSA\|EnsemblGenome=VNG\_1754G\|UniProtKB=Q9HP89 | VNG\_1754G | Deoxyribodipyrimidine photo-lyase phr1 PTN000894446 orthologs | (6-4)-PHOTOLYASE, ISOFORM A (PTHR11455:SF9) | DNA photolyase | Halobacterium salinarum |
|  | **4.** | HALSA\|EnsemblGenome=VNG\_1224G\|UniProtKB=Q9HQC7 | VNG\_1224G | ORC1-type DNA replication protein 8 orc8 PTN000844883 orthologs | ORC1-TYPE DNA REPLICATION PROTEIN (PTHR10763:SF22) | replication origin binding protein | Halobacterium salinarum |
|  | **5.** | HALSA\|EnsemblGenome=VNG\_2475C\|UniProtKB=Q9HMM2 | VNG\_2475C | tRNA(Phe) (4-demethylwyosine(37)-C(7)) aminocarboxypropyltransferase taw2 PTN001146796 orthologs | TRNA(PHE) (4-DEMETHYLWYOSINE(37)-C(7)) AMINOCARBOXYPROPYLTRANSFERASE (PTHR23245:SF41) | RNA methyltransferase | Halobacterium salinarum |
|  | **6.** | HALSA\|EnsemblGenome=VNG\_1980C\|UniProtKB=Q9HNR6 | VNG\_1980C | tRNA (pseudouridine(54)-N(1))-methyltransferase trmY PTN002221506 orthologs | TRNA (PSEUDOURIDINE(54)-N(1))-METHYLTRANSFERASE (PTHR40703:SF1) | RNA processing factor | Halobacterium salinarum |
|  | **7.** | HALSA\|EnsemblGenome=VNG\_0869G\|UniProtKB=Q9HR45 | VNG\_0869G | Transcription initiation factor IIB 4 tfbD PTN000907515 orthologs | TRANSCRIPTION INITIATION FACTOR IIB (PTHR11618:SF13) | general transcription factor | Halobacterium salinarum |
|  | **8.** | HALSA\|EnsemblGenome=VNG\_0734G\|UniProtKB=Q9HRE6 | VNG\_0734G | Transcription initiation factor IIB 2 tfbB PTN000907513 orthologs | TRANSCRIPTION INITIATION FACTOR IIB (PTHR11618:SF13) | general transcription factor | Halobacterium salinarum |
|  | **9.** | HALSA\|EnsemblGenome=AAC82884\|UniProtKB=O52004 | VNG\_6140G | TATA-box-binding protein C tbpC2 PTN002227227 orthologs | RT01008P-RELATED (PTHR10126:SF42) | general transcription factor | Halobacterium salinarum |
|  | **10.** | HALSA\|EnsemblGenome=VNG\_0832C\|UniProtKB=Q9HR71 | VNG\_0832C | Putative dimethyl sulfoxide reductase chaperone dmsD PTN002151535 orthologs | TAT PROOFREADING CHAPERONE DMSD-RELATED (PTHR34227:SF1) | chaperone | Halobacterium salinarum |
|  | **11.** | HALSA\|EnsemblGenome=VNG\_6201G\|UniProtKB=Q9HHW1 | VNG\_6201G | Heat shock protease protein hsp5 PTN008519712 orthologs | HEAT SHOCK PROTEIN 42 (PTHR11527:SF175) | chaperone | Halobacterium salinarum |
|  | **12.** | HALSA\|EnsemblGenome=VNG\_0451G\|UniProtKB=Q9HS14 | VNG\_0451G | Phosphate-specific transport system accessory protein PhoU phoU PTN000797088 orthologs | PHOSPHATE-SPECIFIC TRANSPORT SYSTEM ACCESSORY PROTEIN PHOU (PTHR42930:SF3) | protein-binding activity modulator | Halobacterium salinarum |
|  | **13.** | HALSA\|EnsemblGenome=VNG\_0458G\|UniProtKB=Q9HS09 | VNG\_0458G | PhoU domain protein prp1 PTN000797080 orthologs | PHOSPHATE REGULATORY PROTEIN-LIKE PROTEIN (PTHR42930:SF5) | protein-binding activity modulator | Halobacterium salinarum |
|  | **14.** | HALSA\|EnsemblGenome=VNG\_2587C\|UniProtKB=Q9HME0 | VNG\_2587C | Probable GTP-binding protein EngB engB PTN000909753 orthologs | ENGB-TYPE G DOMAIN-CONTAINING PROTEIN (PTHR11649:SF13) | G-protein | Halobacterium salinarum |
|  | **15.** | HALSA\|EnsemblGenome=VNG\_1234C\|UniProtKB=Q9HQB9 | VNG\_1234C | Sensor box histidine kinase VNG\_1234C PTN001976592 orthologs | OSMOSENSING HISTIDINE PROTEIN KINASE SLN1 (PTHR43047:SF5) | histidine kinase receptor of two-component system | Halobacterium salinarum |
|  | **16.** | HALSA\|EnsemblGenome=VNG\_0916G\|UniProtKB=Q9HR10 | VNG\_0916G | Adaptive-response sensory-kinase ark PTN001232774 orthologs | HISTIDINE KINASE 1 (PTHR43711:SF1) | histidine kinase receptor of two-component system | Halobacterium salinarum |
|  | **17.** | HALSA\|EnsemblGenome=VNG\_1009G\|UniProtKB=P13075 | VNG\_1009G | Flagellin A2 flaA2 PTN002185813 orthologs | FLAGELLIN B1 (PTHR35903:SF1) | structural protein | Halobacterium salinarum |
|  | **18.** | HALSA\|EnsemblGenome=VNG\_0962G\|UniProtKB=P13078 | VNG\_0962G | Flagellin B3 flaB3 PTN002185812 orthologs | FLAGELLIN B1 (PTHR35903:SF1) | structural protein | Halobacterium salinarum |
|  | **19.** | HALSA\|EnsemblGenome=VNG\_0961G\|UniProtKB=P13077 | VNG\_0961G | Flagellin B2 flaB2 PTN002185814 orthologs | FLAGELLIN B1 (PTHR35903:SF1) | structural protein | Halobacterium salinarum |
|  | **20.** | HALSA\|EnsemblGenome=VNG\_0960G\|UniProtKB=P61116 | VNG\_0960G | Flagellin B1 flaB1 PTN002185811 orthologs | FLAGELLIN B1 (PTHR35903:SF1) | structural protein | Halobacterium salinarum |
|  | **21.** | HALSA\|EnsemblGenome=VNG\_1008G\|UniProtKB=P61118 | VNG\_1008G | Flagellin A1 flaA1a PTN002185815 orthologs | FLAGELLIN B1 (PTHR35903:SF1) | structural protein | Halobacterium salinarum |
|  | **22.** | HALSA\|EnsemblGenome=VNG\_0455G\|UniProtKB=Q9HS11 | VNG\_0455G | Phosphate transport system permease protein pstC2 PTN001252325 orthologs | PHOSPHATE TRANSPORT SYSTEM PERMEASE-RELATED (PTHR42727:SF1) | transporter | Halobacterium salinarum |
|  | **23.** | HALSA\|EnsemblGenome=VNG\_1240G\|UniProtKB=Q9HQB3 | VNG\_1240G | Amino acid transporter yhdG PTN000926866 orthologs | SOBREMESA, ISOFORM B (PTHR11785:SF512) | transporter | Halobacterium salinarum |
|  | **24.** | HALSA\|EnsemblGenome=VNG\_2361G\|UniProtKB=Q9HMW2 | VNG\_2361G | ABC-type transport system permease protein(Probable substrate dipeptide/oligopeptide) appC PTN001252814 orthologs | OPPC IN A BINDING PROTEIN-DEPENDENT TRANSPORT SYSTEM (PTHR43839:SF1) | transporter | Halobacterium salinarum |
|  | **25.** | HALSA\|EnsemblGenome=VNG\_0321G\|UniProtKB=Q9HSA8 | VNG\_0321G | Bifunctional short chain isoprenyl diphosphate synthase ids PTN000814100 orthologs | STOMATIN-LIKE PROTEIN 2, MITOCHONDRIAL (PTHR43327:SF10) | transporter | Halobacterium salinarum |
|  | **26.** | HALSA\|EnsemblGenome=VNG\_1350C\|UniProtKB=Q9HQ34 | VNG\_1350C | DMT superfamily transport protein VNG\_1350C PTN001284413 orthologs | EAMA DOMAIN-CONTAINING PROTEIN (PTHR32322:SF2) | transporter | Halobacterium salinarum |
|  | **27.** | HALSA\|EnsemblGenome=VNG\_0453G\|UniProtKB=Q9HS12 | VNG\_0453G | Phosphate transport system permease protein PstA pstA2 PTN001252341 orthologs | PHOSPHATE TRANSPORT SYSTEM PERMEASE PROTEIN PSTA (PTHR43470:SF5) | transporter | Halobacterium salinarum |
|  | **28.** | HALSA\|EnsemblGenome=VNG\_1462G\|UniProtKB=Q9HPV0 | VNG\_1462G | Bacterioopsin-associated chaperone cdc48a PTN001130627 orthologs | NUCLEAR VALOSIN-CONTAINING PROTEIN-LIKE (PTHR23077:SF171) | primary active transporter | Halobacterium salinarum |
|  | **29.** | HALSA\|EnsemblGenome=VNG\_2343G\|UniProtKB=Q9HMX3 | VNG\_2343G | ABC-type transport system ATP-binding protein(Probable substrate dipeptide/oligopeptide) ykfD PTN001199171 orthologs | D,D-DIPEPTIDE TRANSPORT ATP-BINDING PROTEIN DDPF-RELATED (PTHR43776:SF7) | ATP-binding cassette (ABC) transporter | Halobacterium salinarum |
|  | **30.** | HALSA\|EnsemblGenome=VNG\_0668C\|UniProtKB=Q9HRJ6 | VNG\_0668C | ABC-type transport system ATP-binding protein VNG\_0668C PTN001199392 orthologs | ANTIBIOTIC RESISTANCE ABC TRANSPORTER ATP-BINDING PROTEIN (PTHR43582:SF4) | ATP-binding cassette (ABC) transporter | Halobacterium salinarum |
|  | **31.** | HALSA\|EnsemblGenome=VNG\_0452G\|UniProtKB=Q9HS13 | VNG\_0452G | Phosphate import ATP-binding protein PstB 1 pstB1 PTN001198609 orthologs | ABC TRANSPORTER I FAMILY MEMBER 17 (PTHR43423:SF1) | ATP-binding cassette (ABC) transporter | Halobacterium salinarum |
|  | **32.** | HALSA\|EnsemblGenome=VNG\_2363G\|UniProtKB=Q9HMW1 | VNG\_2363G | Oligopeptide ABC transporter ATP-binding oppD1 PTN001198822 orthologs | OLIGOPEPTIDE TRANSPORT ATP-BINDING PROTEIN APPD (PTHR43297:SF2) | ATP-binding cassette (ABC) transporter | Halobacterium salinarum |
|  | **33.** | HALSA\|EnsemblGenome=VNG\_2636G\|UniProtKB=Q9HMA2 | VNG\_2636G | Excision nuclease chain A uvrA PTN001284007 orthologs | UVRABC SYSTEM PROTEIN A (PTHR43152:SF3) | ATP-binding cassette (ABC) transporter | Halobacterium salinarum |
|  | **34.** | HALSA\|EnsemblGenome=VNG\_2349G\|UniProtKB=Q9HMW9 | VNG\_2349G | Dipeptide ABC transporter dipeptide-binding dppA PTN001575934 orthologs | D,D-DIPEPTIDE-BINDING PERIPLASMIC PROTEIN DDPA-RELATED (PTHR30290:SF9) | ATP-binding cassette (ABC) transporter | Halobacterium salinarum |
|  | **35.** | HALSA\|EnsemblGenome=VNG\_2344G\|UniProtKB=Q9HMX2 | VNG\_2344G | ABC-type transport system ATP-binding protein(Probable substrate dipeptide/oligopeptide) oppD2 PTN001198823 orthologs | OLIGOPEPTIDE TRANSPORT ATP-BINDING PROTEIN APPD (PTHR43297:SF2) | ATP-binding cassette (ABC) transporter | Halobacterium salinarum |
|  | **36.** | HALSA\|EnsemblGenome=VNG\_2358G\|UniProtKB=Q9HMW4 | VNG\_2358G | ABC-type transport system periplasmic substrate-binding protein (Probable substratedipeptide/oligopeptide) appA PTN001250539 orthologs | D,D-DIPEPTIDE-BINDING PERIPLASMIC PROTEIN DDPA-RELATED (PTHR30290:SF9) | ATP-binding cassette (ABC) transporter | Halobacterium salinarum |
|  | **37.** | HALSA\|EnsemblGenome=VNG\_2359G\|UniProtKB=Q9HMW3 | VNG\_2359G | ABC-type transport system permease protein(Probable substrate dipeptide/oligopeptide) appB PTN001252690 orthologs | ABC TRANSPORTER-RELATED (PTHR30465:SF96) | ATP-binding cassette (ABC) transporter | Halobacterium salinarum |
|  | **38.** | HALSA\|EnsemblGenome=VNG\_0457G\|UniProtKB=Q9HS10 | VNG\_0457G | ABC-type transport system periplasmicsubstrate-binding protein (Probable substrate phosphate) phoX PTN001577340 orthologs | PHOSPHATE-BINDING PROTEIN PSTS (PTHR30570:SF1) | ATP-binding cassette (ABC) transporter | Halobacterium salinarum |
|  | **39.** | HALSA\|EnsemblGenome=VNG\_2365G\|UniProtKB=Q9HMW0 | VNG\_2365G | ABC-type transport system ATP-binding protein(Probable substrate dipeptide/oligopeptide) appF PTN001199172 orthologs | D,D-DIPEPTIDE TRANSPORT ATP-BINDING PROTEIN DDPF-RELATED (PTHR43776:SF7) | ATP-binding cassette (ABC) transporter | Halobacterium salinarum |
|  | **40.** | HALSA\|EnsemblGenome=VNG\_6264G\|UniProtKB=Q9HHR7 | VNG\_6264G | ABC transporter, ATP-binding protein zurA PTN001198669 orthologs | IRON TRANSPORT SYSTEM ATP-BINDING PROTEIN HI\_0361-RELATED (PTHR42734:SF5) | ATP-binding cassette (ABC) transporter | Halobacterium salinarum |
|  | **41.** | HALSA\|EnsemblGenome=VNG\_1544G\|UniProtKB=Q9HPN8 | VNG\_1544G | Chloride channel clc PTN000914197 orthologs | CHLORIDE CHANNEL PROTEIN CLC-E (PTHR43427:SF6) | ion channel | Halobacterium salinarum |
|  | **42.** | HALSA\|EnsemblGenome=VNG\_6112H\|UniProtKB=O51984 | VNG\_6112H | Vng6112h VNG\_6112H PTN002825107 orthologs | INTEGRASE/RECOMBINASE PROTEIN MJ0367-RELATED (PTHR30349:SF79) | viral or transposable element protein | Halobacterium salinarum |
|  | **43.** | HALSA\|EnsemblGenome=VNG\_6076H\|UniProtKB=O54590 | VNG\_6450H | Vng6076h VNG\_6076H PTN002017937 orthologs | INTEGRASE/RECOMBINASE PROTEIN MJ0367-RELATED (PTHR30349:SF79) | viral or transposable element protein | Halobacterium salinarum |
|  | **44.** | HALSA\|EnsemblGenome=VNG\_0437C\|UniProtKB=Q9HS25 | VNG\_0437C | M20 family amidohydrolase (Homolog toindole-3-acetyl-aspartate hydrolase) VNG\_0437C PTN001285815 orthologs | M20\_DIMER DOMAIN-CONTAINING PROTEIN (PTHR30575:SF3) | metalloprotease | Halobacterium salinarum |
|  | **45.** | HALSA\|EnsemblGenome=VNG\_6123G\|UniProtKB=O51991 | VNG\_6123G | ParA domain protein sojD PTN001786465 orthologs | PARA FAMILY PROTEIN CT\_582 (PTHR13696:SF52) | hydrolase | Halobacterium salinarum |
|  | **46.** | HALSA\|EnsemblGenome=VNG\_1769C\|UniProtKB=Q9HP77 | VNG\_1769C | Uncharacterized protein VNG\_1769C PTN002439800 orthologs | 3-DEHYDRO-SCYLLO-INOSOSE HYDROLASE (PTHR35005:SF1) | hydrolase | Halobacterium salinarum |
|  | **47.** | HALSA\|EnsemblGenome=VNG\_0231C\|UniProtKB=Q9HSH2 | VNG\_0231C | Lactamase\_B domain-containing protein VNG\_0231C PTN000940164 orthologs | METALLO-BETA-LACTAMASE DOMAIN-CONTAINING PROTEIN 2 (PTHR42951:SF4) | hydrolase | Halobacterium salinarum |
|  | **48.** | HALSA\|EnsemblGenome=VNG\_1407C\|UniProtKB=Q9HPZ1 | VNG\_1407C | Thioesterase domain protein VNG\_1407C PTN000965050 orthologs | NOVEL THIOESTERASE SUPERFAMILY DOMAIN AND SAPOSIN A-TYPE DOMAIN CONTAINING PROTEIN (0610012H03RIK) (PTHR31793:SF27) | esterase | Halobacterium salinarum |
|  | **49.** | HALSA\|EnsemblGenome=VNG\_2186G\|UniProtKB=Q9HNA0 | VNG\_2186G | Histidine triad family protein (Homolog tobis(5'-nucleosyl)-tetraphosphatase) hit1 PTN001132913 orthologs | HISTIDINE TRIAD NUCLEOTIDE-BINDING PROTEIN 1 (PTHR23089:SF33) | nucleotide phosphatase | Halobacterium salinarum |
|  | **50.** | HALSA\|EnsemblGenome=VNG\_1450G\|UniProtKB=Q9HPW1 | VNG\_1450G | 2',3'-cyclic-nucleotide 2'-phosphodiesterase yfkN PTN000903166 orthologs | 5'-NUCLEOTIDASE (PTHR11575:SF24) | phosphodiesterase | Halobacterium salinarum |
|  | **51.** | HALSA\|EnsemblGenome=VNG\_0653G\|UniProtKB=Q9HRK7 | VNG\_0653G | Methylmalonyl-CoA mutase, subunit alpha mcmA1 PTN002883247 orthologs | METHYLMALONYL-COA MUTASE, LARGE SUBUNIT (PTHR48101:SF1) | mutase | Halobacterium salinarum |
|  | **52.** | HALSA\|EnsemblGenome=VNG\_1567G\|UniProtKB=Q9HPM1 | VNG\_1567G | Cobalt-precorrin-8 methylmutase cbiC PTN001286322 orthologs | COBALT-PRECORRIN-8 METHYLMUTASE (PTHR43588:SF1) | mutase | Halobacterium salinarum |
|  | **53.** | HALSA\|EnsemblGenome=VNG\_1305G\|UniProtKB=Q9HQ67 | VNG\_1305G | Phosphoribosylamine--glycine ligase purD PTN002460116 orthologs | PHOSPHORIBOSYLAMINE--GLYCINE LIGASE, CHLOROPLASTIC (PTHR43472:SF1) | ligase | Halobacterium salinarum |
|  | **54.** | HALSA\|EnsemblGenome=VNG\_0997G\|UniProtKB=Q9HQU8 | VNG\_0997G | Acetyl-CoA synthetase acs2 PTN001185838 orthologs | ACETYL-COENZYME A SYNTHETASE 1 (PTHR24095:SF14) | ligase | Halobacterium salinarum |
|  | **55.** | HALSA\|EnsemblGenome=VNG\_6303G\|UniProtKB=Q9HHN8 | VNG\_6303G | 7-cyano-7-deazaguanine synthase queC PTN001697088 orthologs | 7-CYANO-7-DEAZAGUANINE SYNTHASE (PTHR42914:SF1) | ligase | Halobacterium salinarum |
|  | **56.** | HALSA\|EnsemblGenome=VNG\_1814G\|UniProtKB=Q9HP43 | VNG\_1814G | Carbamoyl-phosphate synthase large chain carB PTN000892197 orthologs | CAD PROTEIN (PTHR11405:SF5) | ligase | Halobacterium salinarum |
|  | **57.** | HALSA\|EnsemblGenome=VNG\_1565G\|UniProtKB=Q9HPM3 | VNG\_1565G | ATP-dependent cobaltochelatase subunit ChlID hmcA PTN002078327 orthologs | MAGNESIUM-CHELATASE SUBUNIT CHLI-2, CHLOROPLASTIC (PTHR32039:SF9) | ligase | Halobacterium salinarum |
|  | **58.** | HALSA\|EnsemblGenome=VNG\_1815G\|UniProtKB=Q9HP42 | VNG\_1815G | Carbamoyl-phosphate synthase small chain carA PTN000892044 orthologs | CARBAMOYL-PHOSPHATE SYNTHASE ARGININE-SPECIFIC SMALL CHAIN (PTHR11405:SF4) | ligase | Halobacterium salinarum |
|  | **59.** | HALSA\|EnsemblGenome=VNG\_0623G\|UniProtKB=Q9HRN1 | VNG\_0623G | Propionyl-CoA carboxylase homolog yngE PTN001110625 orthologs | METHYLCROTONOYL-COA CARBOXYLASE BETA CHAIN, MITOCHONDRIAL (PTHR22855:SF13) | ligase | Halobacterium salinarum |
|  | **60.** | HALSA\|EnsemblGenome=VNG\_1939G\|UniProtKB=Q9HNU7 | VNG\_1939G | Putative phosphoribosylaminoimidazole-succinocarboxamide synthase purC PTN000906420 orthologs | PHOSPHORIBOSYLAMINOIMIDAZOLE-SUCCINOCARBOXAMIDE SYNTHASE (PTHR43700:SF1) | ligase | Halobacterium salinarum |
|  | **61.** | HALSA\|EnsemblGenome=VNG\_0627G\|UniProtKB=Q9HRM8 | VNG\_0627G | Citrate (Pro-3S)-lyase citE PTN001284312 orthologs | HPCH\_HPAI DOMAIN-CONTAINING PROTEIN (PTHR32308:SF0) | lyase | Halobacterium salinarum |
|  | **62.** | HALSA\|EnsemblGenome=VNG\_1230G\|UniProtKB=Q9HQC2 | VNG\_1230G | Chorismate synthase aroC PTN001078548 orthologs | CHORISMATE SYNTHASE (PTHR21085:SF0) | lyase | Halobacterium salinarum |
|  | **63.** | HALSA\|EnsemblGenome=VNG\_2471G\|UniProtKB=Q9HMM6 | VNG\_2471G | Probable cysteine desulfurase csd PTN000905733 orthologs | CYSTEINE DESULFURASE 1, CHLOROPLASTIC (PTHR43586:SF8) | lyase | Halobacterium salinarum |
|  | **64.** | HALSA\|EnsemblGenome=VNG\_1212G\|UniProtKB=Q9HQD5 | VNG\_1212G | Probable histidine ammonia-lyase hutH PTN001285070 orthologs | HISTIDINE AMMONIA-LYASE (PTHR10362:SF7) | lyase | Halobacterium salinarum |
|  | **65.** | HALSA\|EnsemblGenome=VNG\_1114G\|UniProtKB=Q9HQK8 | VNG\_1114G | Glyoxalase glo1 PTN000822295 orthologs | LACTOYLGLUTATHIONE LYASE (PTHR46036:SF5) | lyase | Halobacterium salinarum |
|  | **66.** | HALSA\|EnsemblGenome=VNG\_2137G\|UniProtKB=Q9HNE5 | VNG\_2137G | Lycopene beta-cyclase crtY PTN001585243 orthologs | PHYTOENE SYNTHASE, CHLOROPLASTIC (PTHR31480:SF2) | cyclase | Halobacterium salinarum |
|  | **67.** | HALSA\|EnsemblGenome=VNG\_1458G\|UniProtKB=Q9HPV3 | VNG\_1458G | Phytoene synthase crtB1 PTN001266710 orthologs | PHYTOENE SYNTHASE, CHLOROPLASTIC (PTHR31480:SF2) | cyclase | Halobacterium salinarum |
|  | **68.** | HALSA\|EnsemblGenome=VNG\_1208G\|UniProtKB=Q9HQD8 | VNG\_1208G | Probable urocanate hydratase hutU PTN001284357 orthologs | UROCANATE HYDRATASE (PTHR12216:SF4) | hydratase | Halobacterium salinarum |
|  | **69.** | HALSA\|EnsemblGenome=VNG\_2195G\|UniProtKB=Q9HN94 | VNG\_2195G | Cytochrome c oxidase subunit II coxB2 PTN001113122 orthologs | BLL4481 PROTEIN (PTHR42838:SF2) | oxidoreductase | Halobacterium salinarum |
|  | **70.** | HALSA\|EnsemblGenome=VNG\_2115H\|UniProtKB=Q9HNG5 | VNG\_2115H | Glutaredoxin grx1 PTN002437946 orthologs | GLUTAREDOXIN-LIKE PROTEIN NRDH (PTHR34386:SF1) | oxidoreductase | Halobacterium salinarum |
|  | **71.** | HALSA\|EnsemblGenome=VNG\_0933G\|UniProtKB=Q9HQZ6 | VNG\_0933G | NADH-dependent flavin oxidoreductase yqjM PTN001113461 orthologs | NADPH DEHYDROGENASE C23G7.10C-RELATED (PTHR43303:SF4) | oxidoreductase | Halobacterium salinarum |
|  | **72.** | HALSA\|EnsemblGenome=VNG\_0830G\|UniProtKB=Q9HR73 | VNG\_0830G | Putative dimethyl sulfoxide reductase iron-sulfur subunit B dmsB PTN000941346 orthologs | PROTEIN NRFC HOMOLOG (PTHR43177:SF3) | oxidoreductase | Halobacterium salinarum |
|  | **73.** | HALSA\|EnsemblGenome=VNG\_6055G\|UniProtKB=O54596 | VNG\_6471G | CydB cydB\_1 PTN002018357 orthologs | CYTOCHROME BD2 SUBUNIT II (PTHR43141:SF4) | oxidoreductase | Halobacterium salinarum |
|  | **74.** | HALSA\|EnsemblGenome=VNG\_2413H\|UniProtKB=Q9HMS1 | VNG\_2413H | DoxX domain protein VNG\_2413H PTN005004957 orthologs | INNER MEMBRANE PROTEIN YPHA-RELATED (PTHR33452:SF1) | oxidoreductase | Halobacterium salinarum |
|  | **75.** | HALSA\|EnsemblGenome=VNG\_1455H\|UniProtKB=Q9HPV6 | VNG\_1455H | DoxX domain protein VNG\_1455H PTN002851019 orthologs | INNER MEMBRANE PROTEIN YPHA-RELATED (PTHR33452:SF1) | oxidoreductase | Halobacterium salinarum |
|  | **76.** | HALSA\|EnsemblGenome=VNG\_1972G\|UniProtKB=Q9HNS2 | VNG\_1972G | Glycerol-3-phosphate dehydrogenase chain C gpdC PTN001287044 orthologs | ANAEROBIC GLYCEROL-3-PHOSPHATE DEHYDROGENASE SUBUNIT C (PTHR32479:SF19) | oxidoreductase | Halobacterium salinarum |
|  | **77.** | HALSA\|EnsemblGenome=VNG\_1191G\|UniProtKB=Q9HQF0 | VNG\_1191G | Acyl-CoA dehydrogenase acd3 PTN000856565 orthologs | ISOVALERYL-COA DEHYDROGENASE, MITOCHONDRIAL-RELATED (PTHR43884:SF12) | dehydrogenase | Halobacterium salinarum |
|  | **78.** | HALSA\|EnsemblGenome=VNG\_1755G\|UniProtKB=Q9HP88 | VNG\_1755G | Phytoene dehydrogenase crtI2 PTN000839646 orthologs | PHYTOENE DESATURASE (PTHR43734:SF1) | dehydrogenase | Halobacterium salinarum |
|  | **79.** | HALSA\|EnsemblGenome=VNG\_1971G\|UniProtKB=Q9HNS3 | VNG\_1971G | Probable anaerobic glycerol-3-phosphate dehydrogenase subunit B glpB PTN005162388 orthologs | ANAEROBIC GLYCEROL-3-PHOSPHATE DEHYDROGENASE SUBUNIT B (PTHR43400:SF11) | dehydrogenase | Halobacterium salinarum |
|  | **80.** | HALSA\|EnsemblGenome=VNG\_0281G\|UniProtKB=Q9HSD9 | VNG\_0281G | FAD-dependent oxidoreductase soxB PTN001014856 orthologs | FAD-DEPENDENT OXIDOREDUCTASE DOMAIN-CONTAINING PROTEIN 1 (PTHR13847:SF287) | dehydrogenase | Halobacterium salinarum |
|  | **81.** | HALSA\|EnsemblGenome=VNG\_0628G\|UniProtKB=Q9HRM7 | VNG\_0628G | NADP-specific glutamate dehydrogenase A1 gdhA1 PTN000906183 orthologs | GLUTAMATE DEHYDROGENASE 1, MITOCHONDRIAL (PTHR11606:SF13) | dehydrogenase | Halobacterium salinarum |
|  | **82.** | HALSA\|EnsemblGenome=VNG\_2171G\|UniProtKB=Q9HNB5 | VNG\_2171G | Alcohol dehydrogenase adh1 PTN002608691 orthologs | HYDROXYACID-OXOACID TRANSHYDROGENASE, MITOCHONDRIAL (PTHR11496:SF83) | dehydrogenase | Halobacterium salinarum |
|  | **83.** | HALSA\|EnsemblGenome=VNG\_1969G\|UniProtKB=Q9HNS4 | VNG\_1969G | Glycerol-3-phosphate dehydrogenase gpdA2 PTN000944713 orthologs | GLYCEROL-3-PHOSPHATE DEHYDROGENASE, MITOCHONDRIAL (PTHR11985:SF15) | dehydrogenase | Halobacterium salinarum |
|  | **84.** | HALSA\|EnsemblGenome=VNG\_0775G\|UniProtKB=Q9HRB3 | VNG\_0775G | Acyl-CoA dehydrogenase acd2 PTN000856567 orthologs | ISOVALERYL-COA DEHYDROGENASE, MITOCHONDRIAL-RELATED (PTHR43884:SF12) | dehydrogenase | Halobacterium salinarum |
|  | **85.** | HALSA\|EnsemblGenome=VNG\_0406C\|UniProtKB=Q9HS49 | VNG\_0406C | Glycerol-1-phosphate dehydrogenase [NAD(P)+] egsA PTN001691336 orthologs | GLYCEROL DEHYDROGENASE 1 (PTHR43616:SF5) | dehydrogenase | Halobacterium salinarum |
|  | **86.** | HALSA\|EnsemblGenome=VNG\_2193G\|UniProtKB=Q9HN95 | VNG\_2193G | Cytochrome c oxidase subunit I coxA1 PTN000823925 orthologs | CYTOCHROME C OXIDASE SUBUNIT I (PTHR10422:SF40) | oxidase | Halobacterium salinarum |
|  | **87.** | HALSA\|EnsemblGenome=VNG\_0930G\|UniProtKB=Q9HQZ9 | VNG\_0930G | Alkanal monooxygenase homolog yvbT PTN001248673 orthologs | LUCIFERASE-LIKE MONOOXYGENASE (PTHR30137:SF6) | oxygenase | Halobacterium salinarum |
|  | **88.** | HALSA\|EnsemblGenome=VNG\_1404G\|UniProtKB=Q9HPZ3 | VNG\_1404G | Peptide methionine sulfoxide reductase MsrB(R-form specific) trh1 PTN000808173 orthologs | METHIONINE-R-SULFOXIDE REDUCTASE B1 (PTHR10173:SF52) | reductase | Halobacterium salinarum |
|  | **89.** | HALSA\|EnsemblGenome=VNG\_1364G\|UniProtKB=Q9HQ24 | VNG\_1364G | Ornithine cyclodeaminase ocd2 PTN001013599 orthologs | KETIMINE REDUCTASE MU-CRYSTALLIN (PTHR13812:SF19) | reductase | Halobacterium salinarum |
|  | **90.** | HALSA\|EnsemblGenome=VNG\_0829G\|UniProtKB=Q9HR74 | VNG\_0829G | Putative dimethyl sulfoxide reductase catalytic subunit A dmsA PTN000906916 orthologs | OXIDOREDUCTASE YYAE-RELATED (PTHR43742:SF6) | reductase | Halobacterium salinarum |
|  | **91.** | HALSA\|EnsemblGenome=VNG\_1644G\|UniProtKB=Q9HPG8 | VNG\_1644G | Vitamin B12-dependent ribonucleotide reductase nrdB2 PTN000902996 orthologs | RIBONUCLEOSIDE-DIPHOSPHATE REDUCTASE (PTHR43371:SF1) | reductase | Halobacterium salinarum |
|  | **92.** | HALSA\|EnsemblGenome=VNG\_0935G\|UniProtKB=Q9HQZ4 | VNG\_0935G | NADH oxidase noxC PTN001125855 orthologs | IODOTYROSINE DEIODINASE 1 (PTHR23026:SF90) | reductase | Halobacterium salinarum |
|  | **93.** | HALSA\|EnsemblGenome=VNG\_2394G\|UniProtKB=Q9HMT6 | VNG\_2394G | Rhodanese domain protein tssB PTN000889595 orthologs | THIOSULFATE SULFURTRANSFERASE (PTHR43855:SF1) | transferase | Halobacterium salinarum |
|  | **94.** | HALSA\|EnsemblGenome=VNG\_1882G\|UniProtKB=Q9HNZ1 | VNG\_1882G | Quinolinate synthase A nadA PTN001254901 orthologs | QUINOLINATE SYNTHASE, CHLOROPLASTIC (PTHR30573:SF0) | transferase | Halobacterium salinarum |
|  | **95.** | HALSA\|EnsemblGenome=VNG\_1574G\|UniProtKB=Q9HPL6 | VNG\_1574G | Cob(I)alamin adenosyltransferase cobI PTN001247759 orthologs | CORRINOID ADENOSYLTRANSFERASE (PTHR46638:SF1) | transferase | Halobacterium salinarum |
|  | **96.** | HALSA\|EnsemblGenome=VNG\_2102G\|UniProtKB=Q9HNH4 | VNG\_2102G | Citrate synthase citZ PTN000921858 orthologs | CITRATE SYNTHASE, PEROXISOMAL (PTHR11739:SF4) | transferase | Halobacterium salinarum |
|  | **97.** | HALSA\|EnsemblGenome=VNG\_0654C\|UniProtKB=Q9HRK6 | VNG\_0654C | GNAT family acetyltransferase VNG\_0654C PTN002453853 orthologs | GCN5-RELATED N-ACETYLTRANSFERASE (PTHR43072:SF52) | acetyltransferase | Halobacterium salinarum |
|  | **98.** | HALSA\|EnsemblGenome=VNG\_1781C\|UniProtKB=Q9HP67 | VNG\_1781C | Uncharacterized protein VNG\_1781C PTN000816032 orthologs | UNDECAPRENYL DIPHOSPHATE SYNTHASE (PTHR10291:SF28) | acyltransferase | Halobacterium salinarum |
|  | **99.** | HALSA\|EnsemblGenome=VNG\_1066C\|UniProtKB=Q9HQP4 | VNG\_1066C | Glycosyltransferase AglI aglI PTN001116001 orthologs | GLYCO\_TRANS\_2-LIKE DOMAIN-CONTAINING PROTEIN (PTHR43685:SF2) | glycosyltransferase | Halobacterium salinarum |
|  | **100.** | HALSA\|EnsemblGenome=VNG\_6316G\|UniProtKB=Q48295 | VNG\_6316G | Carbamate kinase arcC PTN001252230 orthologs | CARBAMATE KINASE-RELATED (PTHR30409:SF1) | kinase | Halobacterium salinarum |
|  | **101.** | HALSA\|EnsemblGenome=VNG\_1900C\|UniProtKB=Q9HNX7 | VNG\_1900C | NAD kinase nadK PTN001072970 orthologs | BIFUNCTIONAL NADP PHOSPHATASE/NAD KINASE (PTHR20275:SF43) | nucleotide kinase | Halobacterium salinarum |
|  | **102.** | HALSA\|EnsemblGenome=VNG\_1568G\|UniProtKB=Q9HPM0 | VNG\_1568G | Cobalt-precorrin-7 C5-methyltransferase cbiJ PTN001329534 orthologs | COBALT-PRECORRIN-7 C(5)-METHYLTRANSFERASE (PTHR43182:SF1) | methyltransferase | Halobacterium salinarum |
|  | **103.** | HALSA\|EnsemblGenome=VNG\_2514G\|UniProtKB=Q9HMJ5 | VNG\_2514G | 30S ribosomal protein S6e rps6e PTN000896942 orthologs | 40S RIBOSOMAL PROTEIN S6 (PTHR11502:SF6) | ribosomal protein | Halobacterium salinarum |
|  | **104.** | HALSA\|EnsemblGenome=VNG\_1190G\|UniProtKB=P09737 | VNG\_1190G | Superoxide dismutase [Mn] 1 sod1 PTN000891878 orthologs | 37S RIBOSOMAL PROTEIN S26, MITOCHONDRIAL (PTHR43595:SF2) | ribosomal protein | Halobacterium salinarum |
|  | **105.** | HALSA\|EnsemblGenome=VNG\_1464G\|UniProtKB=Q9HPU8 | VNG\_1464G | Bacterioopsin transcriptional activator bat PTN001968220 orthologs | BACTERIOOPSIN TRANSCRIPTIONAL ACTIVATOR (PTHR46175:SF4) | DNA-binding transcription factor | Halobacterium salinarum |
|  | **106.** | HALSA\|EnsemblGenome=VNG\_0826C\|UniProtKB=Q9HR76 | VNG\_0826C | Dimethyl sulfoxide reductase transcriptional activator dmsR PTN002151777 orthologs | DIMETHYL SULFOXIDE REDUCTASE TRANSCRIPTIONAL ACTIVATOR (PTHR34236:SF1) | DNA-binding transcription factor | Halobacterium salinarum |
|  | **107.** | HALSA\|EnsemblGenome=VNG\_0794G\|UniProtKB=Q9HRA0 | VNG\_0794G | ABC transporter (Lipoprotein) yufN PTN002153416 orthologs | ABC TRANSPORTER GUANOSINE-BINDING PROTEIN NUPN (PTHR34296:SF2) | DNA-binding transcription factor | Halobacterium salinarum |
|  | **108.** | HALSA\|EnsemblGenome=VNG\_0852C\|UniProtKB=Q9HR58 | VNG\_0852C | HTH-10 family transcription regulator VNG\_0852C PTN002151778 orthologs | DIMETHYL SULFOXIDE REDUCTASE TRANSCRIPTIONAL ACTIVATOR (PTHR34236:SF1) | DNA-binding transcription factor | Halobacterium salinarum |
|  | **109.** | HALSA\|EnsemblGenome=VNG\_0996G\|UniProtKB=Q9HQU9 | VNG\_0996G | Bacterio-opsin activator-like protein boa4 PTN002151780 orthologs | DIMETHYL SULFOXIDE REDUCTASE TRANSCRIPTIONAL ACTIVATOR (PTHR34236:SF1) | DNA-binding transcription factor | Halobacterium salinarum |
|  | **110.** | HALSA\|EnsemblGenome=VNG\_1285G\|UniProtKB=Q9HQ80 | VNG\_1285G | Lrp/AsnC family transcription regulator / TrkAdomain protein trh2 PTN001575286 orthologs | TRANSCRIPTIONAL REGULATOR AZLB (PTHR30154:SF34) | helix-turn-helix transcription factor | Halobacterium salinarum |
|  | **111.** | HALSA\|EnsemblGenome=VNG\_1886C\|UniProtKB=Q9HNY8 | VNG\_1886C | TrmB family transcription regulator VNG\_1886C PTN002153297 orthologs | HTH-TYPE TRANSCRIPTIONAL REGULATOR TRMBL2 (PTHR34293:SF1) | helix-turn-helix transcription factor | Halobacterium salinarum |
|  | **112.** | HALSA\|EnsemblGenome=VNG\_0536G\|UniProtKB=Q9HRU8 | VNG\_0536G | Transcription repressor sirR PTN002114785 orthologs | TRANSCRIPTIONAL REGULATOR MNTR (PTHR33238:SF7) | winged helix/forkhead transcription factor | Halobacterium salinarum |
|  | **113.** | HALSA\|EnsemblGenome=VNG\_2385H\|UniProtKB=Q9HMU2 | VNG\_2385H | Uncharacterized protein VNG\_2385H  orthologs | - | - | Halobacterium salinarum |
|  | **114.** | HALSA\|EnsemblGenome=VNG\_1657H\|UniProtKB=Q9HPF9 | VNG\_1657H | Uncharacterized protein VNG\_1657H  orthologs | - | - | Halobacterium salinarum |
|  | **115.** | HALSA\|EnsemblGenome=VNG\_1831H\|UniProtKB=Q9HP31 | VNG\_1831H | Uncharacterized protein VNG\_1831H  orthologs | - | - | Halobacterium salinarum |
|  | **116.** | HALSA\|EnsemblGenome=VNG\_0849C\|UniProtKB=Q9HR60 | VNG\_0849C | Probable coiled coil protein VNG\_0849C  orthologs | - | - | Halobacterium salinarum |
|  | **117.** | HALSA\|EnsemblGenome=VNG\_0391C\|UniProtKB=Q9HS60 | VNG\_0391C | Diphthine--ammonia ligase dph6 PTN000953804 orthologs | DIPHTHINE--AMMONIA LIGASE (PTHR12196:SF2) | - | Halobacterium salinarum |
|  | **118.** | HALSA\|EnsemblGenome=VNG\_1953C\|UniProtKB=Q9HNT5 | VNG\_1953C | Probable secreted glycoprotein VNG\_1953C  orthologs | - | - | Halobacterium salinarum |
|  | **119.** | HALSA\|EnsemblGenome=VNG\_1660G\|UniProtKB=P0DMH8 | VNG\_1660G | Sensory rhodopsin-1 sop1 PTN002408016 orthologs | BACTERIORHODOPSIN /OPSIN, NOPA (EUROFUNG)-RELATED (PTHR28286:SF2) | - | Halobacterium salinarum |
|  | **120.** | HALSA\|EnsemblGenome=VNG\_1973H\|UniProtKB=Q9HNS1 | VNG\_1973H | Uncharacterized protein VNG\_1973H  orthologs | - | - | Halobacterium salinarum |
|  | **121.** | HALSA\|EnsemblGenome=VNG\_0557H\|UniProtKB=Q9HRT2 | VNG\_0557H | ThiJ/PfpI domain protein VNG\_0557H PTN000866703 orthologs | GLUTATHIONE-INDEPENDENT GLYOXALASE HSP31-RELATED (PTHR48094:SF11) | - | Halobacterium salinarum |
|  | **122.** | HALSA\|EnsemblGenome=VNG\_0703H\|UniProtKB=Q9HRH0 | VNG\_0703H | NP\_1176A family transcription regulator VNG\_0703H  orthologs | - | - | Halobacterium salinarum |
|  | **123.** | HALSA\|EnsemblGenome=VNG\_1475C\|UniProtKB=Q9HPT8 | VNG\_1475C | CARDB domain-containing protein VNG\_1475C PTN002185796 orthologs | CONSERVED WITHIN P. AEROPHILUM (PTHR35902:SF3) | - | Halobacterium salinarum |
|  | **124.** | HALSA\|EnsemblGenome=VNG\_2497H\|UniProtKB=Q9HMK9 | VNG\_2497H | Cupin\_2 domain-containing protein VNG\_2497H PTN002185050 orthologs | CUPIN\_2 DOMAIN-CONTAINING PROTEIN (PTHR35848:SF6) | - | Halobacterium salinarum |
|  | **125.** | HALSA\|EnsemblGenome=VNG\_1566G\|UniProtKB=Q9HPM2 | VNG\_1566G | Cobalamin biosynthesis protein cobN PTN001258368 orthologs | MAGNESIUM CHELATASE SUBUNIT (PTHR44119:SF7) | - | Halobacterium salinarum |
|  | **126.** | HALSA\|EnsemblGenome=VNG\_1637G\|UniProtKB=Q9HPH3 | VNG\_1637G | Halocyanin HcpA hcpA PTN002150135 orthologs | PLASTOCYANIN MAJOR ISOFORM, CHLOROPLASTIC-RELATED (PTHR34192:SF10) | - | Halobacterium salinarum |
|  | **127.** | HALSA\|EnsemblGenome=VNG\_1822G\|UniProtKB=Q9HP37 | VNG\_1822G | Molybdenum cofactor biosynthesis protein moaB PTN000810231 orthologs | MOLYBDENUM COFACTOR BIOSYNTHESIS PROTEIN B (PTHR43232:SF2) | - | Halobacterium salinarum |
|  | **128.** | HALSA\|EnsemblGenome=VNG\_6095C\|UniProtKB=O51976 | VNG\_6095C | Vng6095c VNG\_6095C PTN001784087 orthologs | ABASIC SITE PROCESSING PROTEIN HMCES (PTHR13604:SF0) | - | Halobacterium salinarum |
|  | **129.** | HALSA\|EnsemblGenome=VNG\_0192G\|UniProtKB=P0DMR7 | VNG\_0192G | Cell division protein FtsZ 2 ftsZ2 PTN002410867 orthologs | CELL DIVISION PROTEIN FTSZ 2 (PTHR30314:SF9) | - | Halobacterium salinarum |
|  | **130.** | HALSA\|EnsemblGenome=VNG\_1907H\|UniProtKB=Q9HNX0 | VNG\_1907H | Uncharacterized protein VNG\_1907H  orthologs | - | - | Halobacterium salinarum |
|  | **131.** | HALSA\|EnsemblGenome=VNG\_2523H\|UniProtKB=Q9HMI7 | VNG\_2523H | Usp domain-containing protein VNG\_2523H  orthologs | - | - | Halobacterium salinarum |
|  | **132.** | HALSA\|EnsemblGenome=VNG\_0743H\|UniProtKB=Q9HRD8 | VNG\_0743H | Usp domain-containing protein VNG\_0743H PTN008197798 orthologs | UNIVERSAL STRESS PROTEIN A-RELATED (PTHR46268:SF6) | - | Halobacterium salinarum |
|  | **133.** | HALSA\|EnsemblGenome=VNG\_6184G\|UniProtKB=Q9HHX5 | VNG\_6184G | Cationic amino acid transporter cat4  orthologs | - | - | Halobacterium salinarum |
|  | **134.** | HALSA\|EnsemblGenome=VNG\_1185G\|UniProtKB=Q9HQF5 | VNG\_1185G | Coenzyme PQQ synthesis protein pqqE PTN000881317 orthologs | TUNGSTEN-CONTAINING ALDEHYDE FERREDOXIN OXIDOREDUCTASE COFACTOR MODIFYING PROTEIN (PTHR11228:SF34) | - | Halobacterium salinarum |
|  | **135.** | HALSA\|EnsemblGenome=VNG\_1085H\|UniProtKB=Q9HQN0 | VNG\_1085H | Uncharacterized protein VNG\_1085H  orthologs | - | - | Halobacterium salinarum |
|  | **136.** | HALSA\|EnsemblGenome=VNG\_0995H\|UniProtKB=Q9HQV0 | VNG\_0995H | Small CPxCG-related zinc finger protein VNG\_0995H  orthologs | - | - | Halobacterium salinarum |
|  | **137.** | HALSA\|EnsemblGenome=VNG\_0227H\|UniProtKB=Q9HSH5 | VNG\_0227H | Uncharacterized protein VNG\_0227H  orthologs | - | - | Halobacterium salinarum |
|  | **138.** | HALSA\|EnsemblGenome=VNG\_1250H\|UniProtKB=Q9HQA6 | VNG\_1250H | Uncharacterized protein VNG\_1250H  orthologs | - | - | Halobacterium salinarum |
|  | **139.** | HALSA\|EnsemblGenome=VNG\_1609C\|UniProtKB=Q9HPJ4 | VNG\_1609C | DUF124 family protein VNG\_1609C PTN001273875 orthologs | ALTERED INHERITANCE OF MITOCHONDRIA PROTEIN 24, MITOCHONDRIAL (PTHR31801:SF0) | - | Halobacterium salinarum |
|  | **140.** | HALSA\|EnsemblGenome=VNG\_1890H\|UniProtKB=Q9HNY5 | VNG\_1890H | Uncharacterized protein VNG\_1890H  orthologs | - | - | Halobacterium salinarum |
|  | **141.** | HALSA\|EnsemblGenome=VNG\_1751H\|UniProtKB=Q9HP91 | VNG\_1751H | Receiver/sensor box protein VNG\_1751H  orthologs | - | - | Halobacterium salinarum |
|  | **142.** | HALSA\|EnsemblGenome=VNG\_1002H\|UniProtKB=Q9HQU3 | VNG\_1002H | Uncharacterized protein VNG\_1002H  orthologs | - | - | Halobacterium salinarum |
|  | **143.** | HALSA\|EnsemblGenome=VNG\_1425H\|UniProtKB=Q9HPX8 | VNG\_1425H | Uncharacterized protein VNG\_1425H  orthologs | - | - | Halobacterium salinarum |
|  | **144.** | HALSA\|EnsemblGenome=VNG\_1326H\|UniProtKB=Q9HQ51 | VNG\_1326H | Cupin\_2 domain-containing protein VNG\_1326H PTN002185049 orthologs | CUPIN\_2 DOMAIN-CONTAINING PROTEIN (PTHR35848:SF6) | - | Halobacterium salinarum |
|  | **145.** | HALSA\|EnsemblGenome=VNG\_2196G\|UniProtKB=Q9HN93 | VNG\_2196G | Halocyanin-like hcpB PTN002150136 orthologs | PLASTOCYANIN MAJOR ISOFORM, CHLOROPLASTIC-RELATED (PTHR34192:SF10) | - | Halobacterium salinarum |
|  | **146.** | HALSA\|EnsemblGenome=VNG\_1589C\|UniProtKB=Q9HPK6 | VNG\_1589C | Uncharacterized protein VNG\_1589C  orthologs | - | - | Halobacterium salinarum |
|  | **147.** | HALSA\|Gene=O52017\_HALSA\|UniProtKB=O52017 | VNG\_7113 | Spurious ORF VNG\_7113  orthologs | - | - | Halobacterium salinarum |
|  | **148.** | HALSA\|EnsemblGenome=VNG\_6087C\|UniProtKB=O54654 | VNG\_6087C | DUF1814 family protein VNG\_6087C  orthologs | - | - | Halobacterium salinarum |
|  | **149.** | HALSA\|EnsemblGenome=VNG\_1086C\|UniProtKB=Q9HQM9 | VNG\_1086C | UPF0058 family protein VNG\_1086C PTN002223879 orthologs | UPF0058 PROTEIN MJ1205 (PTHR42203:SF2) | - | Halobacterium salinarum |
|  | **150.** | HALSA\|EnsemblGenome=VNG\_1655H\|UniProtKB=Q9HPG0 | VNG\_1655H | Uncharacterized protein VNG\_1655H  orthologs | - | - | Halobacterium salinarum |
|  | **151.** | HALSA\|EnsemblGenome=VNG\_6305C\|UniProtKB=Q9HHN7 | VNG\_6305C | 7-carboxy-7-deazaguanine synthase queE PTN000881349 orthologs | 7-CARBOXY-7-DEAZAGUANINE SYNTHASE (PTHR42836:SF1) | - | Halobacterium salinarum |
|  | **152.** | HALSA\|EnsemblGenome=VNG\_1765G\|UniProtKB=Q9HP81 | VNG\_1765G | Sensory rhodopsin II transducer htr2 PTN001280265 orthologs | LYSOZYME-LIKE PROTEIN-RELATED (PTHR32089:SF92) | - | Halobacterium salinarum |
|  | **153.** | HALSA\|EnsemblGenome=VNG\_0708H\|UniProtKB=Q9HRG6 | VNG\_0708H | Uncharacterized protein VNG\_0708H  orthologs | - | - | Halobacterium salinarum |
|  | **154.** | HALSA\|EnsemblGenome=VNG\_6132G\|UniProtKB=O51999 | VNG\_6132G | Plasmid replication protein RepI repI  orthologs | - | - | Halobacterium salinarum |
|  | **155.** | HALSA\|EnsemblGenome=VNG\_6306C\|UniProtKB=Q9HHN6 | VNG\_6306C | 6-carboxy-5,6,7,8-tetrahydropterin synthase queD PTN000971842 orthologs | 6-PYRUVOYL TETRAHYDROBIOPTERIN SYNTHASE (PTHR12589:SF7) | - | Halobacterium salinarum |
|  | **156.** | HALSA\|EnsemblGenome=VNG\_6251H\|UniProtKB=Q9HHS5 | VNG\_6251H | DUF2078 family protein VNG\_6251H  orthologs | - | - | Halobacterium salinarum |
|  | **157.** | HALSA\|EnsemblGenome=VNG\_0592G\|UniProtKB=Q9HRQ5 | VNG\_0592G | Endonuclease III nthA2 PTN000820735 orthologs | ENDONUCLEASE III (PTHR10359:SF18) | - | Halobacterium salinarum |
|  | **158.** | HALSA\|EnsemblGenome=VNG\_0874G\|UniProtKB=Q9HR41 | VNG\_0874G | Possible signaling protein traB PTN001089257 orthologs | TRAB DOMAIN-CONTAINING PROTEIN (PTHR21530:SF7) | - | Halobacterium salinarum |
|  | **159.** | HALSA\|EnsemblGenome=VNG\_0729H\|UniProtKB=Q9HRF1 | VNG\_0729H | Uncharacterized protein VNG\_0729H  orthologs | - | - | Halobacterium salinarum |
|  | **160.** | HALSA\|EnsemblGenome=VNG\_1977H\|UniProtKB=Q9HNR8 | VNG\_1977H | AAA-4 family protein VNG\_1977H  orthologs | - | - | Halobacterium salinarum |
|  | **161.** | HALSA\|EnsemblGenome=VNG\_0597H\|UniProtKB=Q9HRQ0 | VNG\_0597H | Glycine-rich protein VNG\_0597H  orthologs | - | - | Halobacterium salinarum |
|  | **162.** | HALSA\|EnsemblGenome=VNG\_2644C\|UniProtKB=Q9HM94 | VNG\_2644C | Probable SepF protein sepF  orthologs | - | - | Halobacterium salinarum |
|  | **163.** | HALSA\|EnsemblGenome=VNG\_1744H\|UniProtKB=Q9HP95 | VNG\_1744H | Uncharacterized protein VNG\_1744H  orthologs | - | - | Halobacterium salinarum |
|  | **164.** | HALSA\|EnsemblGenome=VNG\_1173G\|UniProtKB=Q9HQG6 | VNG\_1173G | Elongation factor 1-beta ef1b PTN002218754 orthologs | ELONGATION FACTOR 1-BETA (PTHR39647:SF1) | - | Halobacterium salinarum |
|  | **165.** | HALSA\|EnsemblGenome=VNG\_1479H\|UniProtKB=Q9HPT5 | VNG\_1479H | Uncharacterized protein VNG\_1479H  orthologs | - | - | Halobacterium salinarum |
|  | **166.** | HALSA\|EnsemblGenome=VNG\_1372C\|UniProtKB=Q9HQ17 | VNG\_1372C | Probable secreted glycoprotein VNG\_1372C  orthologs | - | - | Halobacterium salinarum |
|  | **167.** | HALSA\|EnsemblGenome=VNG\_0195H\|UniProtKB=Q9HSJ8 | VNG\_0195H | Double zinc ribbon protein VNG\_0195H VNG\_0195H  orthologs | - | - | Halobacterium salinarum |
|  | **168.** | HALSA\|EnsemblGenome=VNG\_1857C\|UniProtKB=Q9HP09 | VNG\_1857C | Chemotactic signal transduction systemperiplasmic substrate-binding protein BasB basB PTN001253437 orthologs | PERIPLASMIC BINDING PROTEIN OF ABC TRANSPORTER FOR NATURAL AMINO ACIDS (PTHR30483:SF6) | - | Halobacterium salinarum |
|  | **169.** | HALSA\|EnsemblGenome=VNG\_1193C\|UniProtKB=Q9HQE9 | VNG\_1193C | Histidine kinase VNG\_1193C PTN001564549 orthologs | SENSOR PROTEIN RSTB (PTHR44936:SF9) | - | Halobacterium salinarum |
|  | **170.** | HALSA\|EnsemblGenome=VNG\_1238C\|UniProtKB=Q9HQB4 | VNG\_1238C | Uncharacterized protein VNG\_1238C  orthologs | - | - | Halobacterium salinarum |
|  | **171.** | HALSA\|EnsemblGenome=VNG\_1659G\|UniProtKB=P0DMI3 | VNG\_1659G | Sensory rhodopsin I transducer htr1 PTN002424866 orthologs | LYSOZYME-LIKE PROTEIN-RELATED (PTHR32089:SF92) | - | Halobacterium salinarum |
|  | **172.** | HALSA\|EnsemblGenome=VNG\_2493C\|UniProtKB=Q9HML2 | VNG\_2493C | PHP domain protein VNG\_2493C PTN001246493 orthologs | POLIIIAC DOMAIN-CONTAINING PROTEIN (PTHR42924:SF3) | - | Halobacterium salinarum |
|  | **173.** | HALSA\|EnsemblGenome=VNG\_0258H\|UniProtKB=Q9HSF4 | VNG\_0258H | PadR family transcription regulator RosR rosR  orthologs | - | - | Halobacterium salinarum |
|  | **174.** | HALSA\|EnsemblGenome=VNG\_6323H\|UniProtKB=Q9HHM4 | VNG\_6323H | Vng6323h VNG\_6323H  orthologs | - | - | Halobacterium salinarum |
|  | **175.** | HALSA\|EnsemblGenome=VNG\_6255C\|UniProtKB=Q9HHS3 | VNG\_6255C | Vng6255c VNG\_6255C  orthologs | - | - | Halobacterium salinarum |
|  | **176.** | HALSA\|EnsemblGenome=VNG\_0280H\|UniProtKB=Q9HSE0 | VNG\_0280H | Spurious ORF VNG\_0280H  orthologs | - | - | Halobacterium salinarum |
|  | **177.** | HALSA\|EnsemblGenome=VNG\_2321G\|UniProtKB=Q9HMZ2 | VNG\_2321G | GNAT family acetyltransferase ydaJ PTN001272805 orthologs | [RIBOSOMAL PROTEIN S5]-ALANINE N-ACETYLTRANSFERASE (PTHR43792:SF8) | - | Halobacterium salinarum |
|  | **178.** | HALSA\|EnsemblGenome=VNG\_0447H\|UniProtKB=Q9HS18 | VNG\_0447H | HAD superfamily hydrolase VNG\_0447H  orthologs | - | - | Halobacterium salinarum |
|  | **179.** | HALSA\|EnsemblGenome=VNG\_1758H\|UniProtKB=Q9HP86 | VNG\_1758H | Uncharacterized protein VNG\_1758H  orthologs | - | - | Halobacterium salinarum |
|  | **180.** | HALSA\|EnsemblGenome=VNG\_2191H\|UniProtKB=Q9HN96 | VNG\_2191H | Uncharacterized protein VNG\_2191H  orthologs | - | - | Halobacterium salinarum |
|  | **181.** | HALSA\|EnsemblGenome=VNG\_6429H\|UniProtKB=Q9HHF5 | VNG\_6429H | Spurious ORF VNG\_6429H  orthologs | - | - | Halobacterium salinarum |
|  | **182.** | HALSA\|EnsemblGenome=VNG\_6327H\|UniProtKB=Q9HHM1 | VNG\_6327H | Vng6327h VNG\_6327H  orthologs | - | - | Halobacterium salinarum |
|  | **183.** | HALSA\|EnsemblGenome=VNG\_6297C\|UniProtKB=Q9HHP3 | VNG\_6297C | PQQ repeat protein VNG\_6297C PTN002844908 orthologs | OUTER MEMBRANE PROTEIN ASSEMBLY FACTOR BAMB (PTHR34512:SF30) | - | Halobacterium salinarum |
|  | **184.** | HALSA\|EnsemblGenome=VNG\_2168C\|UniProtKB=Q9HNB6 | VNG\_2168C | Zinc metalloprotease VNG\_2168C PTN000936582 orthologs | ZINC METALLOPROTEASE (PTHR48108:SF19) | - | Halobacterium salinarum |
|  | **185.** | HALSA\|EnsemblGenome=VNG\_1642H\|UniProtKB=Q9HPG9 | VNG\_1642H | Small CPxCG-related zinc finger protein VNG\_1642H  orthologs | - | - | Halobacterium salinarum |
|  | **186.** | HALSA\|EnsemblGenome=VNG\_1115H\|UniProtKB=Q9HQK7 | VNG\_1115H | Probable S-adenosyl-L-methionine-binding protein VNG\_1115H VNG\_1115H  orthologs | - | - | Halobacterium salinarum |
|  | **187.** | HALSA\|EnsemblGenome=VNG\_0992H\|UniProtKB=Q9HQV2 | VNG\_0992H | Uncharacterized protein VNG\_0992H  orthologs | - | - | Halobacterium salinarum |
|  | **188.** | HALSA\|EnsemblGenome=VNG\_0991H\|UniProtKB=Q9HQV3 | VNG\_0991H | Uncharacterized protein VNG\_0991H  orthologs | - | - | Halobacterium salinarum |
|  | **189.** | HALSA\|EnsemblGenome=VNG\_6441H\|UniProtKB=Q9HHE7 | VNG\_6441H | Vng6441h VNG\_6441H PTN002211752 orthologs | PILIN\_N DOMAIN-CONTAINING PROTEIN (PTHR38138:SF1) | - | Halobacterium salinarum |
|  | **190.** | HALSA\|EnsemblGenome=VNG\_6265G\|UniProtKB=Q9HHR6 | VNG\_6265G | ABC-type transport system periplasmicsubstrate-binding protein (Probable substrate zinc) ycdH PTN001253234 orthologs | HIGH-AFFINITY ZINC UPTAKE SYSTEM PROTEIN ZNUA (PTHR42953:SF3) | - | Halobacterium salinarum |
|  | **191.** | HALSA\|EnsemblGenome=VNG\_0180G\|UniProtKB=P0DMH7 | VNG\_0180G | Halorhodopsin hop PTN002408017 orthologs | BACTERIORHODOPSIN /OPSIN, NOPA (EUROFUNG)-RELATED (PTHR28286:SF2) | - | Halobacterium salinarum |
|  | **192.** | HALSA\|EnsemblGenome=VNG\_1465G\|UniProtKB=Q9HPU7 | VNG\_1465G | Probable beta-carotene 15,15'-dioxygenase Brp brp  orthologs | - | - | Halobacterium salinarum |
|  | **193.** | HALSA\|EnsemblGenome=VNG\_0265G\|UniProtKB=Q9HSE9 | VNG\_0265G | Tubulin-like protein CetZ ftsZ4 PTN001250977 orthologs | TUBULIN-LIKE PROTEIN CETZ (PTHR30314:SF10) | - | Halobacterium salinarum |
|  | **194.** | HALSA\|EnsemblGenome=VNG\_1227H\|UniProtKB=Q9HQC6 | VNG\_1227H | Uncharacterized protein VNG\_1227H PTN002443121 orthologs | SLL1095 PROTEIN (PTHR36529:SF1) | - | Halobacterium salinarum |
|  | **195.** | HALSA\|EnsemblGenome=VNG\_0828H\|UniProtKB=Q9HR75 | VNG\_0828H | Ferredoxin\_N domain-containing protein VNG\_0828H  orthologs | - | - | Halobacterium salinarum |
|  | **196.** | HALSA\|EnsemblGenome=VNG\_0831G\|UniProtKB=Q9HR72 | VNG\_0831G | Putative dimethyl sulfoxide reductase membrane subunit C dmsC PTN002166624 orthologs | PROTEIN NRFD (PTHR34856:SF2) | - | Halobacterium salinarum |
|  | **197.** | HALSA\|EnsemblGenome=VNG\_6313G\|UniProtKB=Q9HHN1 | VNG\_6313G | Arginine/ornithine antiporter ArcD arcD PTN002125211 orthologs | MALATE-2H(+)/NA(+)-LACTATE ANTIPORTER (PTHR33451:SF3) | - | Halobacterium salinarum |
|  | **198.** | HALSA\|EnsemblGenome=VNG\_1189H\|UniProtKB=Q9HQF2 | VNG\_1189H | Uncharacterized protein VNG\_1189H  orthologs | - | - | Halobacterium salinarum |
|  | **199.** | HALSA\|Gene=O52005\_HALSA\|UniProtKB=O52005 | VNG\_7101 | YidE family protein VNG\_7101  orthologs | - | - | Halobacterium salinarum |
|  | **200.** | HALSA\|EnsemblGenome=VNG\_0999H\|UniProtKB=Q9HQU6 | VNG\_0999H | Uncharacterized protein VNG\_0999H  orthologs | - | - | Halobacterium salinarum |
|  | **201.** | HALSA\|EnsemblGenome=VNG\_1315H\|UniProtKB=Q9HQ59 | VNG\_1315H | Uncharacterized protein VNG\_1315H  orthologs | - | - | Halobacterium salinarum |
|  | **202.** | HALSA\|EnsemblGenome=VNG\_2412H\|UniProtKB=Q9HMS2 | VNG\_2412H | Uncharacterized protein VNG\_2412H  orthologs | - | - | Halobacterium salinarum |
|  | **203.** | HALSA\|EnsemblGenome=VNG\_1459H\|UniProtKB=Q9HPV2 | VNG\_1459H | Uncharacterized protein VNG\_1459H  orthologs | - | - | Halobacterium salinarum |
|  | **204.** | HALSA\|EnsemblGenome=VNG\_1476C\|UniProtKB=Q9HPT7 | VNG\_1476C | RND superfamily permease VNG\_1476C PTN002012819 orthologs | PROTEIN EXPORT MEMBRANE PROTEIN (PTHR30081:SF10) | - | Halobacterium salinarum |
|  | **205.** | HALSA\|EnsemblGenome=VNG\_0934H\|UniProtKB=Q9HQZ5 | VNG\_0934H | Uncharacterized protein VNG\_0934H  orthologs | - | - | Halobacterium salinarum |
|  | **206.** | HALSA\|EnsemblGenome=VNG\_0578H\|UniProtKB=Q9HRR6 | VNG\_0578H | Uncharacterized protein VNG\_0578H  orthologs | - | - | Halobacterium salinarum |
|  | **207.** | HALSA\|EnsemblGenome=VNG\_0220H\|UniProtKB=Q9HSH9 | VNG\_0220H | Uncharacterized protein VNG\_0220H  orthologs | - | - | Halobacterium salinarum |
|  | **208.** | HALSA\|EnsemblGenome=VNG\_0289H\|UniProtKB=Q9HSD2 | VNG\_0289H | Uncharacterized protein VNG\_0289H PTN002185326 orthologs | PUTATIVE-RELATED (PTHR35866:SF1) | - | Halobacterium salinarum |
|  | **209.** | HALSA\|EnsemblGenome=VNG\_6347H\|UniProtKB=Q9HHK8 | VNG\_6347H | Vng6347h VNG\_6347H  orthologs | - | - | Halobacterium salinarum |
|  | **210.** | HALSA\|EnsemblGenome=VNG\_1446H\|UniProtKB=Q9HPW4 | VNG\_1446H | Uncharacterized protein VNG\_1446H PTN002217358 orthologs | CALCIUM DODECIN (PTHR39324:SF1) | - | Halobacterium salinarum |
|  | **211.** | HALSA\|EnsemblGenome=VNG\_2589C\|UniProtKB=Q9HMD9 | VNG\_2589C | SIMPL domain protein VNG\_2589C PTN002155674 orthologs | SLR1258 PROTEIN (PTHR34387:SF2) | - | Halobacterium salinarum |
|  | **212.** | HALSA\|EnsemblGenome=VNG\_1572C\|UniProtKB=Q9HPL8 | VNG\_1572C | UPF0284 protein VNG\_1572C VNG\_1572C PTN002215596 orthologs | UPF0284 PROTEIN SLL1500 (PTHR38811:SF1) | - | Halobacterium salinarum |
|  | **213.** | HALSA\|EnsemblGenome=VNG\_2136G\|UniProtKB=Q9HNE6 | VNG\_2136G | Probable beta-carotene 15,15'-dioxygenase Blh blh  orthologs | - | - | Halobacterium salinarum |
|  | **214.** | HALSA\|EnsemblGenome=VNG\_0949G\|UniProtKB=Q9HQY3 | VNG\_0949G | Archaellar motor/biogenesis protein ArlI gspE3 PTN001253522 orthologs | FLAGELLA ACCESSORY PROTEIN I (PTHR30486:SF14) | - | Halobacterium salinarum |
|  | **215.** | HALSA\|EnsemblGenome=VNG\_1632G\|UniProtKB=Q9HPH6 | VNG\_1632G | ABC-type transport system permease protein(Probable substrate cobalt) cbiQ PTN002166657 orthologs | COBALT TRANSPORT PROTEIN CBIQ (PTHR43723:SF1) | - | Halobacterium salinarum |
|  | **216.** | HALSA\|EnsemblGenome=VNG\_1039H\|UniProtKB=Q9HQR4 | VNG\_1039H | Uncharacterized protein VNG\_1039H  orthologs | - | - | Halobacterium salinarum |
|  | **217.** | HALSA\|Gene=O52007\_HALSA\|UniProtKB=O52007 | VNG\_7103 | Uncharacterized protein VNG\_7103  orthologs | - | - | Halobacterium salinarum |
|  | **218.** | HALSA\|EnsemblGenome=VNG\_6036H\|UniProtKB=Q9HI15 | VNG\_6036H | Small CPxCG-related zinc finger protein VNG\_6036H  orthologs | - | - | Halobacterium salinarum |
|  | **219.** | HALSA\|EnsemblGenome=VNG\_1314H\|UniProtKB=Q9HQ60 | VNG\_1314H | Uncharacterized protein VNG\_1314H  orthologs | - | - | Halobacterium salinarum |
|  | **220.** | HALSA\|EnsemblGenome=VNG\_1985C\|UniProtKB=Q9HNR2 | VNG\_1985C | Probable NhaC-type sodium/proton antiporter nhaC3 PTN002125193 orthologs | NA\_H\_ANTIPORTER DOMAIN-CONTAINING PROTEIN (PTHR43478:SF1) | - | Halobacterium salinarum |
|  | **221.** | HALSA\|EnsemblGenome=VNG\_1101C\|UniProtKB=Q9HQL6 | VNG\_1101C | DUF112 family protein VNG\_1101C PTN002223886 orthologs | INTEGRAL MEMBRANE PROTEIN (PTHR42204:SF1) | - | Halobacterium salinarum |
|  | **222.** | HALSA\|EnsemblGenome=VNG\_2679G\|UniProtKB=P0DME1 | VNG\_2679G | Cell surface glycoprotein csg  orthologs | - | - | Halobacterium salinarum |
|  | **223.** | HALSA\|EnsemblGenome=VNG\_0184H\|UniProtKB=Q9HSK6 | VNG\_0184H | Methyltransf\_11 domain-containing protein VNG\_0184H  orthologs | - | - | Halobacterium salinarum |
|  | **224.** | HALSA\|EnsemblGenome=VNG\_2421G\|UniProtKB=Q9HMR3 | VNG\_2421G | O-acetyl homoserine hal PTN002887222 orthologs | HOMOCYSTEINE/CYSTEINE SYNTHASE (PTHR43797:SF2) | - | Halobacterium salinarum |
|  | **225.** | HALSA\|EnsemblGenome=VNG\_1576G\|UniProtKB=Q9HPL5 | VNG\_1576G | Probable cobyric acid synthase cobQ PTN001085035 orthologs | COBYRIC ACID SYNTHASE (PTHR21343:SF1) | - | Halobacterium salinarum |
|  | **226.** | HALSA\|EnsemblGenome=VNG\_1536C\|UniProtKB=Q9HPP5 | VNG\_1536C | UspA domain protein VNG\_1536C PTN001277844 orthologs | UNIVERSAL STRESS PROTEIN A-RELATED (PTHR46268:SF6) | - | Halobacterium salinarum |
|  | **227.** | HALSA\|EnsemblGenome=VNG\_2445C\|UniProtKB=Q9HMP5 | VNG\_2445C | ArsR family transcription regulator VNG\_2445C  orthologs | - | - | Halobacterium salinarum |
|  | **228.** | HALSA\|Gene=O52036\_HALSA\|UniProtKB=O52036 | VNG\_7132 | Uncharacterized protein VNG\_7132  orthologs | - | - | Halobacterium salinarum |
|  | **229.** | HALSA\|EnsemblGenome=VNG\_2109H\|UniProtKB=Q9HNG9 | VNG\_2109H | Uncharacterized protein VNG\_2109H  orthologs | - | - | Halobacterium salinarum |
|  | **230.** | HALSA\|EnsemblGenome=VNG\_1362H\|UniProtKB=Q9HQ25 | VNG\_1362H | DUF3054 family protein VNG\_1362H  orthologs | - | - | Halobacterium salinarum |
|  | **231.** | HALSA\|EnsemblGenome=VNG\_0793G\|UniProtKB=Q9HRA1 | VNG\_0793G | Htr6 transducer htr6 PTN001280264 orthologs | LYSOZYME-LIKE PROTEIN-RELATED (PTHR32089:SF92) | - | Halobacterium salinarum |
|  | **232.** | HALSA\|EnsemblGenome=VNG\_0355G\|UniProtKB=Q9HS86 | VNG\_0355G | Htr14 transducer htr14 PTN001280252 orthologs | LYSOZYME-LIKE PROTEIN-RELATED (PTHR32089:SF92) | - | Halobacterium salinarum |
|  | **233.** | HALSA\|EnsemblGenome=VNG\_1467G\|UniProtKB=P02945 | VNG\_1467G | Bacteriorhodopsin bop PTN002001478 orthologs | BACTERIORHODOPSIN /OPSIN, NOPA (EUROFUNG)-RELATED (PTHR28286:SF2) | - | Halobacterium salinarum |
|  | **234.** | HALSA\|EnsemblGenome=VNG\_1927H\|UniProtKB=Q9HNV5 | VNG\_1927H | Uncharacterized protein VNG\_1927H  orthologs | - | - | Halobacterium salinarum |
|  | **235.** | HALSA\|EnsemblGenome=VNG\_2288G\|UniProtKB=Q9HN20 | VNG\_2288G | Glutamate mutase epsilon subunit glmE  orthologs | - | - | Halobacterium salinarum |
|  | **236.** | HALSA\|EnsemblGenome=VNG\_1380H\|UniProtKB=Q9HQ11 | VNG\_1380H | Uncharacterized protein VNG\_1380H  orthologs | - | - | Halobacterium salinarum |
|  | **237.** | HALSA\|EnsemblGenome=VNG\_6155H\|UniProtKB=Q9HHZ6 | VNG\_6155H | Vng6155h VNG\_6155H  orthologs | - | - | Halobacterium salinarum |

  


|  |  |
| --- | --- |
| Hits 1-237 of 237 | [ page: (1) ] |

  
  
  
  
  


About   |  
Release Information   |  
Contact Us   |  
System Requirements   |  
Privacy Policy   |  
Disclaimer

© Copyright 2022 Paul Thomas All Rights Reserved.
