# Supplementary material for: Low Salt Influences Archaellum-Based Motility, Glycerol Metabolism, and Gas Vesicles Biogenesis in Halobacterium salinarum
Source: Microorganisms. 2022 Dec 10;10(12):2442. doi: 10.3390/microorganisms10122442 (PMC9786353; doi:10.3390/microorganisms10122442)
Supplement: Supplementary file 1 [file microorganisms-10-02442-s001.zip › microorganisms-2047039-File S1.zip!/list-283-diff-express-genes-PANTHER-category-v02.html]

this frame prevents back forward cache


The mission of the PANTHER knowledgebase is to support biomedical and other research by providing **comprehensive information about the evolution of protein-coding gene families**, particularly protein phylogeny, function and genetic variation impacting that function. More...

|  |  |  |
| --- | --- | --- |
|  | All Genes and orthologs Pathways PANTHER families Ontology terms | Go |

|  |  |  |  |  |  |  |  |  |  |  |  |  |  |  |  |  |  |
| --- | --- | --- | --- | --- | --- | --- | --- | --- | --- | --- | --- | --- | --- | --- | --- | --- | --- |
|  | Home |  | About |  | Data Version |  | Tools |  | API/Services |  | Publications |  | Workspace |  | Downloads |  | FAQ/Help/Tutorial |

|  |  |  |  |  |  |
| --- | --- | --- | --- | --- | --- |
|  | Login |  | Register |  | Contact us |

|  |  |  |
| --- | --- | --- |
|  | **PANTHER17.0 Released.** | Current Release: **PANTHER 17.0**  \|  **15,619** family phylogenetic trees  \|  **143** species  \|  News   Whole genome function views |


PANTHER Ontology Terms list

|  |  |
| --- | --- |
| Convert List to:  -Select-  Genes PANTHER Families | Send list to:  -Select- Workspace File Text |

|  |  |  |
| --- | --- | --- |
| Display:  1020305010020050010001000020000 items per page |  | Refine Search |

|  |  |  |
| --- | --- | --- |
| Hits 1-170 of 170 | [ page: (1) ] | Number of mapped ids found 121 IDs not found (116) |

|  |  |  |  |  |  |  |  |
| --- | --- | --- | --- | --- | --- | --- | --- |
| clr all | | Category ID | **Mapped IDs** | Name | Parent | Child | Families and Subfamilies |
|  | **1.** | GO:0016747 | HALSA\|EnsemblGenome=VNG\_0654C\|UniProtKB=Q9HRK6 | acyltransferase activity, transferring groups other than amino-acyl groups | acyltransferase activity | palmitoyltransferase activity acetyltransferase activity O-acyltransferase activity N-acyltransferase activity | 1243 |
|  | **2.** | GO:0006099 | HALSA\|EnsemblGenome=VNG\_2102G\|UniProtKB=Q9HNH4 | tricarboxylic acid cycle | aerobic respiration | - | 110 |
|  | **3.** | GO:1901607 | HALSA\|EnsemblGenome=VNG\_1815G\|UniProtKB=Q9HP42 | alpha-amino acid biosynthetic process | alpha-amino acid metabolic process | leucine biosynthetic process aspartate family amino acid biosynthetic process | 450 |
|  | **4.** | GO:0009064 | HALSA\|EnsemblGenome=VNG\_1815G\|UniProtKB=Q9HP42 HALSA\|EnsemblGenome=VNG\_1814G\|UniProtKB=Q9HP43 | glutamine family amino acid metabolic process | alpha-amino acid metabolic process | glutamate metabolic process arginine catabolic process | 190 |
|  | **5.** | GO:0009066 | HALSA\|EnsemblGenome=VNG\_1882G\|UniProtKB=Q9HNZ1 | aspartate family amino acid metabolic process | alpha-amino acid metabolic process | aspartate family amino acid biosynthetic process | 164 |
|  | **6.** | GO:0015179 | HALSA\|EnsemblGenome=VNG\_1240G\|UniProtKB=Q9HQB3 | L-amino acid transmembrane transporter activity | amino acid transmembrane transporter activity | L-glutamate transmembrane transporter activity L-lysine transmembrane transporter activity | 171 |
|  | **7.** | GO:0016887 | HALSA\|EnsemblGenome=VNG\_1462G\|UniProtKB=Q9HPV0 HALSA\|EnsemblGenome=VNG\_6264G\|UniProtKB=Q9HHR7 | ATP hydrolysis activity | ATP-dependent activity | - | 1719 |
|  | **8.** | GO:0051536 | HALSA\|EnsemblGenome=VNG\_1882G\|UniProtKB=Q9HNZ1 | iron-sulfur cluster binding | binding | - | 172 |
|  | **9.** | GO:0044283 | HALSA\|EnsemblGenome=VNG\_1769C\|UniProtKB=Q9HP77 HALSA\|EnsemblGenome=VNG\_6303G\|UniProtKB=Q9HHN8 | small molecule biosynthetic process | biosynthetic process | alcohol biosynthetic process hexose biosynthetic process ubiquinone biosynthetic process organic acid biosynthetic process | 1201 |
|  | **10.** | GO:0044249 | HALSA\|EnsemblGenome=VNG\_1781C\|UniProtKB=Q9HP67 | cellular biosynthetic process | biosynthetic process | heterocycle biosynthetic process cellular nitrogen compound biosynthetic process ubiquinone biosynthetic process organic acid biosynthetic process cellular macromolecule biosynthetic process ergosterol biosynthetic process aromatic compound biosynthetic process phospholipid biosynthetic process cellular carbohydrate biosynthetic process carotenoid biosynthetic process membrane lipid biosynthetic process glycerolipid biosynthetic process | 11234 |
|  | **11.** | GO:1901137 | HALSA\|EnsemblGenome=VNG\_6303G\|UniProtKB=Q9HHN8 | carbohydrate derivative biosynthetic process | carbohydrate derivative metabolic process | lipopolysaccharide biosynthetic process ribose phosphate biosynthetic process glycolipid biosynthetic process glycoprotein biosynthetic process aminoglycan biosynthetic process | 1217 |
|  | **12.** | GO:0016836 | HALSA\|EnsemblGenome=VNG\_1208G\|UniProtKB=Q9HQD8 | hydro-lyase activity | carbon-oxygen lyase activity | - | 353 |
|  | **13.** | GO:0032787 | HALSA\|EnsemblGenome=VNG\_1114G\|UniProtKB=Q9HQK8 | monocarboxylic acid metabolic process | carboxylic acid metabolic process | monocarboxylic acid catabolic process fatty acid metabolic process glycolytic process monocarboxylic acid biosynthetic process | 1045 |
|  | **14.** | GO:0003333 | HALSA\|EnsemblGenome=VNG\_1240G\|UniProtKB=Q9HQB3 | amino acid transmembrane transport | carboxylic acid transmembrane transport | basic amino acid transmembrane transport L-alpha-amino acid transmembrane transport | 234 |
|  | **15.** | GO:1901575 | HALSA\|EnsemblGenome=VNG\_1114G\|UniProtKB=Q9HQK8 | organic substance catabolic process | catabolic process | lipid catabolic process organonitrogen compound catabolic process organophosphate catabolic process macromolecule catabolic process organic acid catabolic process carbohydrate derivative catabolic process organic cyclic compound catabolic process carbohydrate catabolic process | 4004 |
|  | **16.** | GO:0044282 | HALSA\|EnsemblGenome=VNG\_1114G\|UniProtKB=Q9HQK8 | small molecule catabolic process | catabolic process | organic acid catabolic process nucleobase-containing small molecule catabolic process | 848 |
|  | **17.** | GO:0019104 | HALSA\|EnsemblGenome=VNG\_0592G\|UniProtKB=Q9HRQ5 | DNA N-glycosylase activity | catalytic activity, acting on DNA | - | 42 |
|  | **18.** | GO:0004540 | HALSA\|EnsemblGenome=VNG\_2493C\|UniProtKB=Q9HML2 | ribonuclease activity | catalytic activity, acting on RNA | endoribonuclease activity 3'-5'-exoribonuclease activity | 301 |
|  | **19.** | GO:0016829 | HALSA\|EnsemblGenome=VNG\_1114G\|UniProtKB=Q9HQK8 HALSA\|EnsemblGenome=VNG\_1212G\|UniProtKB=Q9HQD5 | lyase activity | catalytic activity | phosphorus-oxygen lyase activity carbon-carbon lyase activity carbon-oxygen lyase activity | 882 |
|  | **20.** | GO:0016491 | HALSA\|EnsemblGenome=VNG\_0281G\|UniProtKB=Q9HSD9 HALSA\|EnsemblGenome=VNG\_1644G\|UniProtKB=Q9HPG8 HALSA\|EnsemblGenome=VNG\_0935G\|UniProtKB=Q9HQZ4 HALSA\|EnsemblGenome=VNG\_0628G\|UniProtKB=Q9HRM7 | oxidoreductase activity | catalytic activity | electron transfer activity oxidoreductase activity, acting on single donors with incorporation of molecular oxygen, incorporation of two atoms of oxygen oxidoreductase activity, acting on the CH-CH group of donors oxidoreductase activity, acting on metal ions oxidoreductase activity, acting on NAD(P)H oxidoreductase activity, acting on the CH-OH group of donors, NAD or NADP as acceptor oxidoreductase activity, acting on a sulfur group of donors monooxygenase activity | 4203 |
|  | **21.** | GO:0016787 | HALSA\|EnsemblGenome=VNG\_2186G\|UniProtKB=Q9HNA0 | hydrolase activity | catalytic activity | peptidase activity serine hydrolase activity hydrolase activity, acting on glycosyl bonds hydrolase activity, acting on ester bonds deaminase activity hydrolase activity, acting on acid anhydrides deacetylase activity ether hydrolase activity hydrolase activity, acting on carbon-nitrogen (but not peptide) bonds | 9060 |
|  | **22.** | GO:0016874 | HALSA\|EnsemblGenome=VNG\_0391C\|UniProtKB=Q9HS60 HALSA\|EnsemblGenome=VNG\_1939G\|UniProtKB=Q9HNU7 HALSA\|EnsemblGenome=VNG\_0623G\|UniProtKB=Q9HRN1 | ligase activity | catalytic activity | ligase activity, forming carbon-sulfur bonds | 732 |
|  | **23.** | GO:0005886 | HALSA\|EnsemblGenome=VNG\_1455H\|UniProtKB=Q9HPV6 HALSA\|EnsemblGenome=VNG\_2413H\|UniProtKB=Q9HMS1 HALSA\|EnsemblGenome=VNG\_0831G\|UniProtKB=Q9HR72 | plasma membrane | cell periphery | intrinsic component of plasma membrane external side of plasma membrane plasma membrane region leading edge membrane plasma membrane protein complex extrinsic component of plasma membrane sarcolemma cytoplasmic side of plasma membrane | 15135 |
|  | **24.** | GO:0009897 | HALSA\|EnsemblGenome=VNG\_2589C\|UniProtKB=Q9HMD9 | external side of plasma membrane | cell surface | - | 397 |
|  | **25.** | GO:1901605 | HALSA\|EnsemblGenome=VNG\_0623G\|UniProtKB=Q9HRN1 | alpha-amino acid metabolic process | cellular amino acid metabolic process | aspartate family amino acid metabolic process glutamine family amino acid metabolic process tyrosine metabolic process alpha-amino acid biosynthetic process | 733 |
|  | **26.** | GO:0009081 | HALSA\|EnsemblGenome=VNG\_0623G\|UniProtKB=Q9HRN1 | branched-chain amino acid metabolic process | cellular amino acid metabolic process | branched-chain amino acid biosynthetic process | 126 |
|  | **27.** | GO:0032153 | HALSA\|EnsemblGenome=VNG\_0265G\|UniProtKB=Q9HSE9 HALSA\|EnsemblGenome=VNG\_0192G\|UniProtKB=P0DMR7 | cell division site | cellular anatomical entity | actomyosin contractile ring | 112 |
|  | **28.** | GO:0005622 | HALSA\|EnsemblGenome=VNG\_0933G\|UniProtKB=Q9HQZ6 | intracellular anatomical structure | cellular anatomical entity | intracellular organelle DNA helicase complex proteasome accessory complex thylakoid cytoplasm proton-transporting ATP synthase complex, coupling factor F(o) exosome (RNase complex) methyltransferase complex | 33185 |
|  | **29.** | GO:0019438 | HALSA\|EnsemblGenome=VNG\_1230G\|UniProtKB=Q9HQC2 | aromatic compound biosynthetic process | cellular aromatic compound metabolic process | nucleobase-containing compound biosynthetic process purine-containing compound biosynthetic process heme biosynthetic process | 7556 |
|  | **30.** | GO:0019439 | HALSA\|EnsemblGenome=VNG\_0437C\|UniProtKB=Q9HS25 | aromatic compound catabolic process | cellular aromatic compound metabolic process | nucleobase-containing compound catabolic process purine-containing compound catabolic process aromatic amino acid family catabolic process | 724 |
|  | **31.** | GO:0044271 | HALSA\|EnsemblGenome=VNG\_1769C\|UniProtKB=Q9HP77 | cellular nitrogen compound biosynthetic process | cellular biosynthetic process | nucleobase-containing compound biosynthetic process amide biosynthetic process purine-containing compound biosynthetic process heme biosynthetic process polyamine biosynthetic process | 8752 |
|  | **32.** | GO:0044270 | HALSA\|EnsemblGenome=VNG\_0437C\|UniProtKB=Q9HS25 | cellular nitrogen compound catabolic process | cellular catabolic process | nucleobase-containing compound catabolic process purine-containing compound catabolic process pyrimidine nucleobase catabolic process | 702 |
|  | **33.** | GO:0044248 | HALSA\|EnsemblGenome=VNG\_1114G\|UniProtKB=Q9HQK8 | cellular catabolic process | cellular metabolic process | cellular nitrogen compound catabolic process aromatic compound catabolic process cellular macromolecule catabolic process cellular carbohydrate catabolic process organic acid catabolic process cellular lipid catabolic process sulfur compound catabolic process autophagy heterocycle catabolic process | 3824 |
|  | **34.** | GO:0044255 | HALSA\|EnsemblGenome=VNG\_1781C\|UniProtKB=Q9HP67 | cellular lipid metabolic process | cellular metabolic process | neutral lipid metabolic process glycerolipid metabolic process ergosterol metabolic process membrane lipid metabolic process lipid modification lipopolysaccharide biosynthetic process cellular lipid catabolic process phospholipid metabolic process fatty acid metabolic process carotenoid biosynthetic process | 1895 |
|  | **35.** | GO:0042180 | HALSA\|EnsemblGenome=VNG\_1114G\|UniProtKB=Q9HQK8 | cellular ketone metabolic process | cellular metabolic process | quinone metabolic process | 183 |
|  | **36.** | GO:0043604 | HALSA\|EnsemblGenome=VNG\_0997G\|UniProtKB=Q9HQU8 | amide biosynthetic process | cellular nitrogen compound biosynthetic process | peptide biosynthetic process ceramide biosynthetic process | 1468 |
|  | **37.** | GO:0043603 | HALSA\|EnsemblGenome=VNG\_0437C\|UniProtKB=Q9HS25 | cellular amide metabolic process | cellular nitrogen compound metabolic process | amide biosynthetic process peptide metabolic process acyl-CoA metabolic process ceramide metabolic process | 2107 |
|  | **38.** | GO:0051301 | HALSA\|EnsemblGenome=VNG\_0265G\|UniProtKB=Q9HSE9 HALSA\|EnsemblGenome=VNG\_0192G\|UniProtKB=P0DMR7 | cell division | cellular process | cytokinesis | 302 |
|  | **39.** | GO:0019725 | HALSA\|EnsemblGenome=VNG\_2115H\|UniProtKB=Q9HNG5 | cellular homeostasis | cellular process | cellular chemical homeostasis | 867 |
|  | **40.** | GO:0006457 | HALSA\|EnsemblGenome=VNG\_6201G\|UniProtKB=Q9HHW1 | protein folding | cellular process | 'de novo' protein folding chaperone-mediated protein folding | 692 |
|  | **41.** | GO:0018193 | HALSA\|EnsemblGenome=VNG\_0391C\|UniProtKB=Q9HS60 | peptidyl-amino acid modification | cellular protein modification process | peptidyl-lysine modification peptidyl-threonine phosphorylation peptidyl-tyrosine modification protein N-linked glycosylation via asparagine peptidyl-arginine modification peptidyl-serine modification | 1337 |
|  | **42.** | GO:0006974 | HALSA\|EnsemblGenome=VNG\_2589C\|UniProtKB=Q9HMD9 | cellular response to DNA damage stimulus | cellular response to stress | intrinsic apoptotic signaling pathway in response to DNA damage signal transduction in response to DNA damage DNA repair | 807 |
|  | **43.** | GO:0005829 | HALSA\|EnsemblGenome=VNG\_1822G\|UniProtKB=Q9HP37 HALSA\|EnsemblGenome=VNG\_6316G\|UniProtKB=Q48295 HALSA\|EnsemblGenome=VNG\_0930G\|UniProtKB=Q9HQZ9 HALSA\|EnsemblGenome=VNG\_1230G\|UniProtKB=Q9HQC2 HALSA\|EnsemblGenome=VNG\_1285G\|UniProtKB=Q9HQ80 | cytosol | cytoplasm | inflammasome complex cytosolic ribosome chaperonin-containing T-complex | 4852 |
|  | **44.** | GO:0043565 | HALSA\|EnsemblGenome=VNG\_1285G\|UniProtKB=Q9HQ80 | sequence-specific DNA binding | DNA binding | telomeric DNA binding sequence-specific double-stranded DNA binding | 4816 |
|  | **45.** | PC00116 | HALSA\|EnsemblGenome=VNG\_1886C\|UniProtKB=Q9HNY8 HALSA\|EnsemblGenome=VNG\_1285G\|UniProtKB=Q9HQ80 | helix-turn-helix transcription factor | DNA-binding transcription factor | Trp repressor-like transcription factor winged helix/forkhead transcription factor homeodomain transcription factor paired box transcription factor Lambda repressor-like transcription factor | 1700 |
|  | **46.** | PC00199 | HALSA\|EnsemblGenome=VNG\_1224G\|UniProtKB=Q9HQC7 | replication origin binding protein | DNA metabolism protein | - | 24 |
|  | **47.** | PC00014 | HALSA\|EnsemblGenome=VNG\_1754G\|UniProtKB=Q9HP89 | DNA photolyase | DNA metabolism protein | - | 19 |
|  | **48.** | GO:0006284 | HALSA\|EnsemblGenome=VNG\_0592G\|UniProtKB=Q9HRQ5 | base-excision repair | DNA repair | - | 60 |
|  | **49.** | GO:0015986 | HALSA\|EnsemblGenome=VNG\_6055G\|UniProtKB=O54596 | ATP synthesis coupled proton transport | energy coupled proton transport, down electrochemical gradient | - | 303 |
|  | **50.** | GO:0016895 | HALSA\|EnsemblGenome=VNG\_2493C\|UniProtKB=Q9HML2 | exodeoxyribonuclease activity, producing 5'-phosphomonoesters | exonuclease activity, active with either ribo- or deoxyribonucleic acids and producing 5'-phosphomonoesters | 3'-5'-exodeoxyribonuclease activity | 48 |
|  | **51.** | GO:0008409 | HALSA\|EnsemblGenome=VNG\_2493C\|UniProtKB=Q9HML2 | 5'-3' exonuclease activity | exonuclease activity | - | 47 |
|  | **52.** | PC00218 | HALSA\|EnsemblGenome=VNG\_0996G\|UniProtKB=Q9HQU9 HALSA\|EnsemblGenome=VNG\_0794G\|UniProtKB=Q9HRA0 HALSA\|EnsemblGenome=VNG\_0852C\|UniProtKB=Q9HR58 HALSA\|EnsemblGenome=VNG\_1464G\|UniProtKB=Q9HPU8 HALSA\|EnsemblGenome=VNG\_0826C\|UniProtKB=Q9HR76 | DNA-binding transcription factor | gene-specific transcriptional regulator | helix-turn-helix transcription factor MADS box transcription factor HMG box transcription factor zinc finger transcription factor basic helix-loop-helix transcription factor basic leucine zipper transcription factor TATA-binding transcription factor immunoglobulin fold transcription factor SMAD-like transcription factor | 4655 |
|  | **53.** | GO:0006536 | HALSA\|EnsemblGenome=VNG\_0628G\|UniProtKB=Q9HRM7 | glutamate metabolic process | glutamine family amino acid metabolic process | - | 70 |
|  | **54.** | GO:0005525 | HALSA\|EnsemblGenome=VNG\_0265G\|UniProtKB=Q9HSE9 HALSA\|EnsemblGenome=VNG\_0192G\|UniProtKB=P0DMR7 | GTP binding | guanyl ribonucleotide binding | - | 663 |
|  | **55.** | PC00246 | HALSA\|EnsemblGenome=VNG\_0536G\|UniProtKB=Q9HRU8 | winged helix/forkhead transcription factor | helix-turn-helix transcription factor | - | 552 |
|  | **56.** | GO:0046700 | HALSA\|EnsemblGenome=VNG\_0437C\|UniProtKB=Q9HS25 | heterocycle catabolic process | heterocycle metabolic process | nucleobase-containing compound catabolic process purine-containing compound catabolic process pyrimidine nucleobase catabolic process | 672 |
|  | **57.** | GO:0018130 | HALSA\|EnsemblGenome=VNG\_1769C\|UniProtKB=Q9HP77 | heterocycle biosynthetic process | heterocycle metabolic process | nucleobase-containing compound biosynthetic process purine-containing compound biosynthetic process heme biosynthetic process | 7359 |
|  | **58.** | GO:0016811 | HALSA\|EnsemblGenome=VNG\_1769C\|UniProtKB=Q9HP77 HALSA\|EnsemblGenome=VNG\_0437C\|UniProtKB=Q9HS25 | hydrolase activity, acting on carbon-nitrogen (but not peptide) bonds, in linear amides | hydrolase activity, acting on carbon-nitrogen (but not peptide) bonds | histone deacetylase activity | 139 |
|  | **59.** | GO:0016790 | HALSA\|EnsemblGenome=VNG\_1407C\|UniProtKB=Q9HPZ1 | thiolester hydrolase activity | hydrolase activity, acting on ester bonds | - | 203 |
|  | **60.** | PC00185 | HALSA\|EnsemblGenome=VNG\_1450G\|UniProtKB=Q9HPW1 | phosphodiesterase | hydrolase | - | 215 |
|  | **61.** | PC00097 | HALSA\|EnsemblGenome=VNG\_1407C\|UniProtKB=Q9HPZ1 | esterase | hydrolase | - | 547 |
|  | **62.** | GO:0006188 | HALSA\|EnsemblGenome=VNG\_1939G\|UniProtKB=Q9HNU7 | IMP biosynthetic process | IMP metabolic process | - | 31 |
|  | **63.** | GO:0098662 | HALSA\|EnsemblGenome=VNG\_1632G\|UniProtKB=Q9HPH6 | inorganic cation transmembrane transport | inorganic ion transmembrane transport | calcium ion transmembrane transport proton transmembrane transport iron ion transmembrane transport sodium ion transmembrane transport potassium ion transmembrane transport | 1215 |
|  | **64.** | GO:1902495 | HALSA\|EnsemblGenome=VNG\_6264G\|UniProtKB=Q9HHR7 HALSA\|EnsemblGenome=VNG\_1632G\|UniProtKB=Q9HPH6 | transmembrane transporter complex | integral component of membrane | respiratory chain complex I ion channel complex mitochondrial respiratory chain complex III | 486 |
|  | **65.** | GO:0005887 | HALSA\|EnsemblGenome=VNG\_1234C\|UniProtKB=Q9HQB9 HALSA\|EnsemblGenome=VNG\_2589C\|UniProtKB=Q9HMD9 | integral component of plasma membrane | integral component of membrane | integral component of synaptic membrane | 2466 |
|  | **66.** | GO:0005737 | HALSA\|EnsemblGenome=VNG\_2186G\|UniProtKB=Q9HNA0 HALSA\|EnsemblGenome=VNG\_0281G\|UniProtKB=Q9HSD9 HALSA\|EnsemblGenome=VNG\_1114G\|UniProtKB=Q9HQK8 HALSA\|EnsemblGenome=VNG\_0265G\|UniProtKB=Q9HSE9 HALSA\|EnsemblGenome=VNG\_2475C\|UniProtKB=Q9HMM2 HALSA\|EnsemblGenome=VNG\_0437C\|UniProtKB=Q9HS25 HALSA\|EnsemblGenome=VNG\_1815G\|UniProtKB=Q9HP42 HALSA\|EnsemblGenome=VNG\_1814G\|UniProtKB=Q9HP43 HALSA\|EnsemblGenome=VNG\_0192G\|UniProtKB=P0DMR7 HALSA\|EnsemblGenome=VNG\_1939G\|UniProtKB=Q9HNU7 HALSA\|EnsemblGenome=VNG\_1404G\|UniProtKB=Q9HPZ3 | cytoplasm | intracellular anatomical structure | SMN complex endoplasmic reticulum cell cortex cytoplasmic vesicle cytoplasmic microtubule cytoplasmic region sarcoplasmic reticulum cytosol cytoplasmic ribonucleoprotein granule mitochondrion SMN-Sm protein complex microbody endoplasmic reticulum-Golgi intermediate compartment contractile fiber perinuclear region of cytoplasm vacuole membrane coat Golgi apparatus phagophore assembly site SNARE complex plastid eukaryotic translation initiation factor 3 complex | 24049 |
|  | **67.** | GO:0043168 | HALSA\|EnsemblGenome=VNG\_1230G\|UniProtKB=Q9HQC2 | anion binding | ion binding | GDP binding carboxylic acid binding flavin adenine dinucleotide binding phosphatidylinositol-4,5-bisphosphate binding purine ribonucleoside triphosphate binding | 1926 |
|  | **68.** | PC00160 | HALSA\|EnsemblGenome=VNG\_1567G\|UniProtKB=Q9HPM1 HALSA\|EnsemblGenome=VNG\_0653G\|UniProtKB=Q9HRK7 | mutase | isomerase | - | 132 |
|  | **69.** | PC00172 | HALSA\|EnsemblGenome=VNG\_1900C\|UniProtKB=Q9HNX7 | nucleotide kinase | kinase | - | 248 |
|  | **70.** | GO:0016877 | HALSA\|EnsemblGenome=VNG\_0997G\|UniProtKB=Q9HQU8 | ligase activity, forming carbon-sulfur bonds | ligase activity | long-chain fatty acid-CoA ligase activity | 240 |
|  | **71.** | GO:0016835 | HALSA\|EnsemblGenome=VNG\_1230G\|UniProtKB=Q9HQC2 | carbon-oxygen lyase activity | lyase activity | hydro-lyase activity | 383 |
|  | **72.** | PC00079 | HALSA\|EnsemblGenome=VNG\_1458G\|UniProtKB=Q9HPV3 HALSA\|EnsemblGenome=VNG\_2137G\|UniProtKB=Q9HNE5 | cyclase | lyase | adenylate cyclase guanylate cyclase | 149 |
|  | **73.** | PC00120 | HALSA\|EnsemblGenome=VNG\_1208G\|UniProtKB=Q9HQD8 | hydratase | lyase | - | 68 |
|  | **74.** | GO:0097478 | HALSA\|EnsemblGenome=VNG\_2589C\|UniProtKB=Q9HMD9 | leaflet of membrane bilayer | membrane | - | 626 |
|  | **75.** | GO:0006739 | HALSA\|EnsemblGenome=VNG\_1900C\|UniProtKB=Q9HNX7 | NADP metabolic process | metabolic process | - | 91 |
|  | **76.** | PC00144 | HALSA\|EnsemblGenome=VNG\_1114G\|UniProtKB=Q9HQK8 HALSA\|EnsemblGenome=VNG\_2471G\|UniProtKB=Q9HMM6 HALSA\|EnsemblGenome=VNG\_0627G\|UniProtKB=Q9HRM8 HALSA\|EnsemblGenome=VNG\_1230G\|UniProtKB=Q9HQC2 HALSA\|EnsemblGenome=VNG\_1212G\|UniProtKB=Q9HQD5 | lyase | metabolite interconversion enzyme | dehydratase hydratase aldolase cyclase decarboxylase | 1408 |
|  | **77.** | PC00142 | HALSA\|EnsemblGenome=VNG\_1565G\|UniProtKB=Q9HPM3 HALSA\|EnsemblGenome=VNG\_1305G\|UniProtKB=Q9HQ67 HALSA\|EnsemblGenome=VNG\_6303G\|UniProtKB=Q9HHN8 HALSA\|EnsemblGenome=VNG\_1815G\|UniProtKB=Q9HP42 HALSA\|EnsemblGenome=VNG\_1814G\|UniProtKB=Q9HP43 HALSA\|EnsemblGenome=VNG\_1939G\|UniProtKB=Q9HNU7 HALSA\|EnsemblGenome=VNG\_0623G\|UniProtKB=Q9HRN1 HALSA\|EnsemblGenome=VNG\_0997G\|UniProtKB=Q9HQU8 | ligase | metabolite interconversion enzyme | - | 515 |
|  | **78.** | PC00176 | HALSA\|EnsemblGenome=VNG\_1972G\|UniProtKB=Q9HNS2 HALSA\|EnsemblGenome=VNG\_1455H\|UniProtKB=Q9HPV6 HALSA\|EnsemblGenome=VNG\_6055G\|UniProtKB=O54596 HALSA\|EnsemblGenome=VNG\_0830G\|UniProtKB=Q9HR73 HALSA\|EnsemblGenome=VNG\_2413H\|UniProtKB=Q9HMS1 HALSA\|EnsemblGenome=VNG\_2195G\|UniProtKB=Q9HN94 HALSA\|EnsemblGenome=VNG\_0933G\|UniProtKB=Q9HQZ6 HALSA\|EnsemblGenome=VNG\_2115H\|UniProtKB=Q9HNG5 | oxidoreductase | metabolite interconversion enzyme | oxygenase hydroxylase peroxidase reductase dehydrogenase oxidase | 5481 |
|  | **79.** | PC00121 | HALSA\|EnsemblGenome=VNG\_1769C\|UniProtKB=Q9HP77 HALSA\|EnsemblGenome=VNG\_0231C\|UniProtKB=Q9HSH2 HALSA\|EnsemblGenome=VNG\_6123G\|UniProtKB=O51991 | hydrolase | metabolite interconversion enzyme | esterase pyrophosphatase deaminase glycosidase deacetylase glucosidase phosphatase phosphodiesterase amylase lipase galactosidase | 3716 |
|  | **80.** | PC00220 | HALSA\|EnsemblGenome=VNG\_2394G\|UniProtKB=Q9HMT6 HALSA\|EnsemblGenome=VNG\_1882G\|UniProtKB=Q9HNZ1 HALSA\|EnsemblGenome=VNG\_1574G\|UniProtKB=Q9HPL6 HALSA\|EnsemblGenome=VNG\_2102G\|UniProtKB=Q9HNH4 | transferase | metabolite interconversion enzyme | nucleotidyltransferase methyltransferase acetyltransferase transketolase glycosyltransferase acyltransferase transaldolase transaminase kinase | 3275 |
|  | **81.** | GO:0000287 | HALSA\|EnsemblGenome=VNG\_0627G\|UniProtKB=Q9HRM8 | magnesium ion binding | metal ion binding | - | 76 |
|  | **82.** | GO:0000041 | HALSA\|EnsemblGenome=VNG\_1632G\|UniProtKB=Q9HPH6 | transition metal ion transport | metal ion transport | iron ion transport | 228 |
|  | **83.** | GO:0008757 | HALSA\|EnsemblGenome=VNG\_1980C\|UniProtKB=Q9HNR6 | S-adenosylmethionine-dependent methyltransferase activity | methyltransferase activity | lysine N-methyltransferase activity rRNA (adenine) methyltransferase activity | 566 |
|  | **84.** | GO:0000086 | HALSA\|EnsemblGenome=VNG\_1224G\|UniProtKB=Q9HQC7 | G2/M transition of mitotic cell cycle | mitotic cell cycle phase transition | - | 78 |
|  | **85.** | GO:0140014 | HALSA\|EnsemblGenome=VNG\_1224G\|UniProtKB=Q9HQC7 | mitotic nuclear division | mitotic cell cycle process | mitotic sister chromatid segregation mitotic spindle assembly | 1092 |
|  | **86.** | GO:0033314 | HALSA\|EnsemblGenome=VNG\_1224G\|UniProtKB=Q9HQC7 | mitotic DNA replication checkpoint signaling | mitotic G2/M transition checkpoint | - | 21 |
|  | **87.** | GO:0060089 | HALSA\|EnsemblGenome=VNG\_1234C\|UniProtKB=Q9HQB9 | molecular transducer activity | molecular\_function | signaling receptor activity | 2919 |
|  | **88.** | GO:0003677 | HALSA\|EnsemblGenome=VNG\_1754G\|UniProtKB=Q9HP89 | DNA binding | nucleic acid binding | single-stranded DNA binding damaged DNA binding sequence-specific DNA binding chromatin DNA binding double-stranded DNA binding DNA binding, bending four-way junction DNA binding | 5782 |
|  | **89.** | GO:0009119 | HALSA\|EnsemblGenome=VNG\_6303G\|UniProtKB=Q9HHN8 | ribonucleoside metabolic process | nucleoside metabolic process | purine ribonucleoside metabolic process | 43 |
|  | **90.** | GO:0003924 | HALSA\|EnsemblGenome=VNG\_0265G\|UniProtKB=Q9HSE9 HALSA\|EnsemblGenome=VNG\_0192G\|UniProtKB=P0DMR7 | GTPase activity | nucleoside-triphosphatase activity | - | 1252 |
|  | **91.** | GO:0032553 | HALSA\|EnsemblGenome=VNG\_1230G\|UniProtKB=Q9HQC2 | ribonucleotide binding | nucleotide binding | purine ribonucleotide binding | 1077 |
|  | **92.** | GO:0050660 | HALSA\|EnsemblGenome=VNG\_1754G\|UniProtKB=Q9HP89 | flavin adenine dinucleotide binding | nucleotide binding | - | 248 |
|  | **93.** | GO:0009165 | HALSA\|EnsemblGenome=VNG\_1900C\|UniProtKB=Q9HNX7 HALSA\|EnsemblGenome=VNG\_1882G\|UniProtKB=Q9HNZ1 | nucleotide biosynthetic process | nucleotide metabolic process | ribonucleotide biosynthetic process pyrimidine nucleotide biosynthetic process cyclic nucleotide biosynthetic process purine nucleotide biosynthetic process | 670 |
|  | **94.** | GO:0046394 | HALSA\|EnsemblGenome=VNG\_1230G\|UniProtKB=Q9HQC2 | carboxylic acid biosynthetic process | organic acid biosynthetic process | cellular amino acid biosynthetic process icosanoid biosynthetic process monocarboxylic acid biosynthetic process | 783 |
|  | **95.** | GO:0046395 | HALSA\|EnsemblGenome=VNG\_0437C\|UniProtKB=Q9HS25 HALSA\|EnsemblGenome=VNG\_1208G\|UniProtKB=Q9HQD8 HALSA\|EnsemblGenome=VNG\_0628G\|UniProtKB=Q9HRM7 HALSA\|EnsemblGenome=VNG\_0623G\|UniProtKB=Q9HRN1 | carboxylic acid catabolic process | organic acid catabolic process | monocarboxylic acid catabolic process arginine catabolic process | 537 |
|  | **96.** | GO:0034654 | HALSA\|EnsemblGenome=VNG\_6303G\|UniProtKB=Q9HHN8 | nucleobase-containing compound biosynthetic process | organic cyclic compound biosynthetic process | nucleoside phosphate biosynthetic process DNA biosynthetic process RNA biosynthetic process | 7026 |
|  | **97.** | GO:0006760 | HALSA\|EnsemblGenome=VNG\_0437C\|UniProtKB=Q9HS25 | folic acid-containing compound metabolic process | organic cyclic compound metabolic process | - | 87 |
|  | **98.** | GO:0009072 | HALSA\|EnsemblGenome=VNG\_1230G\|UniProtKB=Q9HQC2 | aromatic amino acid family metabolic process | organic cyclic compound metabolic process | aromatic amino acid family catabolic process tyrosine metabolic process | 168 |
|  | **99.** | GO:0046165 | HALSA\|EnsemblGenome=VNG\_1781C\|UniProtKB=Q9HP67 | alcohol biosynthetic process | organic hydroxy compound biosynthetic process | secondary alcohol biosynthetic process | 201 |
|  | **100.** | GO:1901362 | HALSA\|EnsemblGenome=VNG\_1769C\|UniProtKB=Q9HP77 HALSA\|EnsemblGenome=VNG\_1230G\|UniProtKB=Q9HQC2 | organic cyclic compound biosynthetic process | organic substance biosynthetic process | steroid biosynthetic process nucleobase-containing compound biosynthetic process purine-containing compound biosynthetic process heme biosynthetic process | 7676 |
|  | **101.** | GO:1901566 | HALSA\|EnsemblGenome=VNG\_1769C\|UniProtKB=Q9HP77 HALSA\|EnsemblGenome=VNG\_1900C\|UniProtKB=Q9HNX7 HALSA\|EnsemblGenome=VNG\_6303G\|UniProtKB=Q9HHN8 HALSA\|EnsemblGenome=VNG\_1230G\|UniProtKB=Q9HQC2 HALSA\|EnsemblGenome=VNG\_1882G\|UniProtKB=Q9HNZ1 | organonitrogen compound biosynthetic process | organic substance biosynthetic process | cellular amino acid biosynthetic process glycoprotein biosynthetic process aminoglycan biosynthetic process sphingolipid biosynthetic process peptide biosynthetic process purine-containing compound biosynthetic process branched-chain amino acid biosynthetic process heme biosynthetic process polyamine biosynthetic process pyrimidine nucleotide biosynthetic process lipoprotein biosynthetic process phosphatidylcholine biosynthetic process | 3692 |
|  | **102.** | GO:0008610 | HALSA\|EnsemblGenome=VNG\_1781C\|UniProtKB=Q9HP67 | lipid biosynthetic process | organic substance biosynthetic process | lipopolysaccharide biosynthetic process phospholipid biosynthetic process steroid biosynthetic process fatty acid biosynthetic process carotenoid biosynthetic process membrane lipid biosynthetic process glycerolipid biosynthetic process | 1137 |
|  | **103.** | GO:1901361 | HALSA\|EnsemblGenome=VNG\_0437C\|UniProtKB=Q9HS25 | organic cyclic compound catabolic process | organic substance catabolic process | steroid catabolic process nucleobase-containing compound catabolic process purine-containing compound catabolic process aromatic amino acid family catabolic process pyrimidine nucleobase catabolic process | 792 |
|  | **104.** | GO:1901565 | HALSA\|EnsemblGenome=VNG\_0437C\|UniProtKB=Q9HS25 HALSA\|EnsemblGenome=VNG\_1208G\|UniProtKB=Q9HQD8 HALSA\|EnsemblGenome=VNG\_0628G\|UniProtKB=Q9HRM7 HALSA\|EnsemblGenome=VNG\_0623G\|UniProtKB=Q9HRN1 | organonitrogen compound catabolic process | organic substance catabolic process | purine-containing compound catabolic process nucleoside catabolic process aromatic amino acid family catabolic process protein catabolic process arginine catabolic process sphingolipid catabolic process pyrimidine nucleobase catabolic process | 2509 |
|  | **105.** | GO:1901615 | HALSA\|EnsemblGenome=VNG\_1114G\|UniProtKB=Q9HQK8 | organic hydroxy compound metabolic process | organic substance metabolic process | organic hydroxy compound biosynthetic process phytosteroid metabolic process sterol metabolic process alcohol metabolic process catecholamine metabolic process | 810 |
|  | **106.** | GO:0006527 | HALSA\|EnsemblGenome=VNG\_6316G\|UniProtKB=Q48295 | arginine catabolic process | organonitrogen compound catabolic process | - | 12 |
|  | **107.** | GO:0006520 | HALSA\|EnsemblGenome=VNG\_1208G\|UniProtKB=Q9HQD8 | cellular amino acid metabolic process | organonitrogen compound metabolic process | aromatic amino acid family metabolic process cellular amino acid biosynthetic process branched-chain amino acid metabolic process alpha-amino acid metabolic process | 1114 |
|  | **108.** | GO:0019646 | HALSA\|EnsemblGenome=VNG\_6055G\|UniProtKB=O54596 | aerobic electron transport chain | oxidative phosphorylation | mitochondrial electron transport, NADH to ubiquinone mitochondrial electron transport, cytochrome c to oxygen mitochondrial electron transport, succinate to ubiquinone mitochondrial electron transport, ubiquinol to cytochrome c | 145 |
|  | **109.** | GO:0003995 | HALSA\|EnsemblGenome=VNG\_0775G\|UniProtKB=Q9HRB3 HALSA\|EnsemblGenome=VNG\_1191G\|UniProtKB=Q9HQF0 | acyl-CoA dehydrogenase activity | oxidoreductase activity, acting on the CH-CH group of donors | - | 26 |
|  | **110.** | GO:0016616 | HALSA\|EnsemblGenome=VNG\_2171G\|UniProtKB=Q9HNB5 | oxidoreductase activity, acting on the CH-OH group of donors, NAD or NADP as acceptor | oxidoreductase activity | - | 816 |
|  | **111.** | GO:0016667 | HALSA\|EnsemblGenome=VNG\_1404G\|UniProtKB=Q9HPZ3 | oxidoreductase activity, acting on a sulfur group of donors | oxidoreductase activity | disulfide oxidoreductase activity | 170 |
|  | **112.** | GO:0009055 | HALSA\|EnsemblGenome=VNG\_6055G\|UniProtKB=O54596 HALSA\|EnsemblGenome=VNG\_2115H\|UniProtKB=Q9HNG5 | electron transfer activity | oxidoreductase activity | - | 124 |
|  | **113.** | PC00177 | HALSA\|EnsemblGenome=VNG\_0930G\|UniProtKB=Q9HQZ9 | oxygenase | oxidoreductase | - | 2038 |
|  | **114.** | PC00175 | HALSA\|EnsemblGenome=VNG\_2193G\|UniProtKB=Q9HN95 | oxidase | oxidoreductase | - | 785 |
|  | **115.** | PC00092 | HALSA\|EnsemblGenome=VNG\_0775G\|UniProtKB=Q9HRB3 HALSA\|EnsemblGenome=VNG\_0281G\|UniProtKB=Q9HSD9 HALSA\|EnsemblGenome=VNG\_1969G\|UniProtKB=Q9HNS4 HALSA\|EnsemblGenome=VNG\_0406C\|UniProtKB=Q9HS49 HALSA\|EnsemblGenome=VNG\_1971G\|UniProtKB=Q9HNS3 HALSA\|EnsemblGenome=VNG\_1191G\|UniProtKB=Q9HQF0 HALSA\|EnsemblGenome=VNG\_1755G\|UniProtKB=Q9HP88 HALSA\|EnsemblGenome=VNG\_2171G\|UniProtKB=Q9HNB5 HALSA\|EnsemblGenome=VNG\_0628G\|UniProtKB=Q9HRM7 | dehydrogenase | oxidoreductase | - | 1462 |
|  | **116.** | PC00198 | HALSA\|EnsemblGenome=VNG\_1644G\|UniProtKB=Q9HPG8 HALSA\|EnsemblGenome=VNG\_0935G\|UniProtKB=Q9HQZ4 HALSA\|EnsemblGenome=VNG\_1364G\|UniProtKB=Q9HQ24 HALSA\|EnsemblGenome=VNG\_0829G\|UniProtKB=Q9HR74 HALSA\|EnsemblGenome=VNG\_1404G\|UniProtKB=Q9HPZ3 | reductase | oxidoreductase | - | 958 |
|  | **117.** | GO:0019752 | HALSA\|EnsemblGenome=VNG\_0627G\|UniProtKB=Q9HRM8 | carboxylic acid metabolic process | oxoacid metabolic process | carboxylic acid catabolic process monocarboxylic acid metabolic process carboxylic acid biosynthetic process cellular amino acid metabolic process icosanoid metabolic process | 2128 |
|  | **118.** | GO:0008238 | HALSA\|EnsemblGenome=VNG\_0437C\|UniProtKB=Q9HS25 | exopeptidase activity | peptidase activity | carboxypeptidase activity aminopeptidase activity | 392 |
|  | **119.** | PC00173 | HALSA\|EnsemblGenome=VNG\_2186G\|UniProtKB=Q9HNA0 | nucleotide phosphatase | phosphatase | - | 94 |
|  | **120.** | GO:0004672 | HALSA\|EnsemblGenome=VNG\_1234C\|UniProtKB=Q9HQB9 | protein kinase activity | phosphotransferase activity, alcohol group as acceptor | transmembrane receptor protein kinase activity protein tyrosine kinase activity protein serine/threonine kinase activity cyclin-dependent protein kinase activity | 2695 |
|  | **121.** | GO:0098797 | HALSA\|EnsemblGenome=VNG\_6264G\|UniProtKB=Q9HHR7 HALSA\|EnsemblGenome=VNG\_1632G\|UniProtKB=Q9HPH6 | plasma membrane protein complex | plasma membrane | plasma membrane respiratory chain complex I voltage-gated calcium channel complex | 597 |
|  | **122.** | PC00003 | HALSA\|EnsemblGenome=VNG\_2349G\|UniProtKB=Q9HMW9 HALSA\|EnsemblGenome=VNG\_2358G\|UniProtKB=Q9HMW4 HALSA\|EnsemblGenome=VNG\_0457G\|UniProtKB=Q9HS10 HALSA\|EnsemblGenome=VNG\_2636G\|UniProtKB=Q9HMA2 HALSA\|EnsemblGenome=VNG\_2365G\|UniProtKB=Q9HMW0 HALSA\|EnsemblGenome=VNG\_2344G\|UniProtKB=Q9HMX2 HALSA\|EnsemblGenome=VNG\_2359G\|UniProtKB=Q9HMW3 HALSA\|EnsemblGenome=VNG\_6264G\|UniProtKB=Q9HHR7 HALSA\|EnsemblGenome=VNG\_0452G\|UniProtKB=Q9HS13 HALSA\|EnsemblGenome=VNG\_2363G\|UniProtKB=Q9HMW1 HALSA\|EnsemblGenome=VNG\_0668C\|UniProtKB=Q9HRJ6 HALSA\|EnsemblGenome=VNG\_2343G\|UniProtKB=Q9HMX3 | ATP-binding cassette (ABC) transporter | primary active transporter | - | 1035 |
|  | **123.** | GO:0005975 | HALSA\|EnsemblGenome=VNG\_2102G\|UniProtKB=Q9HNH4 | carbohydrate metabolic process | primary metabolic process | monosaccharide metabolic process carbohydrate biosynthetic process cellular carbohydrate metabolic process carbohydrate catabolic process polysaccharide metabolic process | 1231 |
|  | **124.** | PC00153 | HALSA\|EnsemblGenome=VNG\_0437C\|UniProtKB=Q9HS25 | metalloprotease | protease | - | 754 |
|  | **125.** | PC00020 | HALSA\|EnsemblGenome=VNG\_2587C\|UniProtKB=Q9HME0 | G-protein | protein-binding activity modulator | heterotrimeric G-protein small GTPase | 513 |
|  | **126.** | GO:0051082 | HALSA\|EnsemblGenome=VNG\_6201G\|UniProtKB=Q9HHW1 | unfolded protein binding | protein binding | - | 442 |
|  | **127.** | PC00237 | HALSA\|EnsemblGenome=VNG\_6112H\|UniProtKB=O51984 HALSA\|EnsemblGenome=VNG\_6076H\|UniProtKB=O54590 | viral or transposable element protein | protein class | reverse transcriptase | 233 |
|  | **128.** | PC00211 | HALSA\|EnsemblGenome=VNG\_0961G\|UniProtKB=P13077 HALSA\|EnsemblGenome=VNG\_0960G\|UniProtKB=P61116 HALSA\|EnsemblGenome=VNG\_1009G\|UniProtKB=P13075 HALSA\|EnsemblGenome=VNG\_1008G\|UniProtKB=P61118 HALSA\|EnsemblGenome=VNG\_0962G\|UniProtKB=P13078 | structural protein | protein class | surfactant myelin protein | 426 |
|  | **129.** | PC00009 | HALSA\|EnsemblGenome=VNG\_2473G\|UniProtKB=Q9HMM4 HALSA\|EnsemblGenome=VNG\_6362G\|UniProtKB=Q9HHJ8 | DNA metabolism protein | protein class | DNA polymerase processivity factor DNA methyltransferase exodeoxyribonuclease DNA strand-pairing protein endodeoxyribonuclease DNA topoisomerase DNA glycosylase DNA ligase DNA-directed DNA polymerase DNA helicase replication origin binding protein primase DNA photolyase damaged DNA-binding protein | 911 |
|  | **130.** | PC00072 | HALSA\|EnsemblGenome=VNG\_6201G\|UniProtKB=Q9HHW1 HALSA\|EnsemblGenome=VNG\_0832C\|UniProtKB=Q9HR71 | chaperone | protein class | Hsp100 family chaperone Hsp60 family chaperone Hsp90 family chaperone chaperonin Hsp70 family chaperone | 1294 |
|  | **131.** | PC00095 | HALSA\|EnsemblGenome=VNG\_0451G\|UniProtKB=Q9HS14 HALSA\|EnsemblGenome=VNG\_0458G\|UniProtKB=Q9HS09 | protein-binding activity modulator | protein class | kinase modulator G-protein modulator G-protein phosphatase modulator protease inhibitor | 1060 |
|  | **132.** | PC00227 | HALSA\|EnsemblGenome=VNG\_2361G\|UniProtKB=Q9HMW2 HALSA\|EnsemblGenome=VNG\_0453G\|UniProtKB=Q9HS12 HALSA\|EnsemblGenome=VNG\_0455G\|UniProtKB=Q9HS11 HALSA\|EnsemblGenome=VNG\_1350C\|UniProtKB=Q9HQ34 HALSA\|EnsemblGenome=VNG\_1240G\|UniProtKB=Q9HQB3 HALSA\|EnsemblGenome=VNG\_0321G\|UniProtKB=Q9HSA8 | transporter | protein class | primary active transporter secondary carrier transporter ion channel | 8021 |
|  | **133.** | GO:0070069 | HALSA\|EnsemblGenome=VNG\_6055G\|UniProtKB=O54596 | cytochrome complex | protein-containing complex | respiratory chain complex IV mitochondrial respiratory chain complex III | 91 |
|  | **134.** | GO:1902494 | HALSA\|EnsemblGenome=VNG\_1815G\|UniProtKB=Q9HP42 HALSA\|EnsemblGenome=VNG\_0623G\|UniProtKB=Q9HRN1 | catalytic complex | protein-containing complex | DNA helicase complex oxidoreductase complex ATPase complex catalytic step 2 spliceosome protein serine/threonine phosphatase complex transferase complex peptidase complex dynein complex histone deacetylase complex exosome (RNase complex) endoribonuclease complex | 3337 |
|  | **135.** | GO:0032993 | HALSA\|EnsemblGenome=VNG\_0869G\|UniProtKB=Q9HR45 HALSA\|EnsemblGenome=VNG\_0734G\|UniProtKB=Q9HRE6 | protein-DNA complex | protein-containing complex | DNA replication preinitiation complex pre-replicative complex nuclear telomere cap complex replisome | 236 |
|  | **136.** | GO:0065003 | HALSA\|EnsemblGenome=VNG\_6201G\|UniProtKB=Q9HHW1 | protein-containing complex assembly | protein-containing complex subunit organization | cellular protein-containing complex assembly | 1392 |
|  | **137.** | GO:0046777 | HALSA\|EnsemblGenome=VNG\_1234C\|UniProtKB=Q9HQB9 | protein autophosphorylation | protein phosphorylation | - | 336 |
|  | **138.** | GO:0050794 | HALSA\|EnsemblGenome=VNG\_2115H\|UniProtKB=Q9HNG5 | regulation of cellular process | regulation of biological process | regulation of vesicle-mediated transport regulation of chromosome segregation regulation of cell death regulation of sequestering of calcium ion regulation of establishment or maintenance of cell polarity regulation of cell population proliferation regulation of cellular component movement positive regulation of cellular process regulation of cellular component biogenesis regulation of actin filament-based process regulation of cellular response to growth factor stimulus negative regulation of cellular process regulation of membrane depolarization regulation of cellular metabolic process regulation of secretion by cell regulation of cell adhesion regulation of cellular localization regulation of transmembrane transport regulation of microtubule-based process signal transduction regulation of cell activation regulation of cell division regulation of cell communication regulation of cellular response to stress regulation of conjugation regulation of cellular component organization regulation of cell cycle regulation of cell differentiation | 14489 |
|  | **139.** | GO:0006417 | HALSA\|EnsemblGenome=VNG\_0391C\|UniProtKB=Q9HS60 | regulation of translation | regulation of cellular amide metabolic process | regulation of translational initiation negative regulation of translation positive regulation of translation | 411 |
|  | **140.** | GO:1901700 | HALSA\|EnsemblGenome=VNG\_6201G\|UniProtKB=Q9HHW1 HALSA\|EnsemblGenome=VNG\_1285G\|UniProtKB=Q9HQ80 | response to oxygen-containing compound | response to chemical | response to estradiol cellular response to oxygen-containing compound response to glucose response to gibberellin response to peptide response to lipopolysaccharide response to abscisic acid | 850 |
|  | **141.** | GO:0010243 | HALSA\|EnsemblGenome=VNG\_1285G\|UniProtKB=Q9HQ80 | response to organonitrogen compound | response to nitrogen compound | cellular response to organonitrogen compound ERAD pathway response to peptide | 417 |
|  | **142.** | GO:0006979 | HALSA\|EnsemblGenome=VNG\_6201G\|UniProtKB=Q9HHW1 | response to oxidative stress | response to stress | cellular response to oxidative stress | 292 |
|  | **143.** | GO:0009408 | HALSA\|EnsemblGenome=VNG\_6201G\|UniProtKB=Q9HHW1 | response to heat | response to stress | cellular response to heat | 158 |
|  | **144.** | GO:0006970 | HALSA\|EnsemblGenome=VNG\_6201G\|UniProtKB=Q9HHW1 | response to osmotic stress | response to stress | cellular response to osmotic stress | 101 |
|  | **145.** | GO:0009152 | HALSA\|EnsemblGenome=VNG\_0997G\|UniProtKB=Q9HQU8 | purine ribonucleotide biosynthetic process | ribonucleotide biosynthetic process | ATP biosynthetic process cGMP biosynthetic process IMP biosynthetic process | 483 |
|  | **146.** | PC00147 | HALSA\|EnsemblGenome=VNG\_1980C\|UniProtKB=Q9HNR6 | RNA processing factor | RNA metabolism protein | mRNA polyadenylation factor mRNA capping factor RNA splicing factor | 1483 |
|  | **147.** | PC00259 | HALSA\|EnsemblGenome=AAC82884\|UniProtKB=O52004 HALSA\|EnsemblGenome=VNG\_0869G\|UniProtKB=Q9HR45 HALSA\|EnsemblGenome=VNG\_0734G\|UniProtKB=Q9HRE6 | general transcription factor | RNA metabolism protein | - | 455 |
|  | **148.** | PC00033 | HALSA\|EnsemblGenome=VNG\_2475C\|UniProtKB=Q9HMM2 | RNA methyltransferase | RNA metabolism protein | - | 164 |
|  | **149.** | GO:0008175 | HALSA\|EnsemblGenome=VNG\_1980C\|UniProtKB=Q9HNR6 HALSA\|EnsemblGenome=VNG\_2475C\|UniProtKB=Q9HMM2 | tRNA methyltransferase activity | RNA methyltransferase activity | - | 67 |
|  | **150.** | GO:0003688 | HALSA\|EnsemblGenome=VNG\_1224G\|UniProtKB=Q9HQC7 | DNA replication origin binding | sequence-specific double-stranded DNA binding | - | 30 |
|  | **151.** | GO:0006637 | HALSA\|EnsemblGenome=VNG\_0997G\|UniProtKB=Q9HQU8 | acyl-CoA metabolic process | sulfur compound metabolic process | - | 175 |
|  | **152.** | GO:0006352 | HALSA\|EnsemblGenome=AAC82884\|UniProtKB=O52004 HALSA\|EnsemblGenome=VNG\_0869G\|UniProtKB=Q9HR45 HALSA\|EnsemblGenome=VNG\_0734G\|UniProtKB=Q9HRE6 | DNA-templated transcription, initiation | transcription, DNA-templated | transcription initiation from RNA polymerase II promoter | 194 |
|  | **153.** | GO:0016765 | HALSA\|EnsemblGenome=VNG\_1882G\|UniProtKB=Q9HNZ1 | transferase activity, transferring alkyl or aryl (other than methyl) groups | transferase activity | glutathione transferase activity prenyltransferase activity | 606 |
|  | **154.** | GO:0016746 | HALSA\|EnsemblGenome=VNG\_2102G\|UniProtKB=Q9HNH4 | acyltransferase activity | transferase activity | acyltransferase activity, transferring groups other than amino-acyl groups | 1359 |
|  | **155.** | GO:0004659 | HALSA\|EnsemblGenome=VNG\_1781C\|UniProtKB=Q9HP67 HALSA\|EnsemblGenome=VNG\_1458G\|UniProtKB=Q9HPV3 HALSA\|EnsemblGenome=VNG\_2137G\|UniProtKB=Q9HNE5 | prenyltransferase activity | transferase activity, transferring alkyl or aryl (other than methyl) groups | - | 118 |
|  | **156.** | GO:0016773 | HALSA\|EnsemblGenome=VNG\_1900C\|UniProtKB=Q9HNX7 | phosphotransferase activity, alcohol group as acceptor | transferase activity, transferring phosphorus-containing groups | protein kinase activity | 3211 |
|  | **157.** | GO:0016301 | HALSA\|EnsemblGenome=VNG\_1900C\|UniProtKB=Q9HNX7 HALSA\|EnsemblGenome=VNG\_6316G\|UniProtKB=Q48295 | kinase activity | transferase activity, transferring phosphorus-containing groups | carbohydrate kinase activity lipid kinase activity protein kinase activity nucleoside diphosphate kinase activity | 3397 |
|  | **158.** | PC00155 | HALSA\|EnsemblGenome=VNG\_1568G\|UniProtKB=Q9HPM0 | methyltransferase | transferase | - | 782 |
|  | **159.** | PC00111 | HALSA\|EnsemblGenome=VNG\_1066C\|UniProtKB=Q9HQP4 | glycosyltransferase | transferase | - | 1360 |
|  | **160.** | PC00038 | HALSA\|EnsemblGenome=VNG\_0654C\|UniProtKB=Q9HRK6 | acetyltransferase | transferase | - | 493 |
|  | **161.** | PC00137 | HALSA\|EnsemblGenome=VNG\_6316G\|UniProtKB=Q48295 | kinase | transferase | carbohydrate kinase nucleotide kinase amino acid kinase | 726 |
|  | **162.** | PC00042 | HALSA\|EnsemblGenome=VNG\_1781C\|UniProtKB=Q9HP67 | acyltransferase | transferase | - | 542 |
|  | **163.** | PC00202 | HALSA\|EnsemblGenome=VNG\_2514G\|UniProtKB=Q9HMJ5 HALSA\|EnsemblGenome=VNG\_1190G\|UniProtKB=P09737 | ribosomal protein | translational protein | - | 1592 |
|  | **164.** | GO:0006414 | HALSA\|EnsemblGenome=VNG\_0391C\|UniProtKB=Q9HS60 | translational elongation | translation | - | 1395 |
|  | **165.** | GO:0002181 | HALSA\|EnsemblGenome=VNG\_0391C\|UniProtKB=Q9HS60 | cytoplasmic translation | translation | cytoplasmic translational initiation | 350 |
|  | **166.** | PC00265 | HALSA\|EnsemblGenome=VNG\_1234C\|UniProtKB=Q9HQB9 HALSA\|EnsemblGenome=VNG\_0916G\|UniProtKB=Q9HR10 | histidine kinase receptor of two-component system | transmembrane signal receptor | - | 154 |
|  | **167.** | GO:0022804 | HALSA\|EnsemblGenome=VNG\_6264G\|UniProtKB=Q9HHR7 | active transmembrane transporter activity | transmembrane transporter activity | secondary active transmembrane transporter activity active ion transmembrane transporter activity | 1461 |
|  | **168.** | PC00068 | HALSA\|EnsemblGenome=VNG\_1462G\|UniProtKB=Q9HPV0 | primary active transporter | transporter | ATP-binding cassette (ABC) transporter ATP synthase | 1616 |
|  | **169.** | PC00133 | HALSA\|EnsemblGenome=VNG\_1544G\|UniProtKB=Q9HPN8 | ion channel | transporter | voltage-gated ion channel ligand-gated ion channel | 1075 |
|  | **170.** | GO:0030488 | HALSA\|EnsemblGenome=VNG\_1980C\|UniProtKB=Q9HNR6 HALSA\|EnsemblGenome=VNG\_2475C\|UniProtKB=Q9HMM2 | tRNA methylation | tRNA modification | - | 81 |

  


|  |  |
| --- | --- |
| Hits 1-170 of 170 | [ page: (1) ] |

  
  
  
  
  


About   |  
Release Information   |  
Contact Us   |  
System Requirements   |  
Privacy Policy   |  
Disclaimer

© Copyright 2022 Paul Thomas All Rights Reserved.
