# Supplementary material for: Low Salt Influences Archaellum-Based Motility, Glycerol Metabolism, and Gas Vesicles Biogenesis in Halobacterium salinarum
Source: Microorganisms. 2022 Dec 10;10(12):2442. doi: 10.3390/microorganisms10122442 (PMC9786353; doi:10.3390/microorganisms10122442)
Supplement: Supplementary file 1 [file microorganisms-10-02442-s001.zip › microorganisms-2047039-File S1.zip!/list-283-diff-express-genes-PANTHER-category-v03.html]

The mission of the PANTHER knowledgebase is to support biomedical and other research by providing **comprehensive information about the evolution of protein-coding gene families**, particularly protein phylogeny, function and genetic variation impacting that function. More...

|  |  |  |
| --- | --- | --- |
|  | All Genes and orthologs Pathways PANTHER families Ontology terms | Go |

|  |  |  |  |  |  |  |  |  |  |  |  |  |  |  |  |  |  |
| --- | --- | --- | --- | --- | --- | --- | --- | --- | --- | --- | --- | --- | --- | --- | --- | --- | --- |
|  | Home |  | About |  | Data Version |  | Tools |  | API/Services |  | Publications |  | Workspace |  | Downloads |  | FAQ/Help/Tutorial |

|  |  |  |  |  |  |
| --- | --- | --- | --- | --- | --- |
|  | Login |  | Register |  | Contact us |

|  |  |  |
| --- | --- | --- |
|  | **PANTHER17.0 Released.** | Current Release: **PANTHER 17.0**  \|  **15,619** family phylogenetic trees  \|  **143** species  \|  News   Whole genome function views |

Analysis Summary: Please report in publication 


**Analysis Type:** PANTHER Overrepresentation Test (Released 20220712)

---

**Annotation Version and Release Date:** PANTHER version 17.0 Released 2022-02-22

---

|  |  |  |
| --- | --- | --- |
| **Analyzed List:** | Client Text Box Input (Halobacterium salinarum) | Change |
| --- | | |
| **Reference List:** | Halobacterium salinarum (all genes in database) | Change |

---

**Annotation Data Set:** 

 GO biological process complete
 GO molecular function complete
 GO cellular component complete
 PANTHER Pathways
 PANTHER GO-Slim Molecular Function
 PANTHER GO-Slim Biological Process
 PANTHER GO-Slim Cellular Component
 PANTHER Protein Class
 Reactome pathways

---

**Test Type:** 
Fisher's Exact  
Binomial

---

**Correction:** 
Calculate False Discovery Rate  
Use the Bonferroni correction for multiple testing    
No correction

  
Results

  


|
|  |


|  |  |  |
| --- | --- | --- |
|  | Reference list | Client Text Box Input |
| Uniquely Mapped IDS: | 2423 out of 2423 | 237 out of 237 |
| Unmapped IDs: | 0 | 46 |
| Multiple mapping information: | 0 | 0 |

  
Export  Table  XML with user input ids  JSON with user input ids
  View:
-- Please select a chart to display --
Multiple Pie Chart
Bar Chart of Gene Count
Overlaid Area Chart of Difference
Bar Chart of Difference
  
  
Displaying only results for uncorrected P < 0.05, click here to display all results
  
  

|  |  |  |  |  |  |  |  |
| --- | --- | --- | --- | --- | --- | --- | --- |
|  | Halobacterium salinarum (REF) | Client Text Box Input ( Hierarchy  NEW! ) | | | | | |
| PANTHER Protein Class | # | # | expected | Fold Enrichment | +/- | raw P value |  |
| structural protein | 10 | 5 | .98 | 5.11 | + | 7.67E-03 |  |
| ATP-binding cassette (ABC) transporter | 52 | 12 | 5.09 | 2.36 | + | 1.17E-02 |  |
| primary active transporter | 70 | 13 | 6.85 | 1.90 | + | 4.64E-02 |  |
| translational protein | 87 | 2 | 8.51 | .24 | - | 2.13E-02 |  |

About   |  
Release Information   |  
Contact Us   |  
System Requirements   |  
Privacy Policy   |  
Disclaimer

� Copyright 2022 Paul Thomas All Rights Reserved.
